# Supplementary figures and images for: Genotypic and phenotypic characterization of thermo-sensitive genic male sterile (TGMS) rice lines using simple sequence repeat (SSR) markers and population structure analysis
Source: PeerJ. 2025 May 8;13:e18975. doi: 10.7717/peerj.18975 (PMC12066105; doi:10.7717/peerj.18975)

Supplementary figure 1. Heat map representing the Euclidean distance between the TGMS

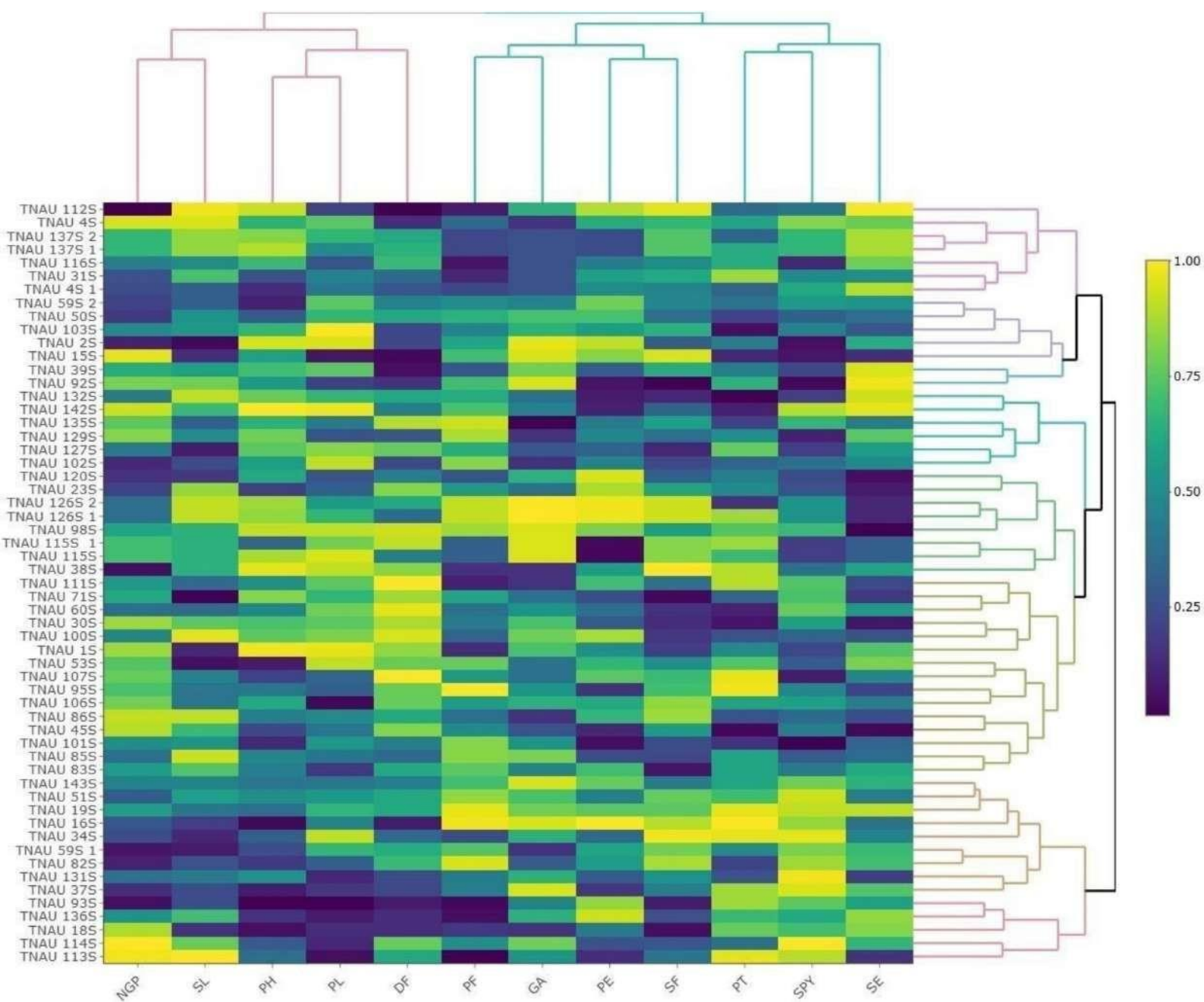

Supplement: Supplemental Information 1 [file peerj-13-18975-s001.pdf]
