# Supplementary material for: Genotypic and phenotypic characterization of thermo-sensitive genic male sterile (TGMS) rice lines using simple sequence repeat (SSR) markers and population structure analysis
Source: PeerJ. 2025 May 8;13:e18975. doi: 10.7717/peerj.18975 (PMC12066105; doi:10.7717/peerj.18975)
Supplement: Supplemental Information 2 [file peerj-13-18975-s002.docx]

Supplementary table 1. List of TGMS lines along with pedigree.

| **S. No.** | **TGMS line** | **Pedigree** |
| --- | --- | --- |
| 1 | TNAU 1S | Selection from CBDHTS 0235 |
| 2 | TNAU 2S | Selection from CBTS 0282 (TS29/IR68281B-400) |
| 3 | TNAU 4S | Selection from GD 98049-1-1(CBGD0502) |
| 4 | TNAU 4S-1 | Selection from GD 98049-1-1(CBGD0502) |
| 5 | TNAU 15S | Selection from IR 73827 – 23 S |
| 6 | TNAU 16S | Selection from IR 73824 S |
| 7 | TNAU 18S | Selection from IR 75589-41-13-17-15-22S |
| 8 | TNAU 19S | Selection from IR 75589-41-13-17-15-3S |
| 9 | TNAU 23S | TS 16 x IR 36 -1-2-122 |
| 10 | TNAU 30S | TS 29 x IR682818-400-4-2-3-1 |
| 11 | TNAU 31S | TS 16 x MRST 9-292-2-1 |
| 12 | TNAU 34S | TS 29 x IR 68281B-400-2-6 |
| 13 | TNAU 37S | TS 29 x IR 68281B-400-2-1 |
| 14 | TNAU 38S | TS 29 x IR 68281B-25-5-2 |
| 15 | TNAU 39S | TS 29 x IR 68281B-400-2-3-2 |
| 16 | TNAU 45S | Selection from GD 99017-17 |
| 17 | TNAU 50S | Selection from GD 99017-10 |
| 18 | TNAU 51S | Selection from GD 98049-1-49 |
| 19 | TNAU 53S | Selection from GD 98049-29 |
| 20 | TNAU 59S-1 | Mutant from ADT 39 at Thenkasi |
| 21 | TNAU 59S-2 | Mutant from ADT 39 at Thenkasi |
| 22 | TNAU 60S | Spontaneous mutant from PMK 3 (Pet Type) |
| 23 | TNAU 71S | Selection from DRR 29S |
| 24 | TNAU 82S | Selection from GDR 9 S |
| 25 | TNAU 83S | TNAU 45S-2 |
| 26 | TNAU 85S | TS 29 100GY |
| 27 | TNAU 86S | Spontaneous mutant MS 3 |
| 28 | TNAU 92S | Selection from DRR 23 S |
| 29 | TNAU 93S | Selection from DRR 28 S |
| 30 | TNAU 95S | Selection from TNAU 65 S (TS 29/IR 62917-2-3-2-1-1) |
| 31 | TNAU 98S | GD98049-3-29-1 |
| 32 | TNAU 100S | ADT 39 100GY |
| 33 | TNAU 101S | DRR 23S-1 |
| 34 | TNAU 102S | TS29-1 |
| 35 | TNAU 103S | CBTS0282-27-4-15-15 |
| 36 | TNAU 106S | CBTS0282-27-4-12-15 |
| 37 | TNAU 107S | GD98029-29 |
| 38 | TNAU 111S | TS29150GY-3 |
| 39 | TNAU 112S | MS4/TNAU51-1 |
| 40 | TNAU 113S | TNAU 4S-7-2/BPT5204 |
| 41 | TNAU 114S | TNAU 4S-2/BPT 5204 |
| 42 | TNAU 115S | TS 06-207-1-28 |
| 43 | TNAU 115S-1 | TS 06-207-1-28 |
| 44 | TNAU 116S | CBTS 282-6-22-1 |
| 45 | TNAU 120S | CBTS 0282-282-7 |
| 46 | TNAU 126S-1 | TNAU 15S/Sona Mashuri -2 |
| 47 | TNAU 126S-2 | TNAU 15S/Sona Mashuri -2 |
| 48 | TNAU 127S | TNAU 15S/Sona Mashuri -3 |
| 49 | TNAU 129S | MS 2S-2 |
| 50 | TNAU 131S | CO 49/TS 29 (F4 27S) |
| 51 | TNAU 132S | CO 49/TS 29 (F4 19S-1) |
| 52 | TNAU 135S | TS29/CO 49 (F4 17S) |
| 53 | TNAU 136S | TS 06-182-2 |
| 54 | TNAU 137S-1 | TNAU 4S-1-2/CB06-564 |
| 55 | TNAU 137S-2 | TNAU 4S-1-2/CB06-564 |
| 56 | TNAU 142S | TNAU 19S-1 |
| 57 | TNAU 143S | TNAU 19S-2 |
