# Supplementary material for: Genotypic and phenotypic characterization of thermo-sensitive genic male sterile (TGMS) rice lines using simple sequence repeat (SSR) markers and population structure analysis
Source: PeerJ. 2025 May 8;13:e18975. doi: 10.7717/peerj.18975 (PMC12066105; doi:10.7717/peerj.18975)
Supplement: Supplemental Information 3 [file peerj-13-18975-s003.docx]

Supplementary table 2. The list of polymorphic markers along with their sequence, chromosome number and annealing temperature.

| **S.no** | **Marker** | **Fp sequence (5’-3’)** | **Rp sequence (5’-3’)** | **Chromosome number** | **Annealing temperature** | **Base pairs lengths** |
| --- | --- | --- | --- | --- | --- | --- |
| 1 | RM319 | ATCAAGGTACCTAGACCACCAC | TCCTGGTGCAGCTATGTCTG | 1 | 55 | 134-175 |
| 2 | RM22597 | GACCAATATGGAAGAACCTGTGC | CTAAGAAGTGGTAGACTTGCCAATGC | 5 | 55 | 249-255 |
| 3 | RM3533 | TTCCAACCTGTCAGGGAATC | CATTTCCCTTCCCTCTCCTC | 9 | 55 | 120-125 |
| 4 | RM7653 | AATTCGTCCCCGTCTCCTAC | GAATTCCAGCTCTTTGACCG | 5 | 50 | 121-125 |
| 5 | RM4601 | CATACATGTGAACCTGACTG | CTAGCTTAGCATCTCCTCAA | 11 | 55 | 115-120 |
| 6 | RM13912 | TTCCGTGGAAGATTCTCGATACC | GCTAGCCTACACAACGCAAATCC | 2 | 55 | 185-195 |
| 7 | RM36 | CAACTATGCACCATTGTCGC | GTACTCCACAAGACCGTACC | 3 | 55 | 180-197 |
| 8 | RM81 | GAGTGCTTGTGCAAGATCCA | CTTCTTCACTCATGCAGTTC | 3 | 55 | 105-120 |
| 9 | RM 1018 | ATCTTGTCCCACTGCACCAC | TGTGACTGCTTTTCTGTCGC | 4 | 55 | 160-170 |
| 10 | RM 286 | GGCTTCATCTTTGGCGAC | CCGGATTCACGAGATAAACTC | 11 | 55 | 100-110 |
| 11 | RM1896 | GGACAGGGTAAAGTGTTAGA | CCTAAGACCTATCAACTCCA | 9 | 55 | 102-108 |
| 12 | RM5709 | CTGAATTTATTATAGGACGGAAG | CATAGTATTGGATTGGACACG | 4 | 55 | 110-113 |
| 13 | RM310 | CCAAAACATTTAAAATATCATG | GCTTGTTGGTCATTACCATTC | 8 | 55 | 100-105 |
| 14 | RM559 | ACGTACACTTGGCCCTATGC | ATGGGTGTCAGTTTGCTTCC | 4 | 55 | 160-180 |
| 15 | RM16559 | CCTGGAACCTGGAGGTGTTCTCG | GTCGTGGACGATTTCTTCGTCAGC | 4 | 55 | 197-210 |
| 16 | RM420 | GGACAGAATGTGAAGACAGTCG | ACTAATCCACCAACGCATCC | 7 | 55 | 190-210 |
| 17 | RM282 | CTGTGTCGAAAGGCTGCAC | CAGTCCTGTGTTGCAGCAAG | 3 | 55 | 136-140 |
| 18 | RM210 | TCACATTCGGTGGCATTG | CGAGGATGGTTGTTCACTTG | 8 | 55 | 125-145 |
| 19 | RM125 | ATCAGCAGCCATGGCAGCGACC | AGGGGATCATGTGCCGAAGGCC | 7 | 55 | 123-127 |
| 20 | RM5352 | GGAACTAAACATGGTGCAAG | ACCAGATCACATGAAGAGGA | 10 | 50 | 103-125 |
| 21 | RM4455 | CTCTCAAAGAACTAGGACTC | GAGAAGGTATGATAACCAAT | 10 | 55 | 105-120 |
| 22 | RM157 | CCTCCTCCTCACGAATCCCGCC | GGGCTTCTTCTCCGCCGGCTTC | 3 | 55 | 103-115 |
| 23 | RM195 | AGAAAGAGAGGCCGTCGGCGGC | GGGCTCACCCCCAAACCTGCAG | 8 | 61 | 305-370 |
| 24 | RM5704 | AAAAGTTTTGAATAAAACGAATG | ATGTGATTCTCCAAGCAGAG | 11 | 55 | 198-210 |
| 25 | RM184 | ATCCCATTCGCCAAAACCGGCC | TGACACTTGGAGAGCGGTGTGG | 10 | 55 | 120-125 |
| 26 | RM433 | TGCGCTGAACTAAACACAGC | AGACAAACCTGGCCATTCAC | 8 | 55 | 224-245 |
| 27 | RM423 | AGCACCCATGCCTTATGTTG | CCTTTTTCAGTAGCCCTCCC | 2 | 55 | 210-273 |
| 28 | RM561 | GAGCTGTTTTGGACTACGGC | GAGTAGCTTTCTCCCACCCC | 2 | 55 | 175-190 |
| 29 | RM511 | CTTCGATCCGGTGACGAC | AACGAAAGCGAAGCTGTCTC | 12 | 55 | 130-145 |
| 30 | RM5931 | CTCTCCTGCCTGACAAAAGC | GTTTTAGCGGATGTGGCATC | 1 | 50 | 195-202 |
| 31 | RM7403 | GCTGGTGCACACTCGACTC | GACCTGCCTGAGAAGCTGAG | 3 | 50 | 96-105 |
| 32 | RM349 | TTGCCATTCGCGTGGAGGCG | GTCCATCATCCCTATGGTCG | 4 | 55 | 136-160 |
| 33 | RM3917 | GGAACTAAACATGGTGCAAG | ACCAGATCACATGAAGAGGA | 10 | 50 | 225-246 |
| 34 | RM521 | GGGATTTGCAGTGAGCTAGC | TTCCCTTATTCCTGCTCTCC | 2 | 55 | 205-260 |
| 35 | RM552 | CGCAGTTGTGGATTTCAGTG | TGCTCAACGTTTGACTGTCC | 11 | 55 | 195-210 |
| 36 | RM129 | TCTCTCCGGAGCCAAGGCGAGG | CGAGCCACGACGCGATGTACCC | 1 | 55 | 190-205 |
| 37 | RM335 | GTACACACCCACATCGAGAAG | GCTCTATGCGAGTATCCATGG | 4 | 55 | 188-205 |
| 38 | RM457 | CTCCAGCATGGCCTTTCTAC | ACCTGATGGTCAAAGATGGG | 11 | 55 | 150-180 |
| 39 | RM427 | TCACTAGCTCTGCCCTGACC | TGATGAGAGTTGGTTGCGAG | 7 | 55 | 191-200 |
| 40 | RM205 | CTGGTTCTGTATGGGAGCAG | CTGGCCCTTCACGTTTCAGTG | 9 | 55 | 80-110 |
| 41 | RM8263 | TTTGCTGTCCCTTTGTTC | TGCAATTCAAAGTCTTAGGG | 7 | 55 | 193-205 |
| 42 | RM1337 | GTGCAATGCTGAGGAGTATC | CTGAGAATCTGGAGTGCTTG | 12 | 55 | 90-110 |
| 43 | RM346 | CGAGAGAGCCCATAACTACG | ACAAGACGACGAGGAGGGAC | 7 | 55 | 160.-175 |

*Fp- Forward primer; Rp- Reverse primer
