# Supplementary material for: Genotypic and phenotypic characterization of thermo-sensitive genic male sterile (TGMS) rice lines using simple sequence repeat (SSR) markers and population structure analysis
Source: PeerJ. 2025 May 8;13:e18975. doi: 10.7717/peerj.18975 (PMC12066105; doi:10.7717/peerj.18975)

Supplementary file 1. Gel images for Scoring of the SSR markers

RM22597
Chromosome -8

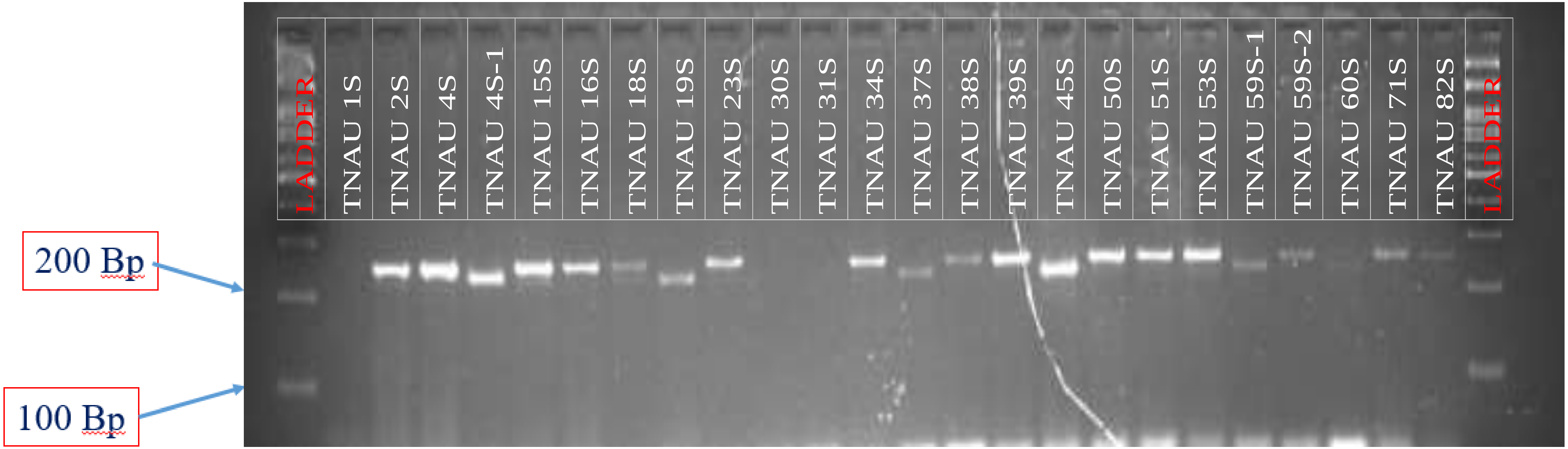

RM22597
Chromosome -8

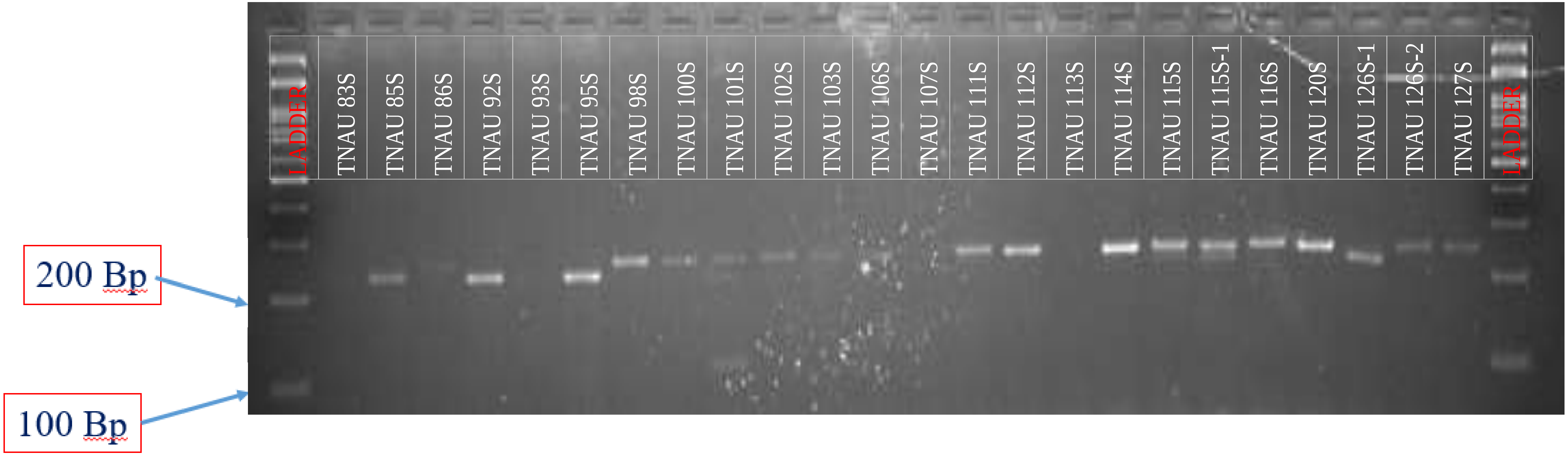

RM3533
Chromosome-9

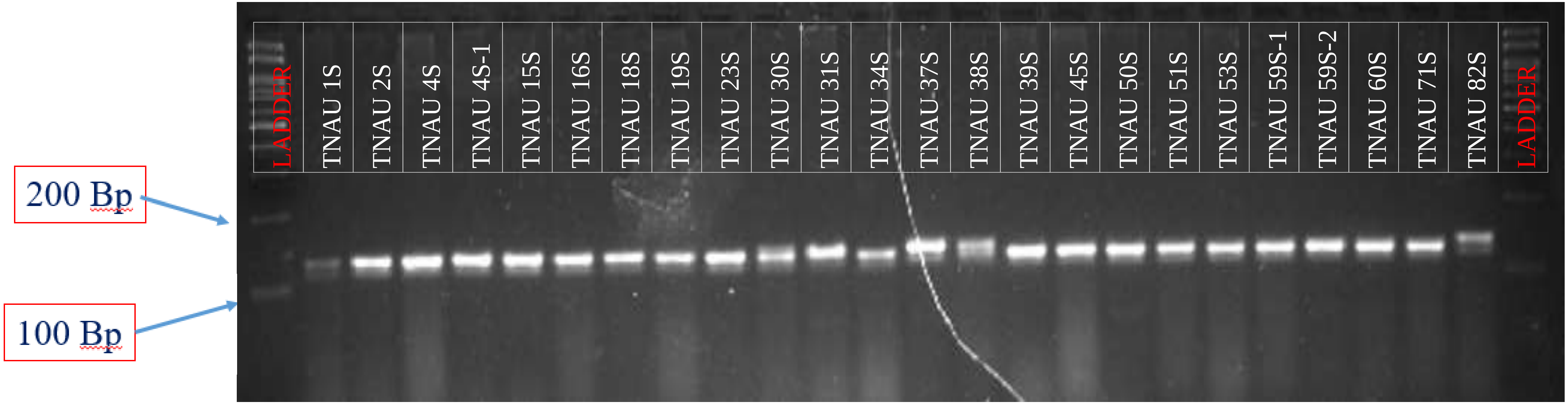

RM3533
Chromosome-9

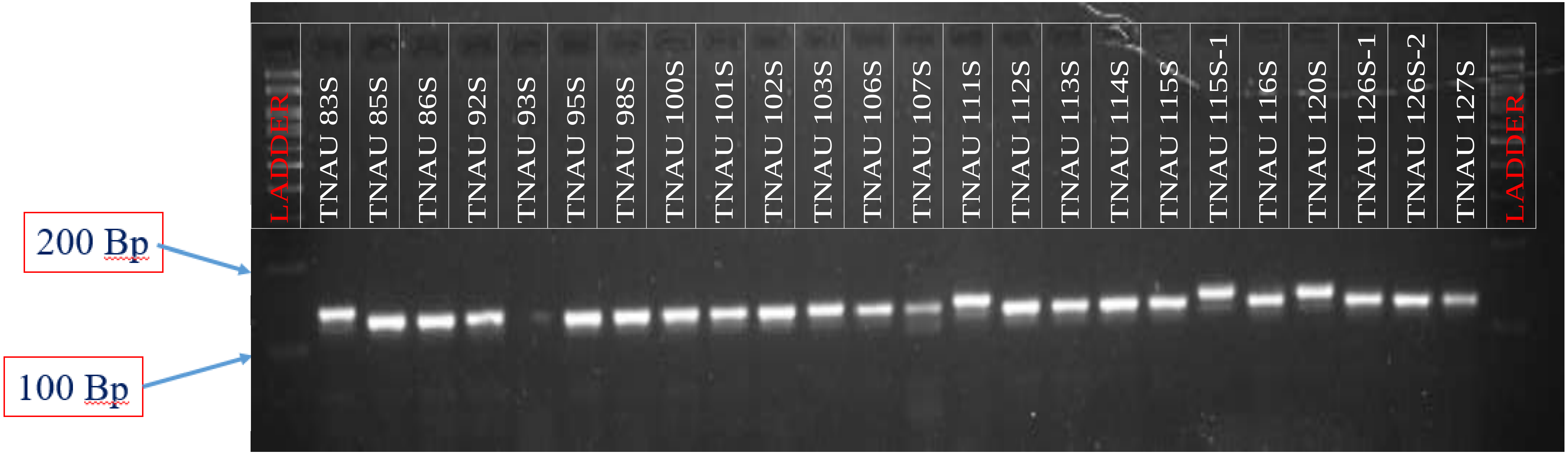

RM3533
Chromosome-9

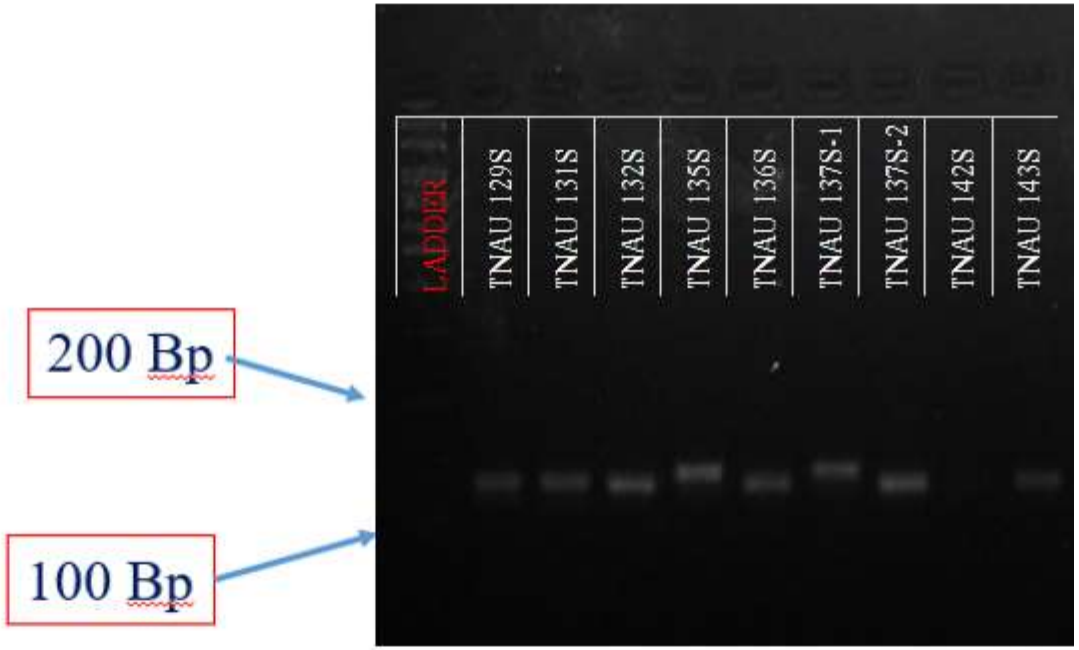

RM7653
Chromosome-5

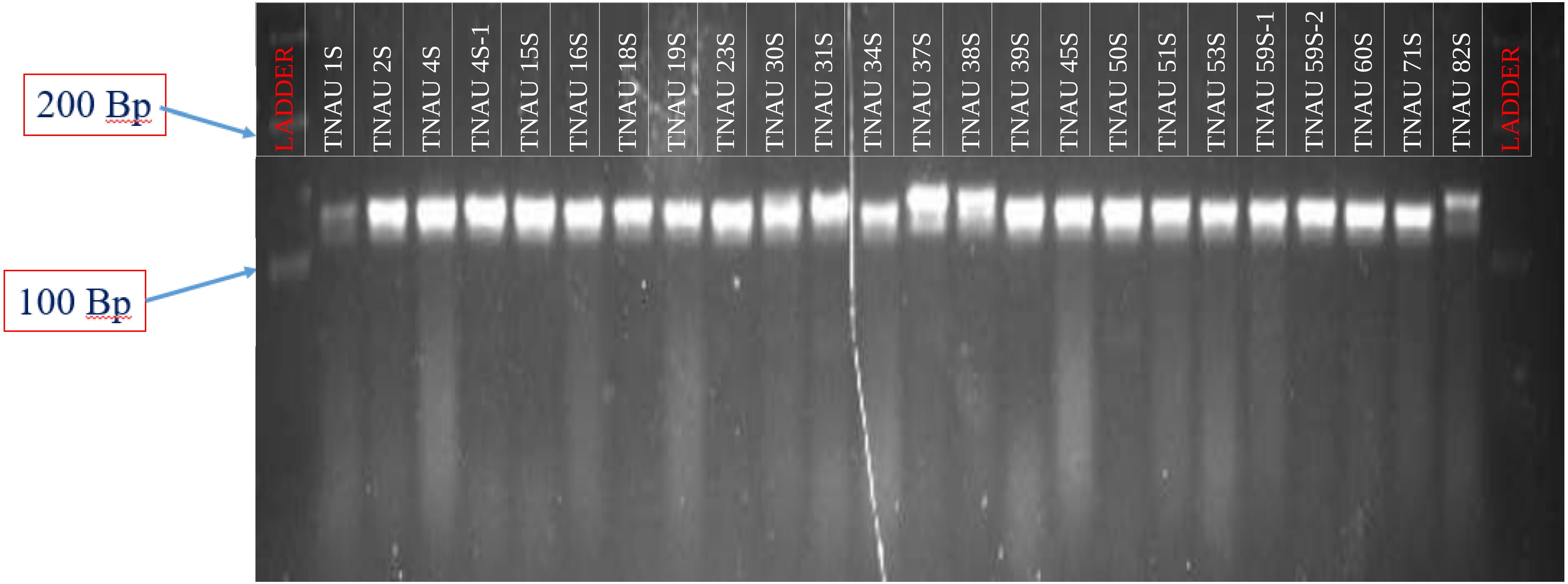

RM7653
Chromosome-5

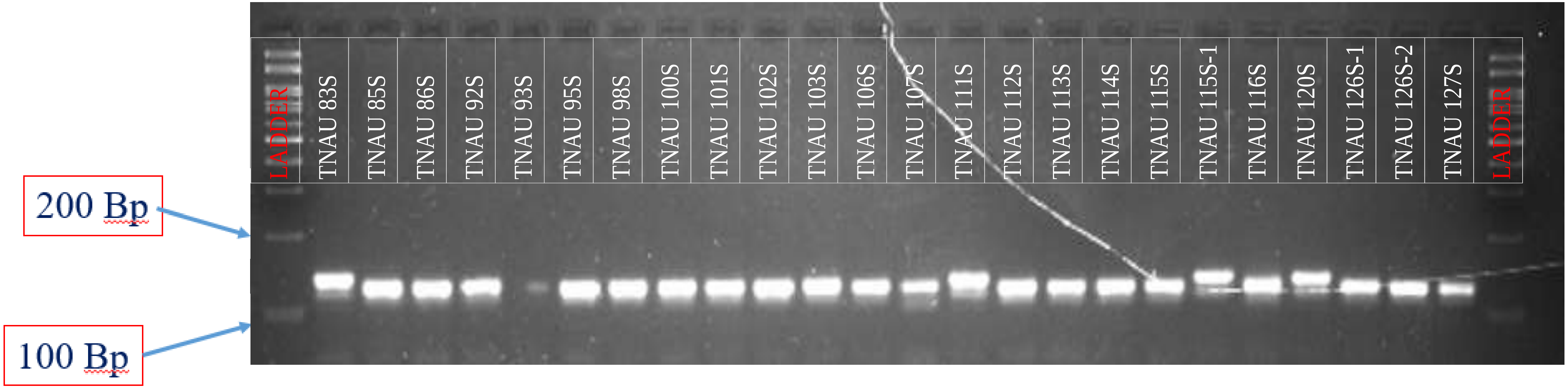

RM7653
Chromosome-5

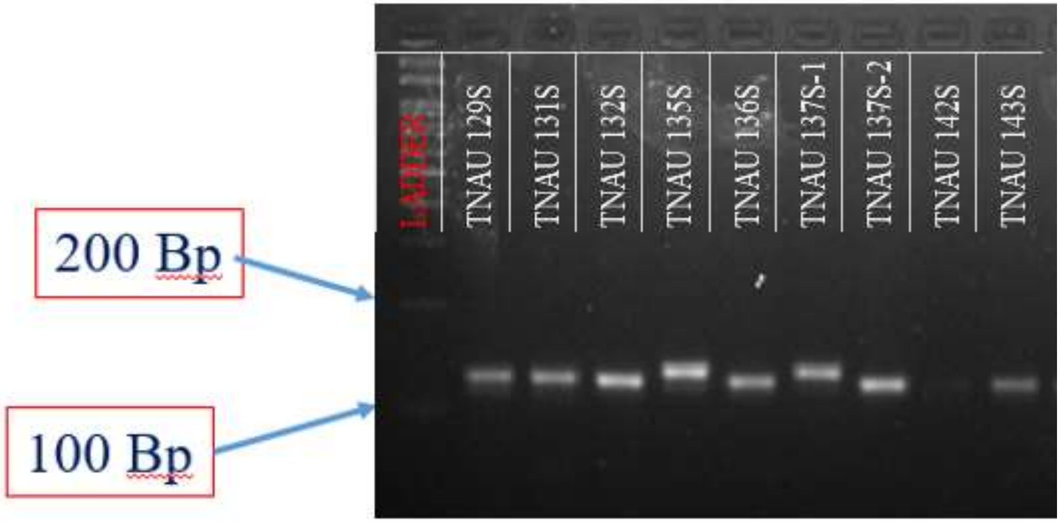

RM4601
Chromosome-11

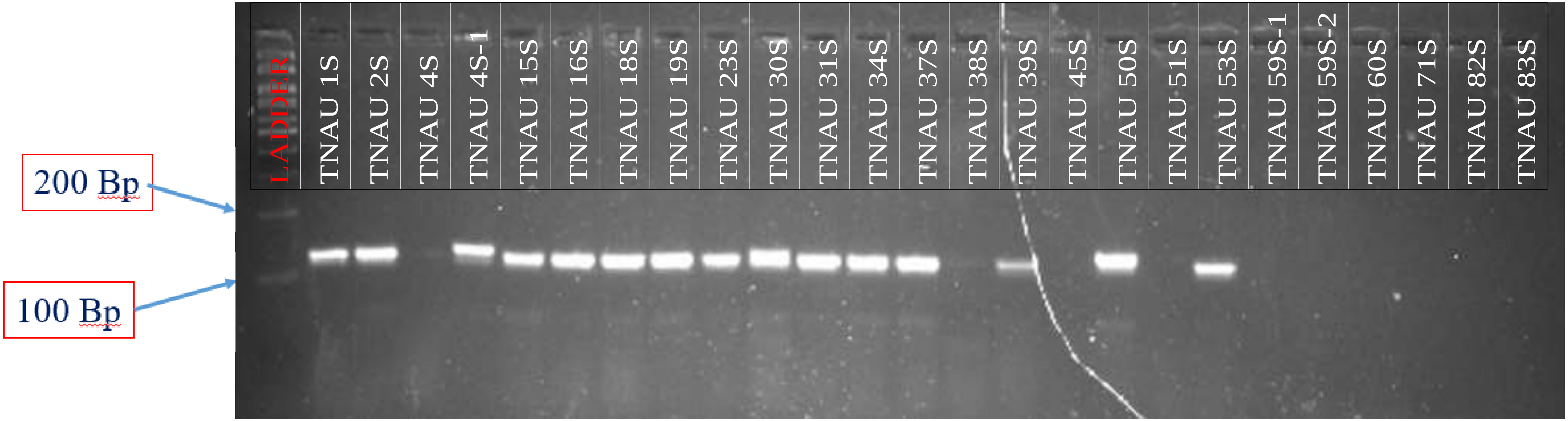

RM4601
Chromosome-11

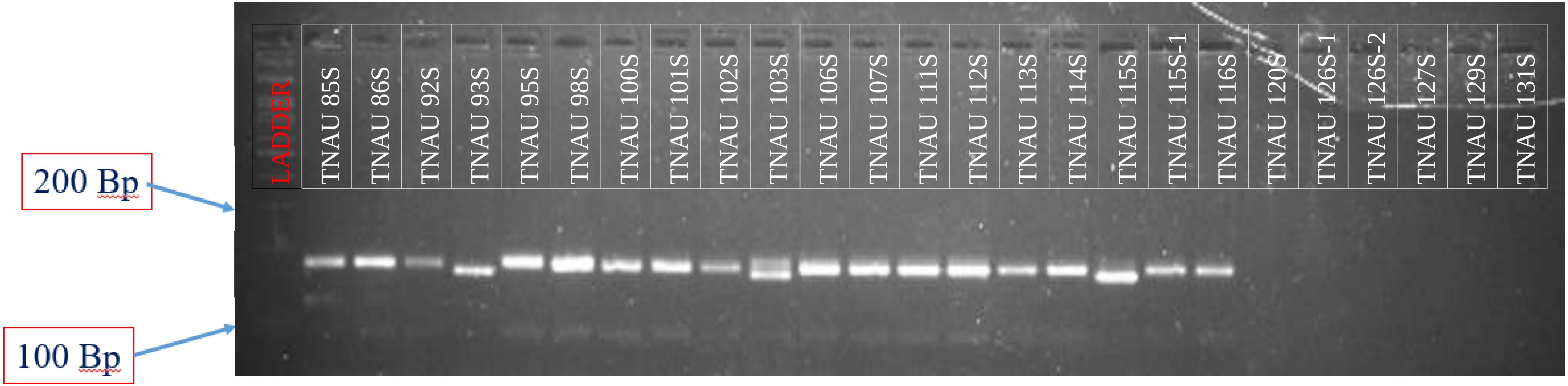

RM4601
Chromosome-11

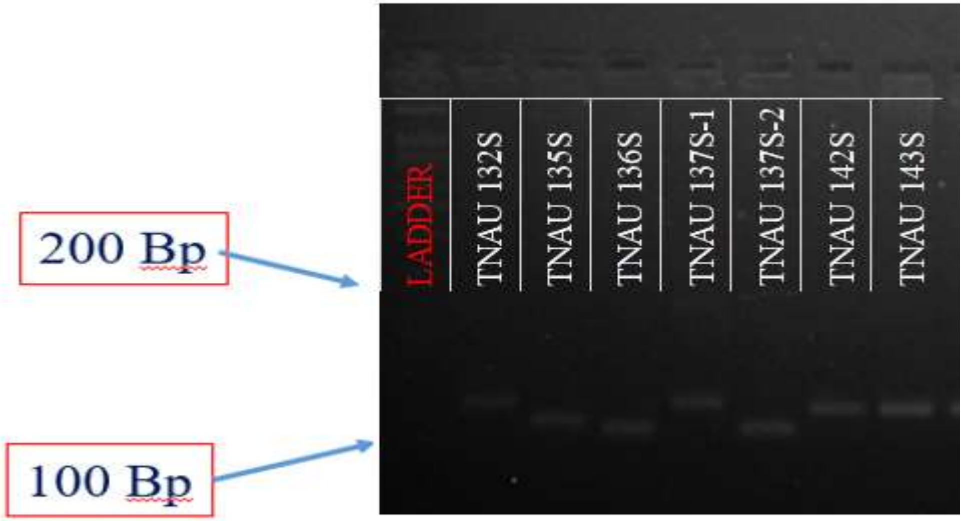

RM13912
Chromosome-2

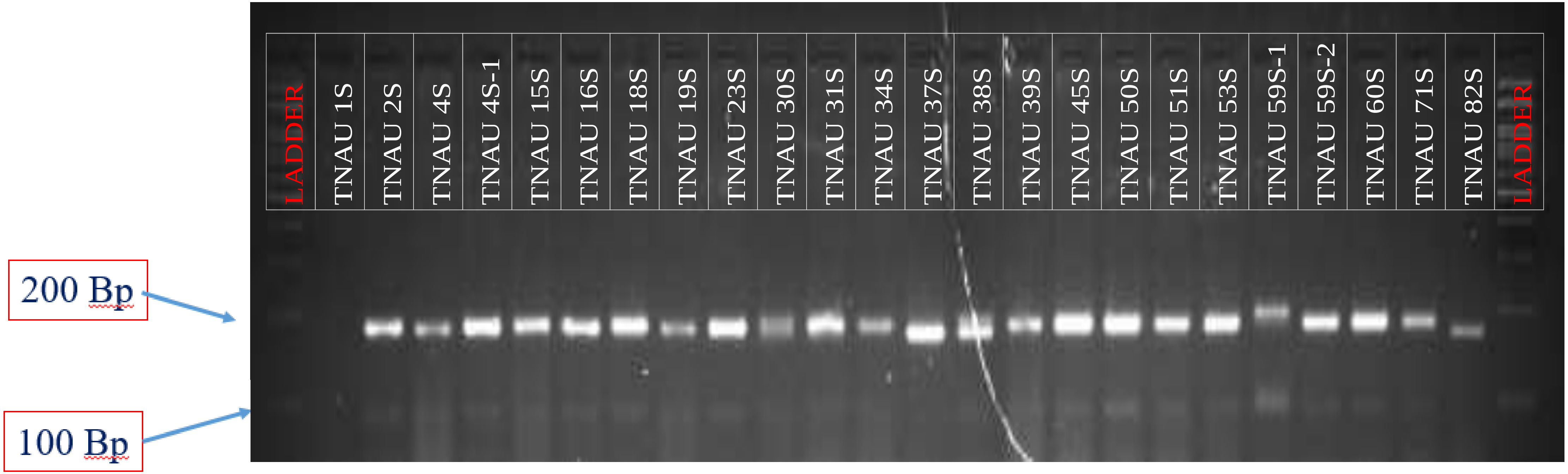

RM13912
Chromosome-2

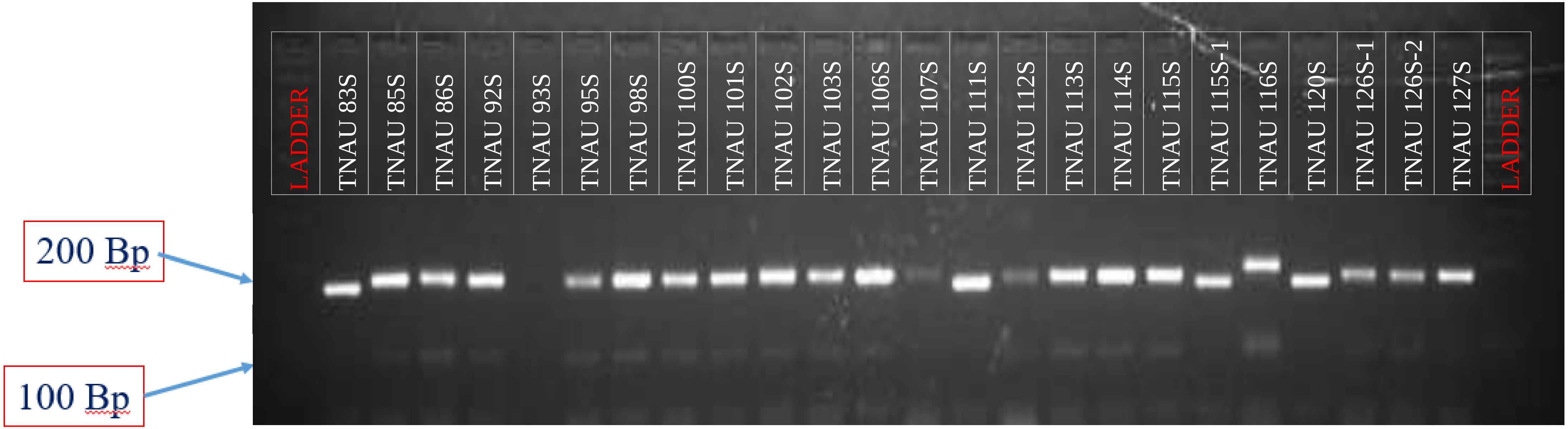

RM13912
Chromosome-2

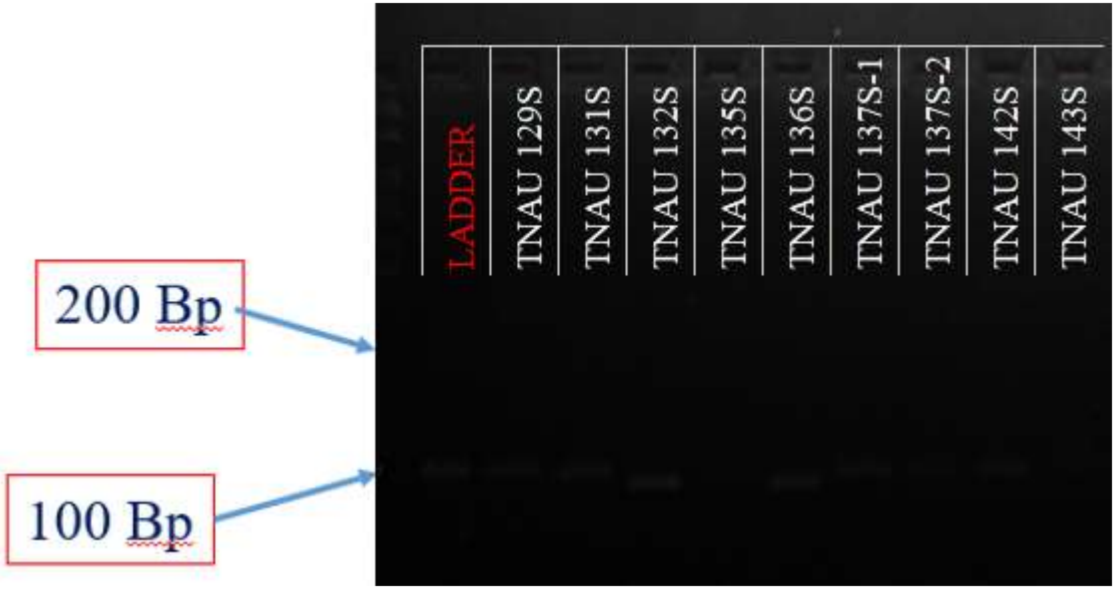

RM36
Chromosome-1

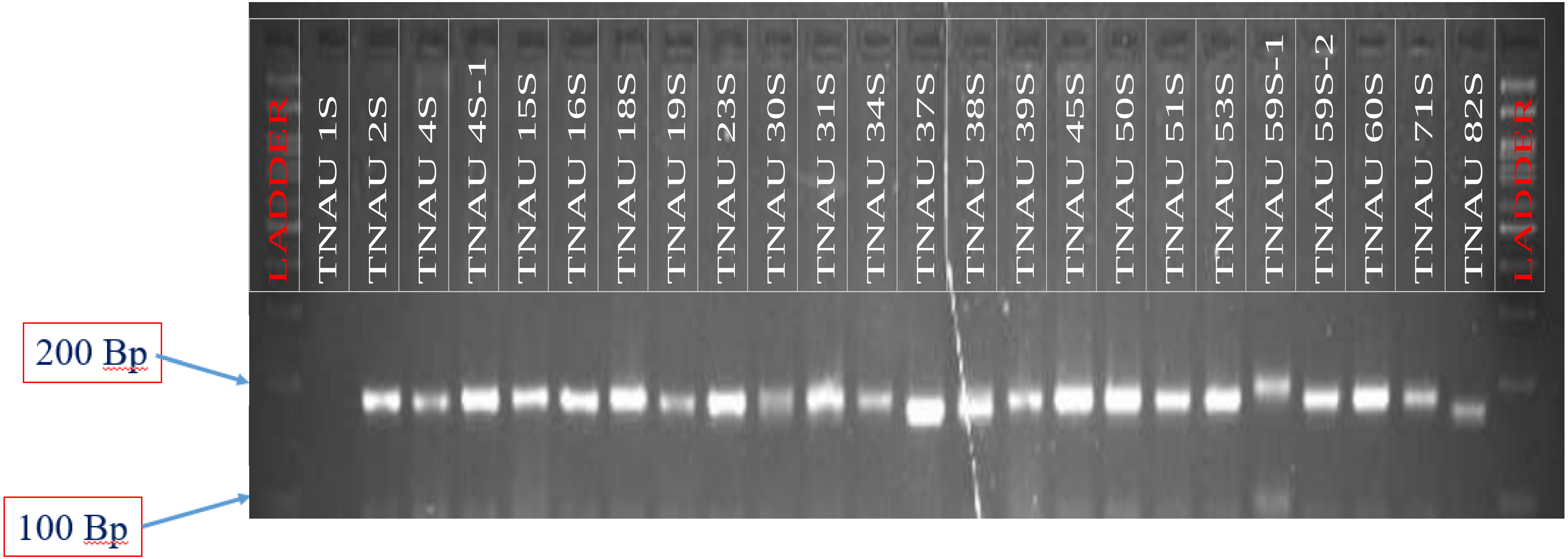

RM36
Chromosome-1

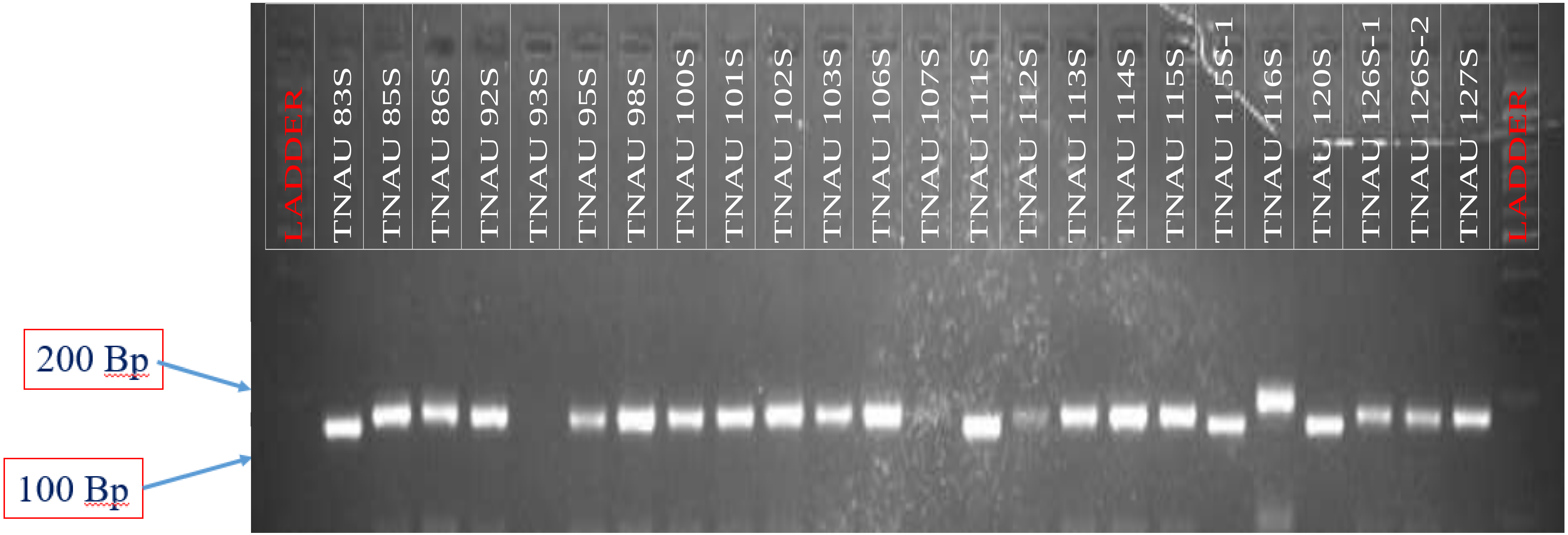

RM36
Chromosome-1

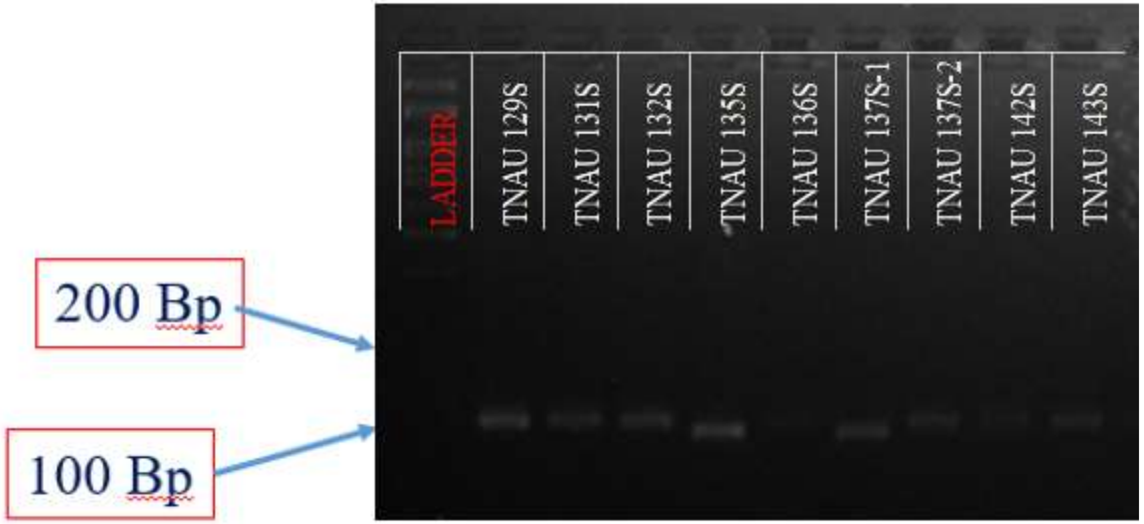

RM81
Chromosome-3

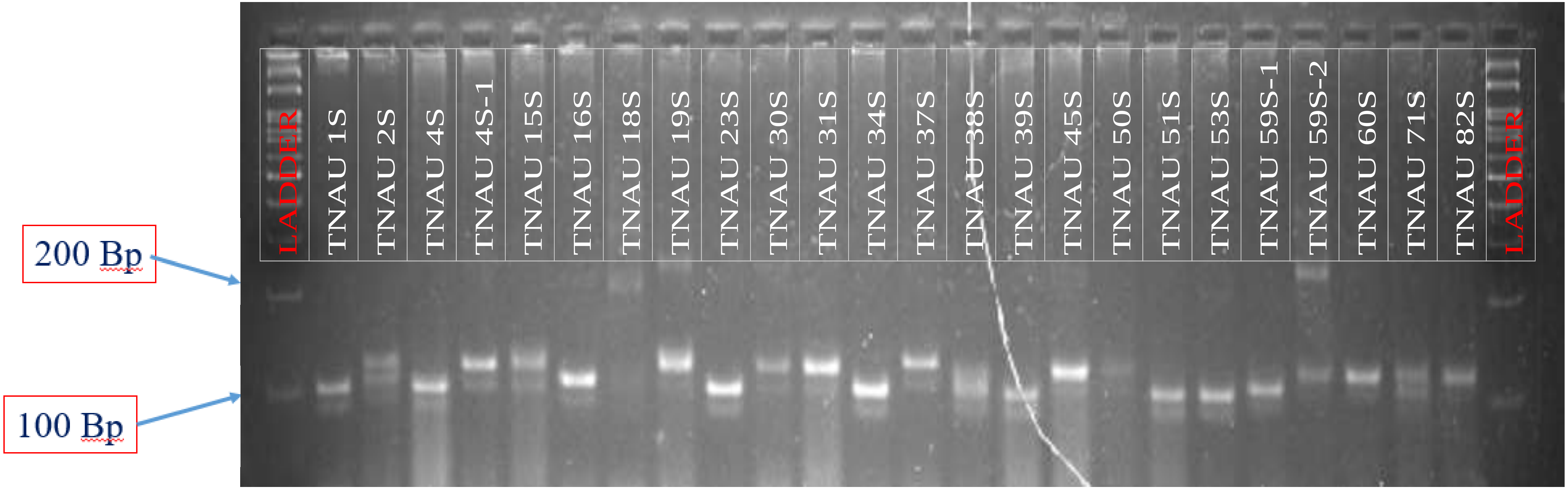

RM81
Chromosome-3

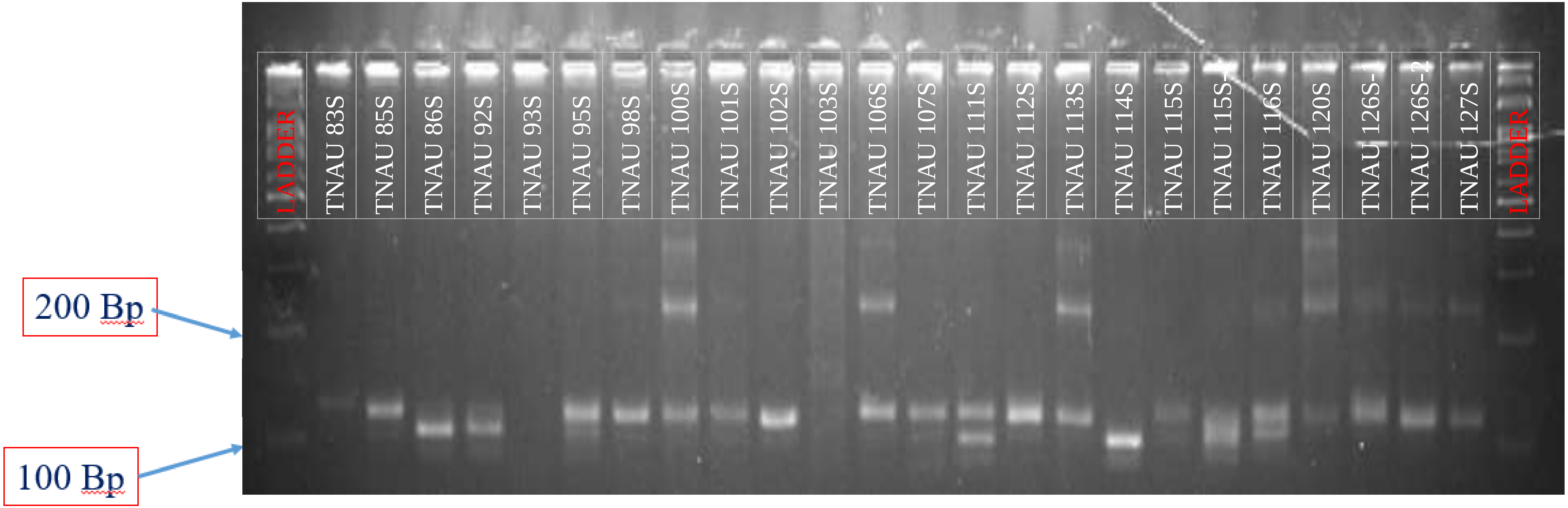

RM81
Chromosome-3

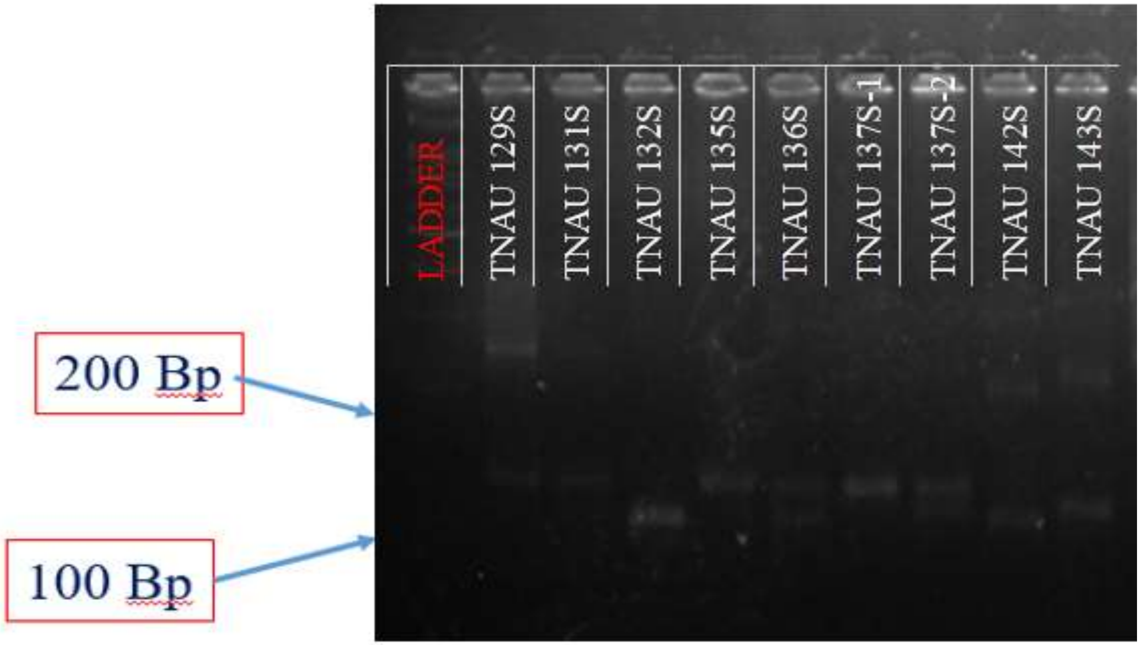

RM1018
Chromosome-4

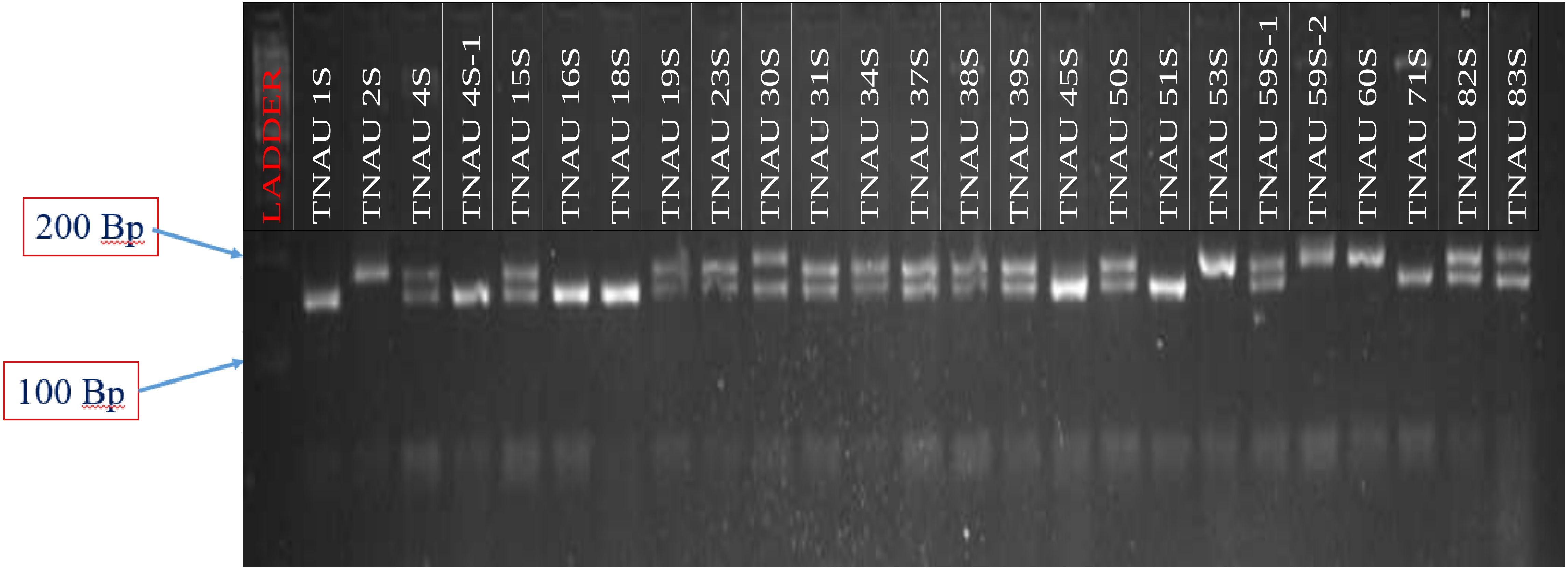

RM1018
Chromosome-4

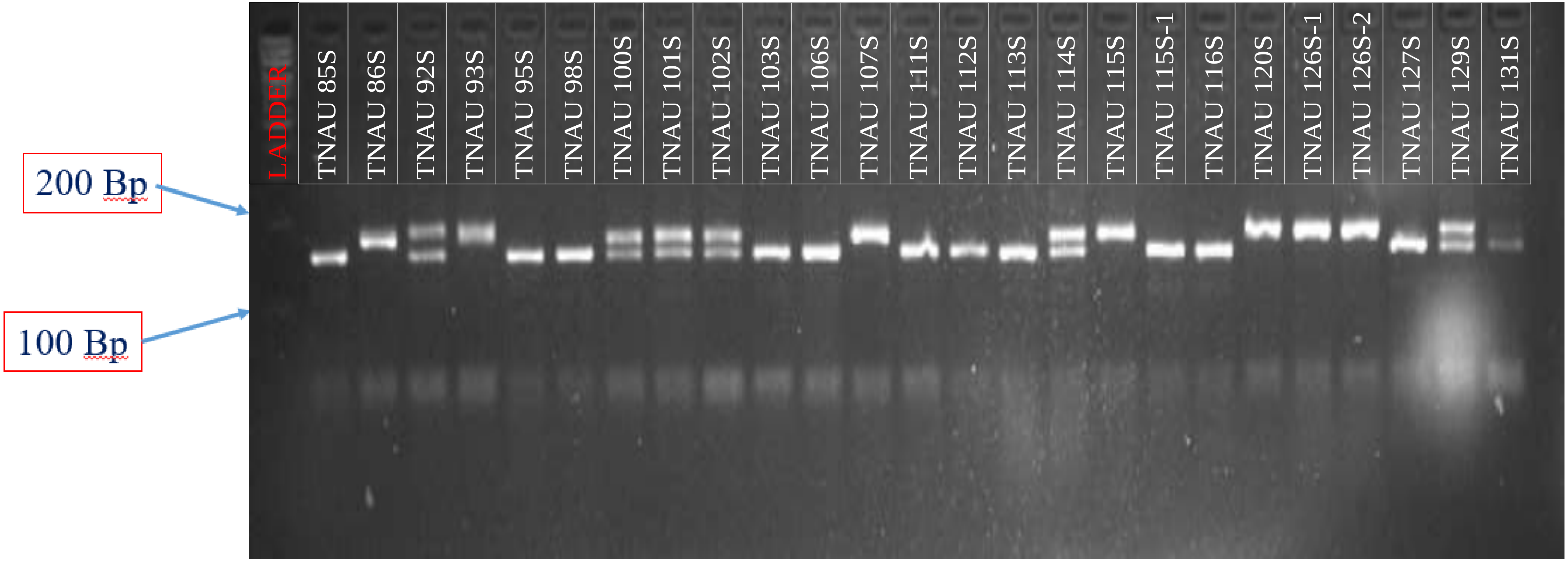

RM1018
Chromosome-4

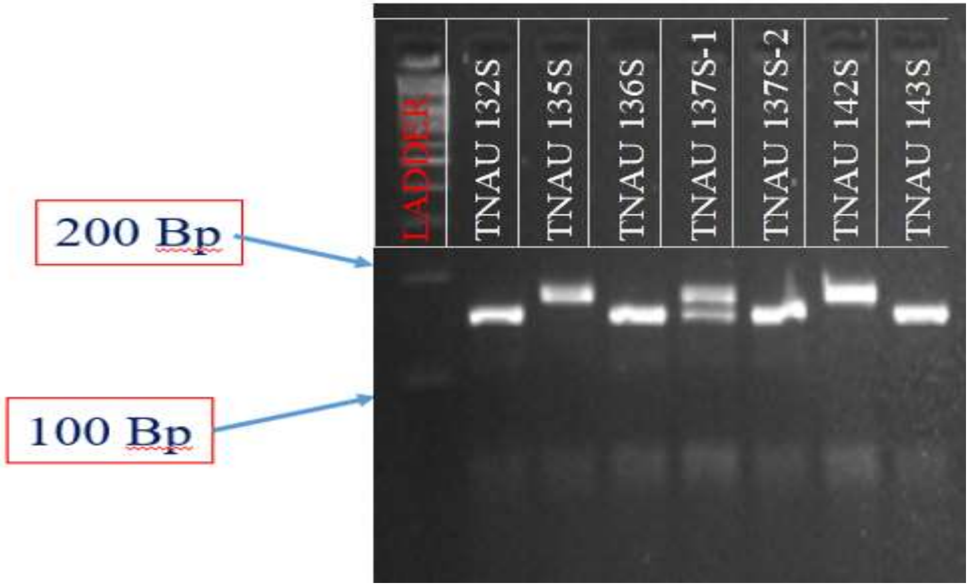

RM286
Chromosome-11

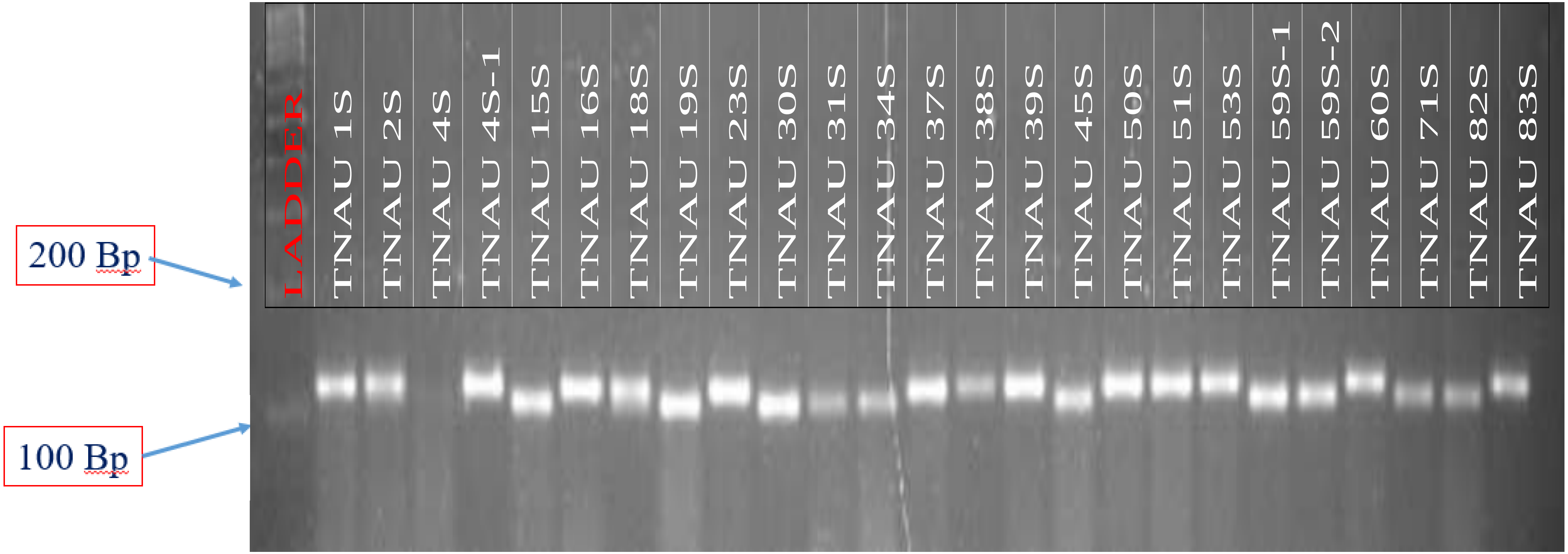

RM286
Chromosome-11

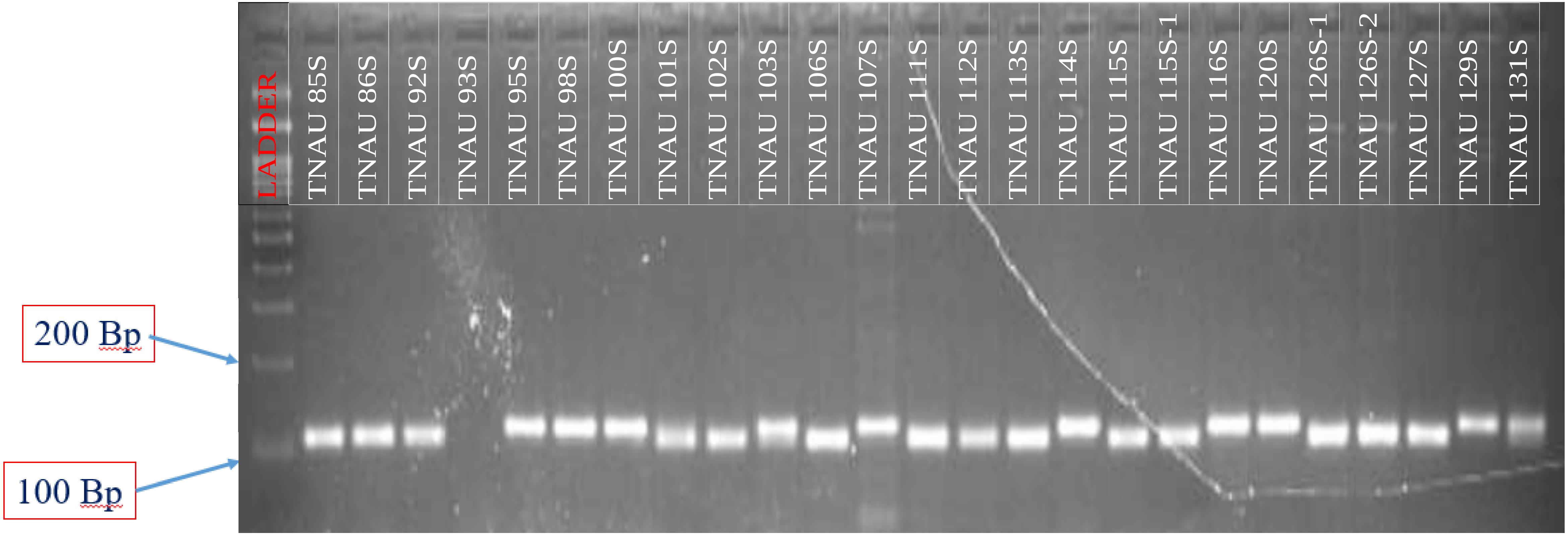

RM286
Chromosome-11

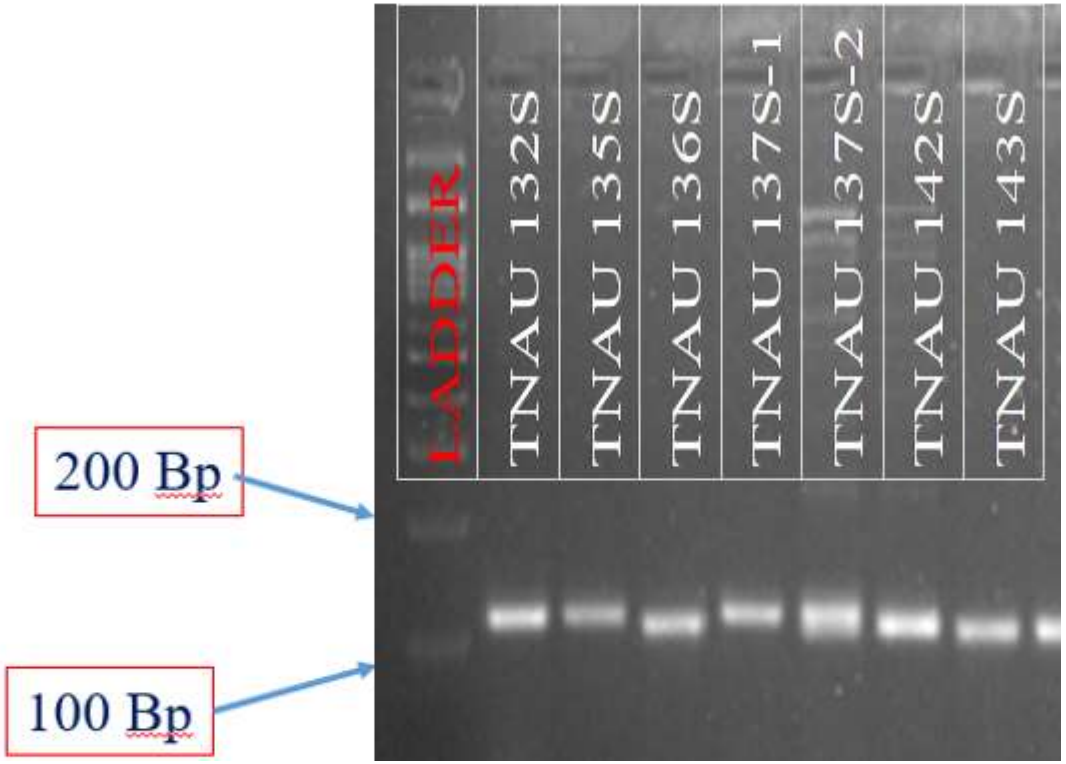

RM1896
Chromosome-9

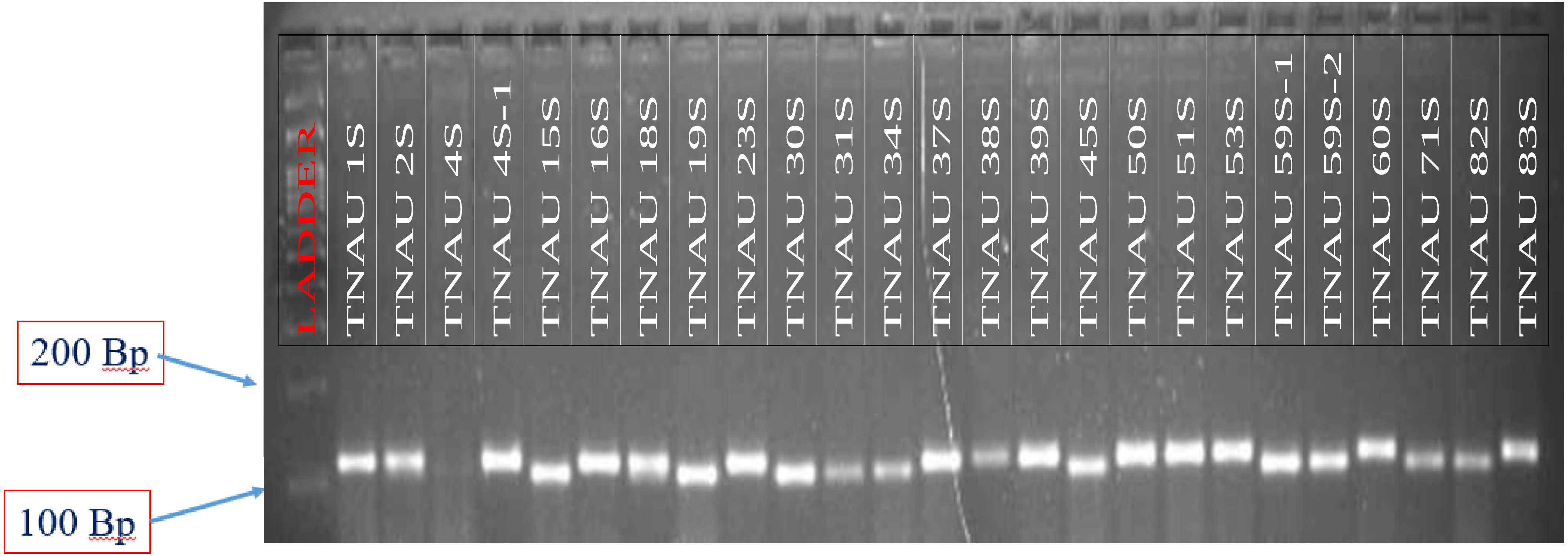

RM1896
Chromosome-9

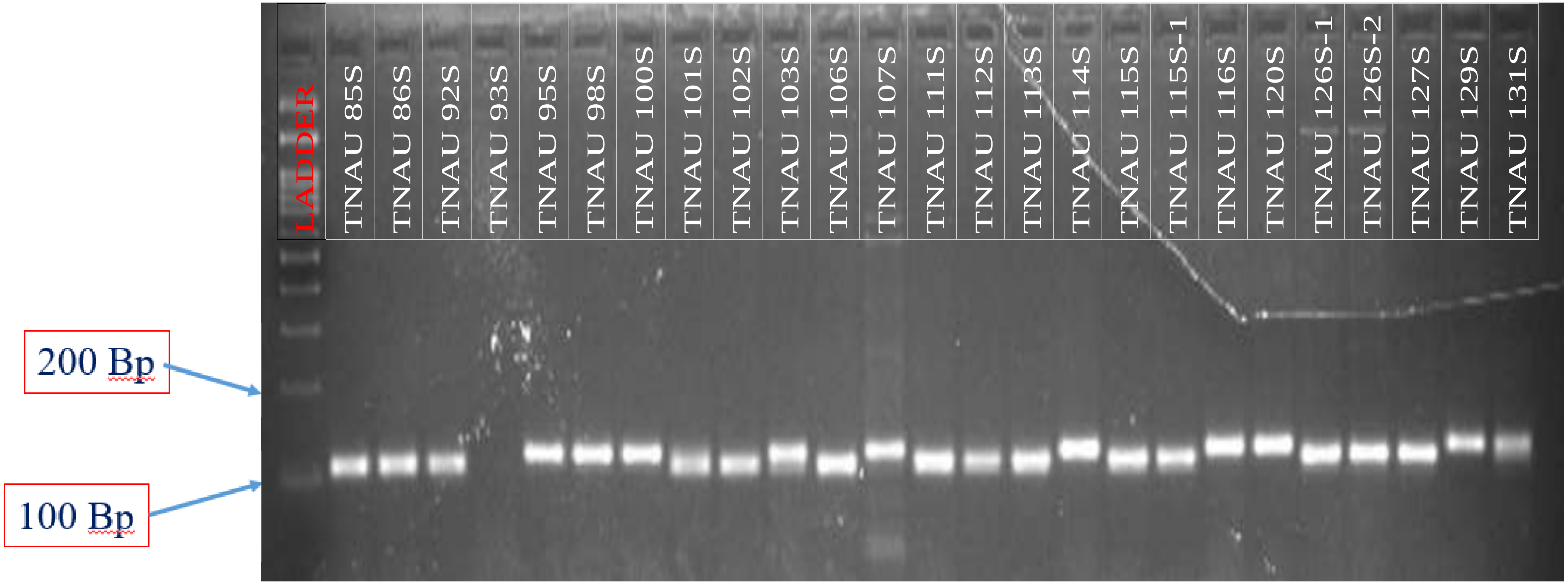

RM1896
Chromosome-9

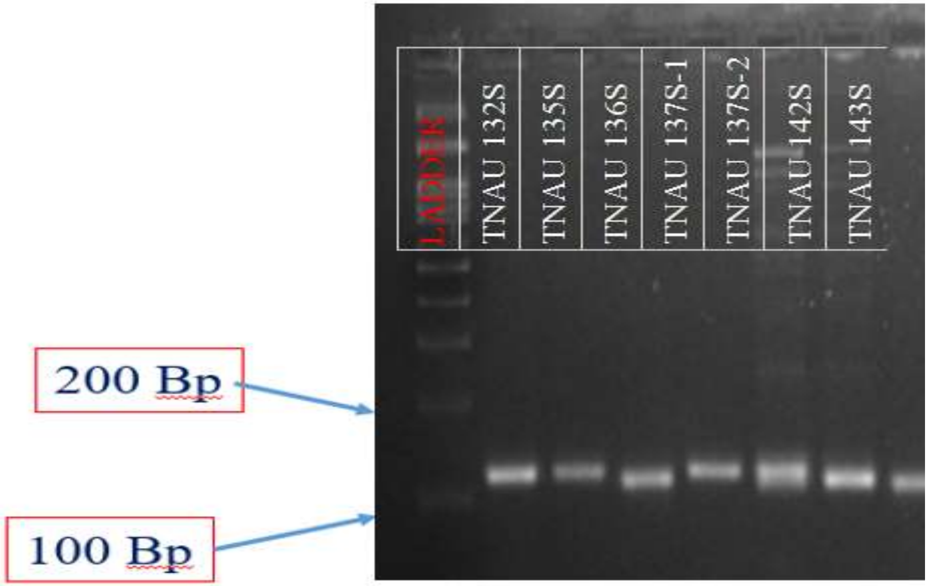

RM8134
Chromosome-1

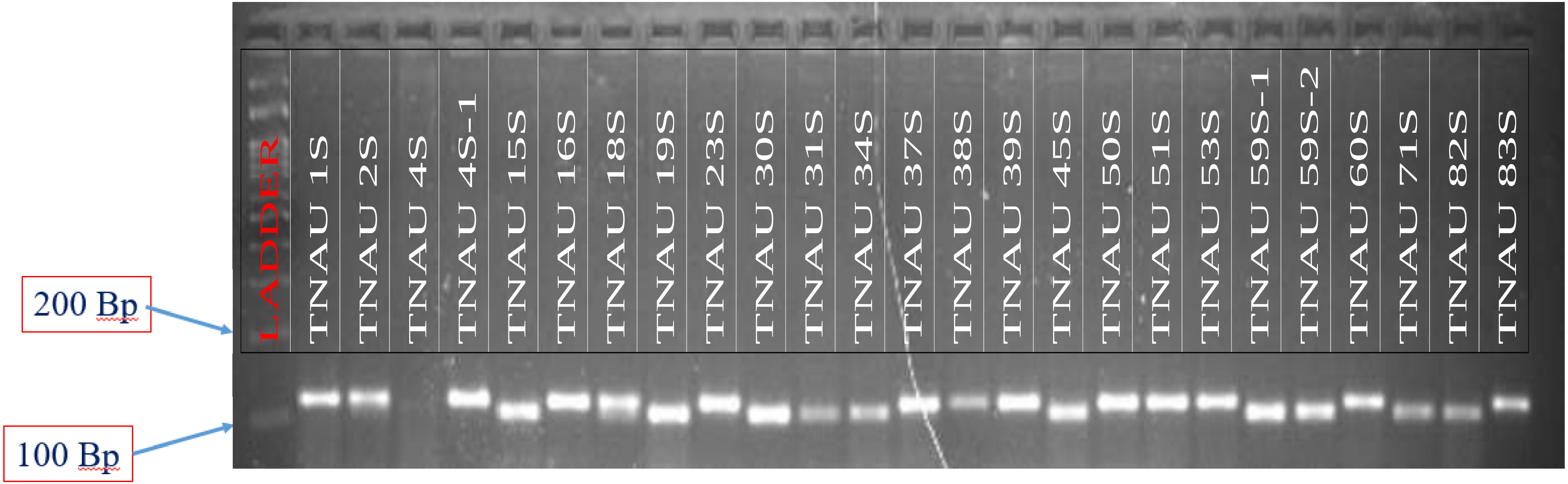

RM8134
Chromosome-1

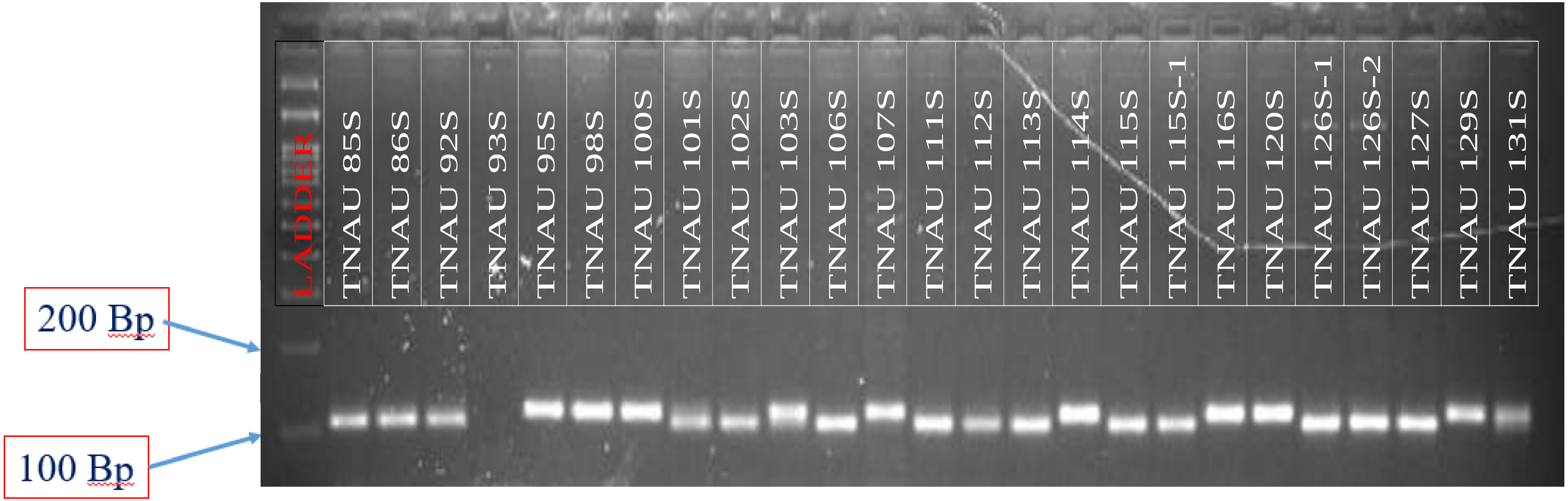

RM8134
Chromosome-1

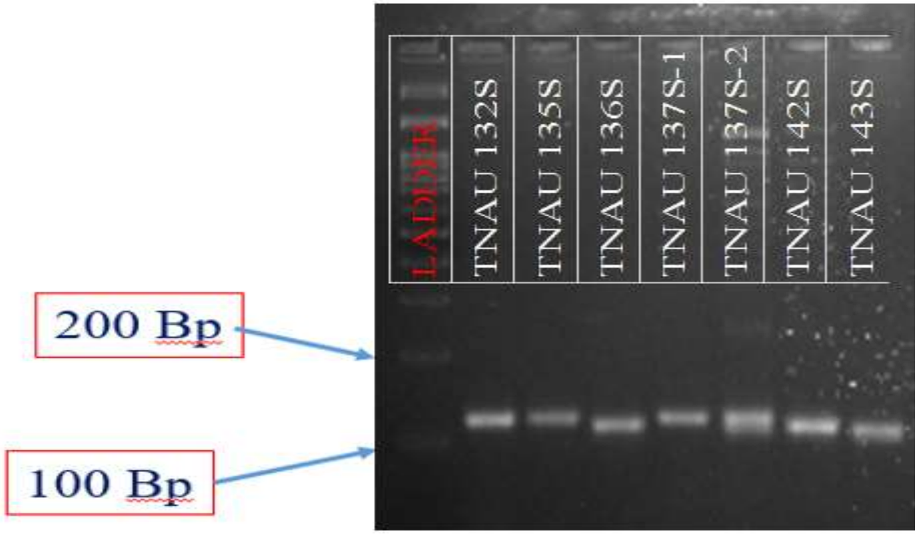

RM5709
Chromosome-4

200 Bp

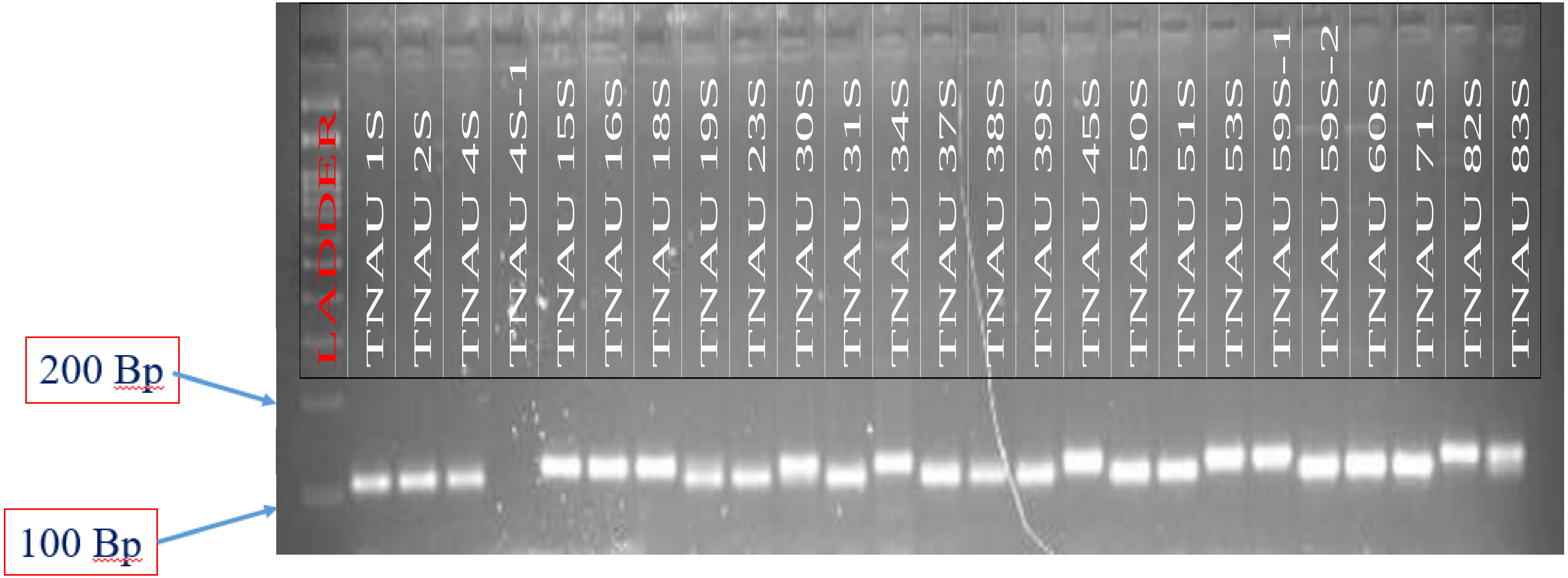

100 Bp

RM5709
Chromosome-4

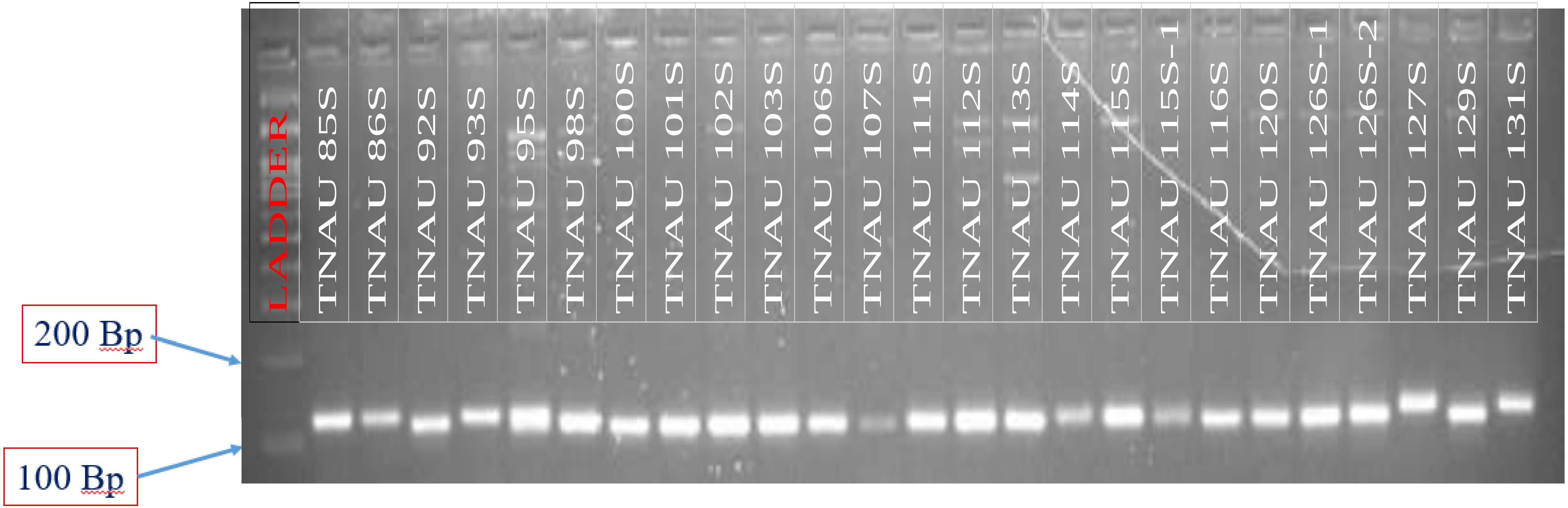

RM5709
Chromosome-4

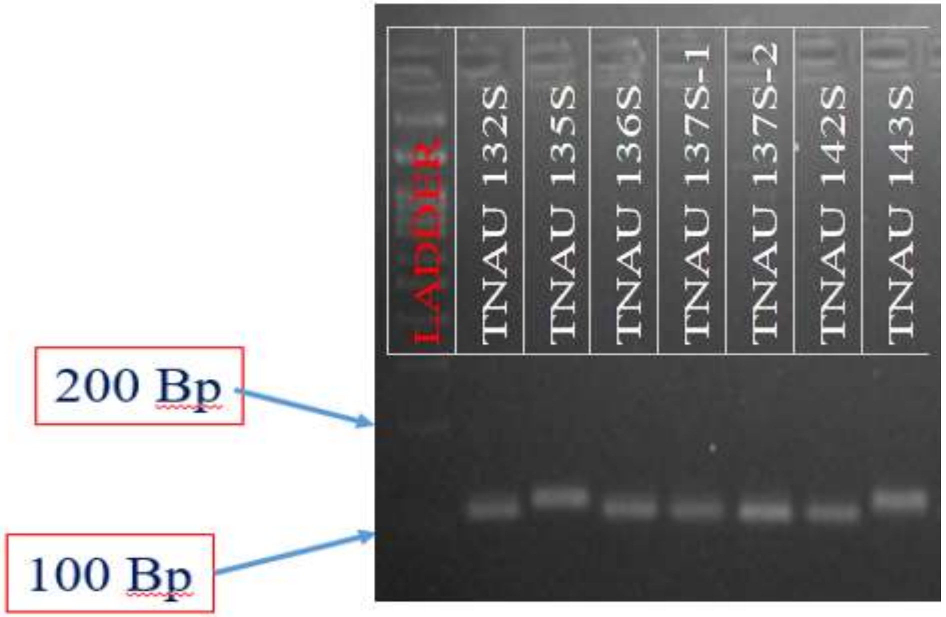

RM310
Chromosome-8

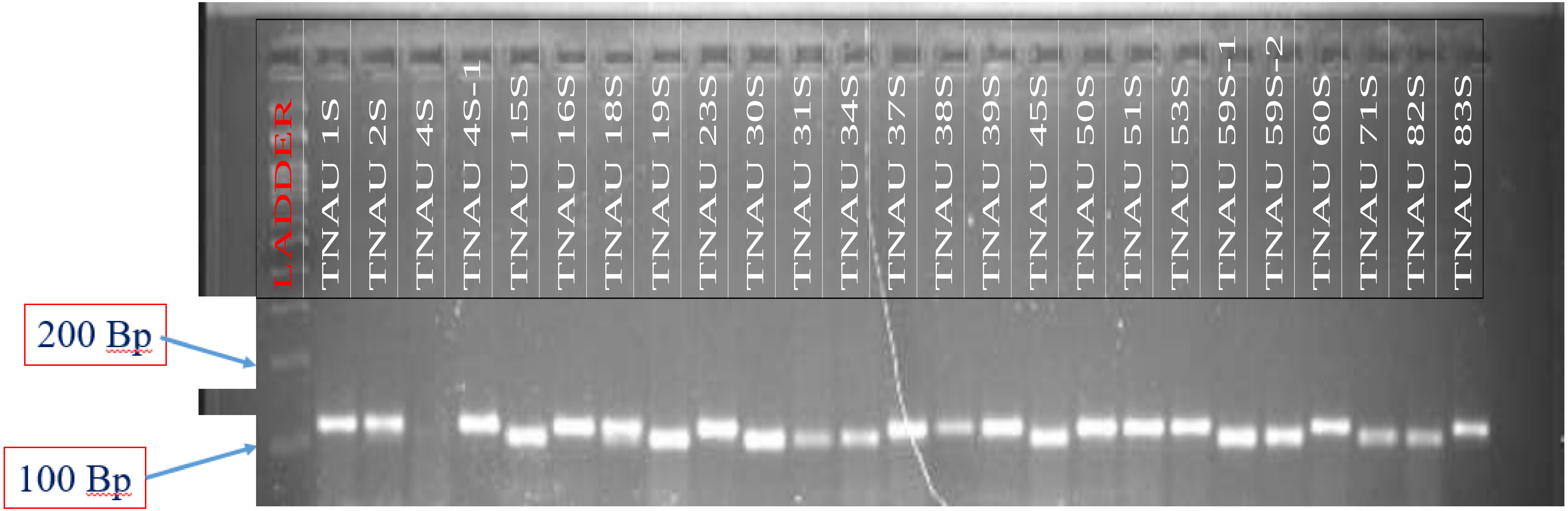

RM310
Chromosome-8

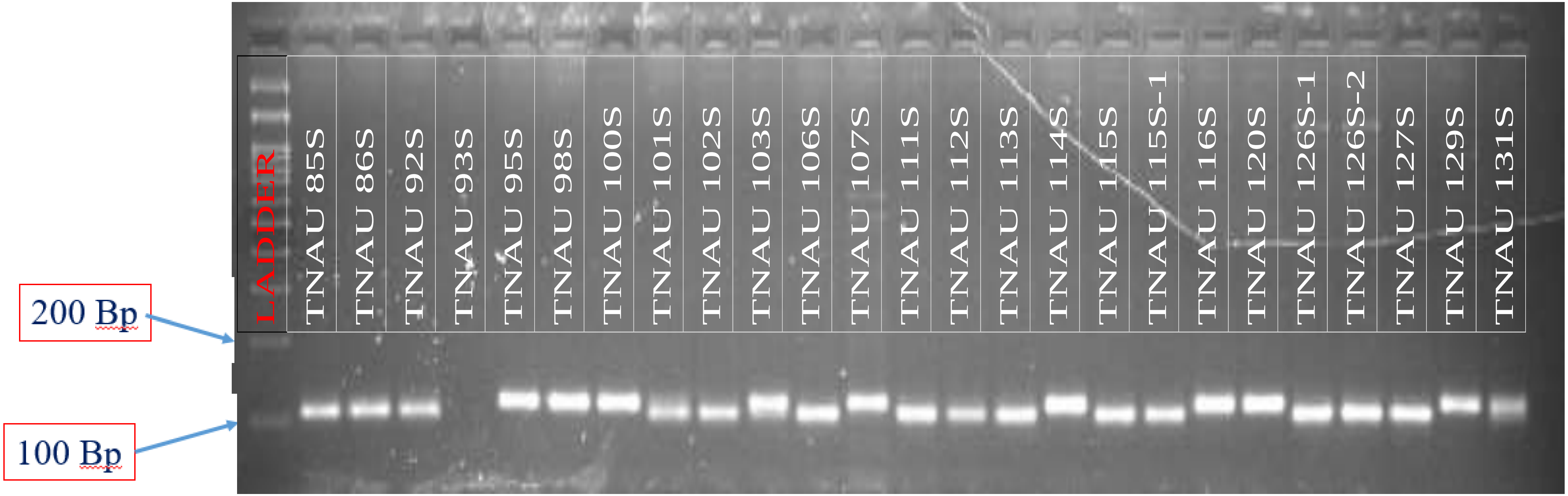

RM310
Chromosome-8

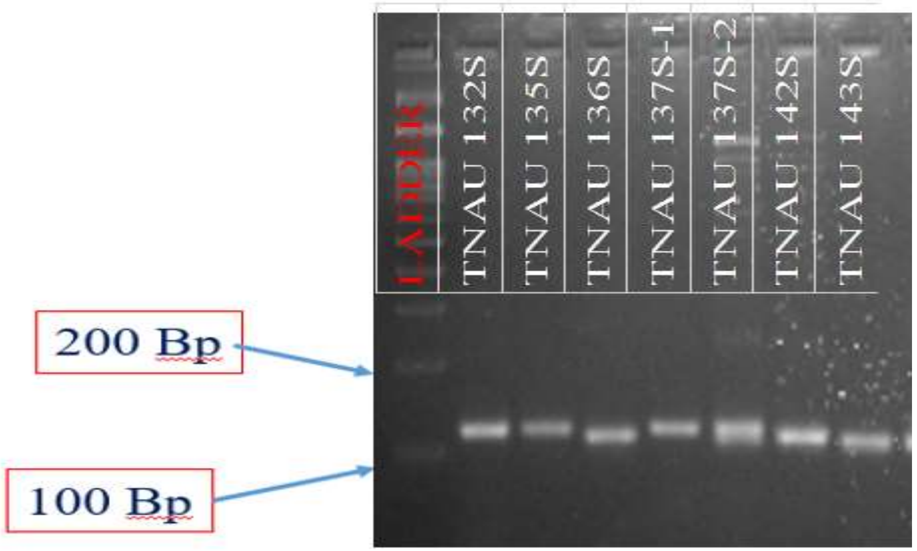

RM559
Chromosome-12

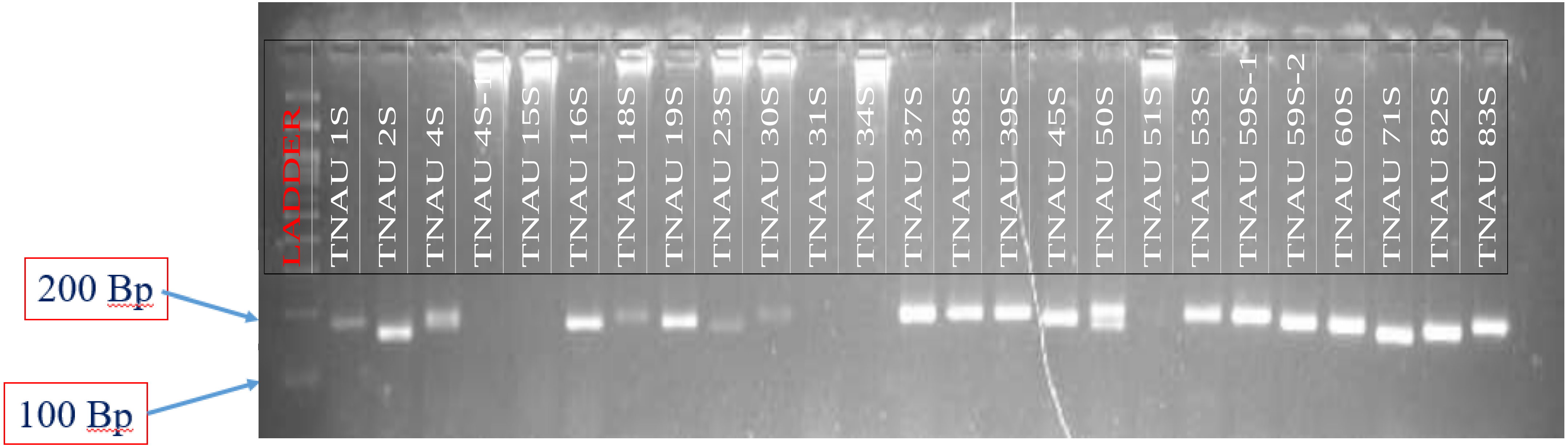

RM559
Chromosome-12

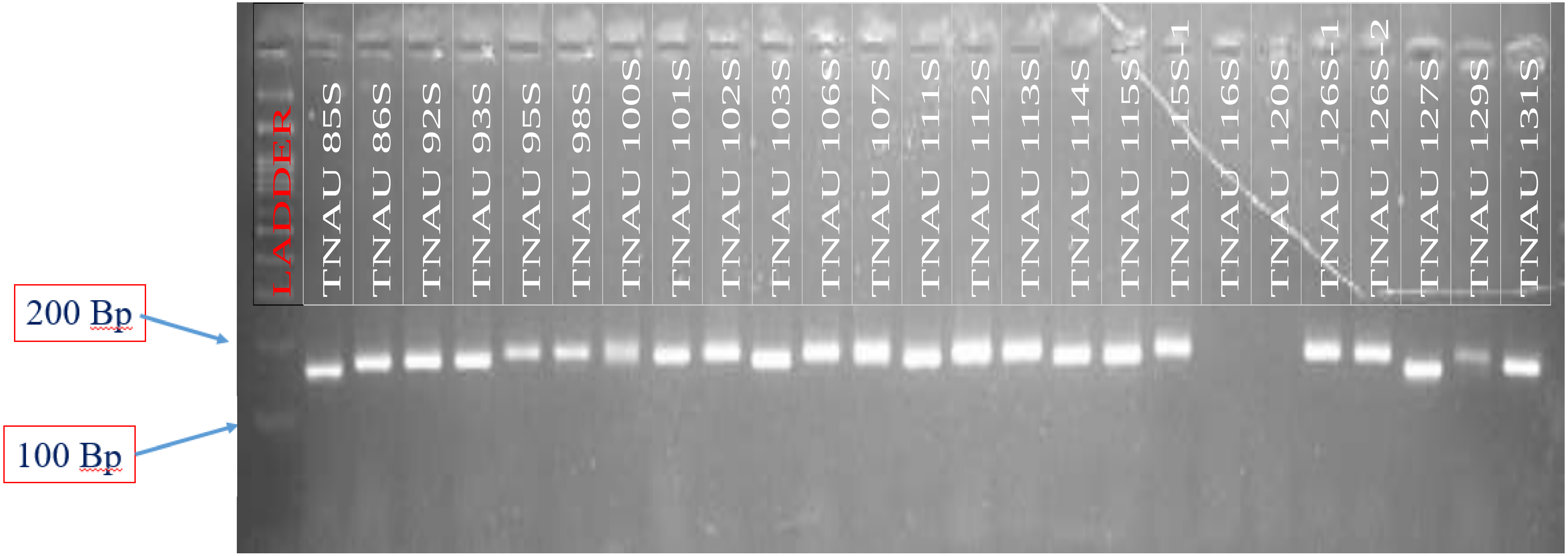

RM559
Chromosome-12

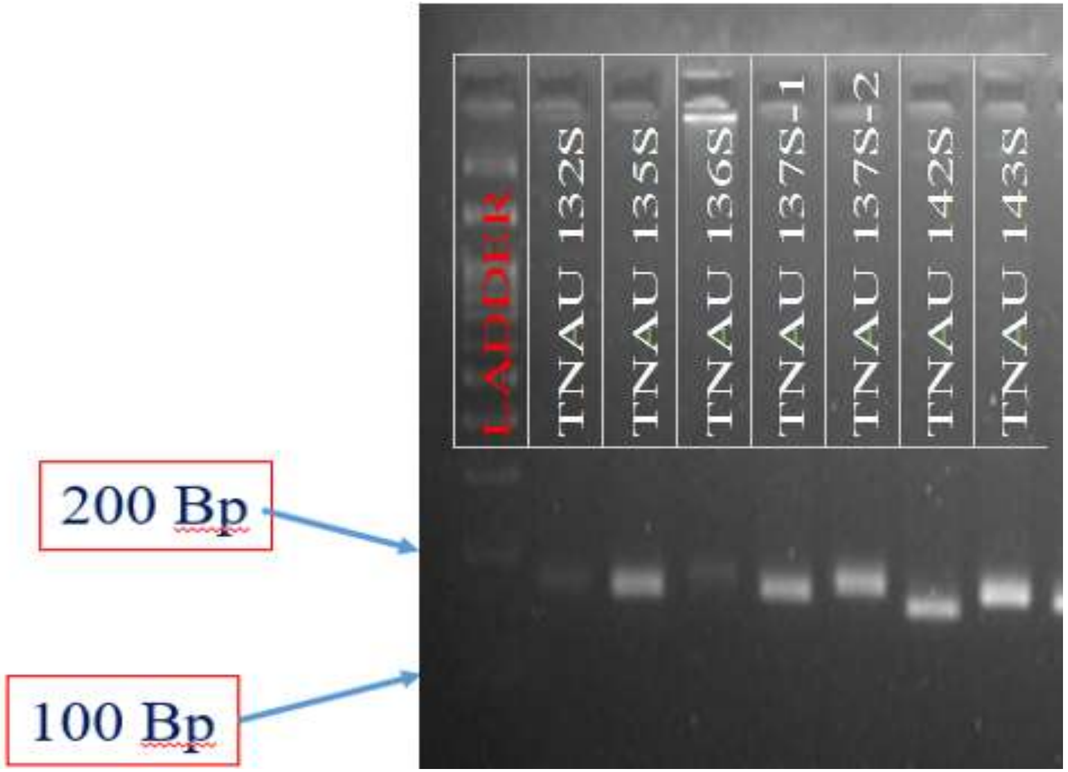

RM16559
Chromosome-4

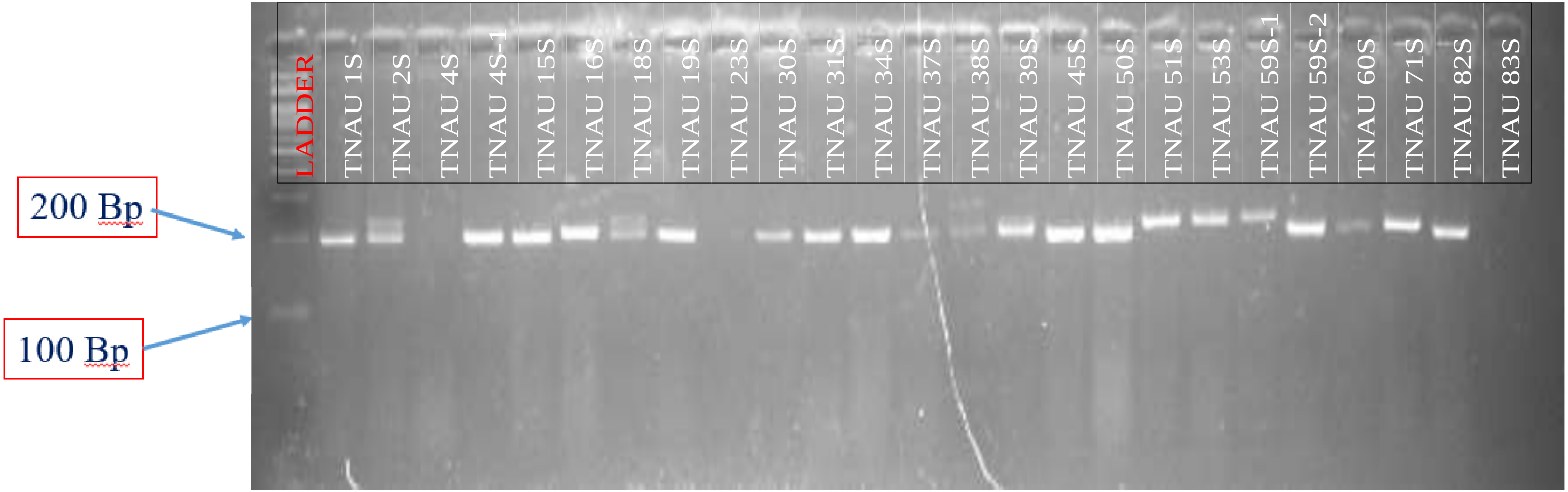

RM16559
Chromosome-4

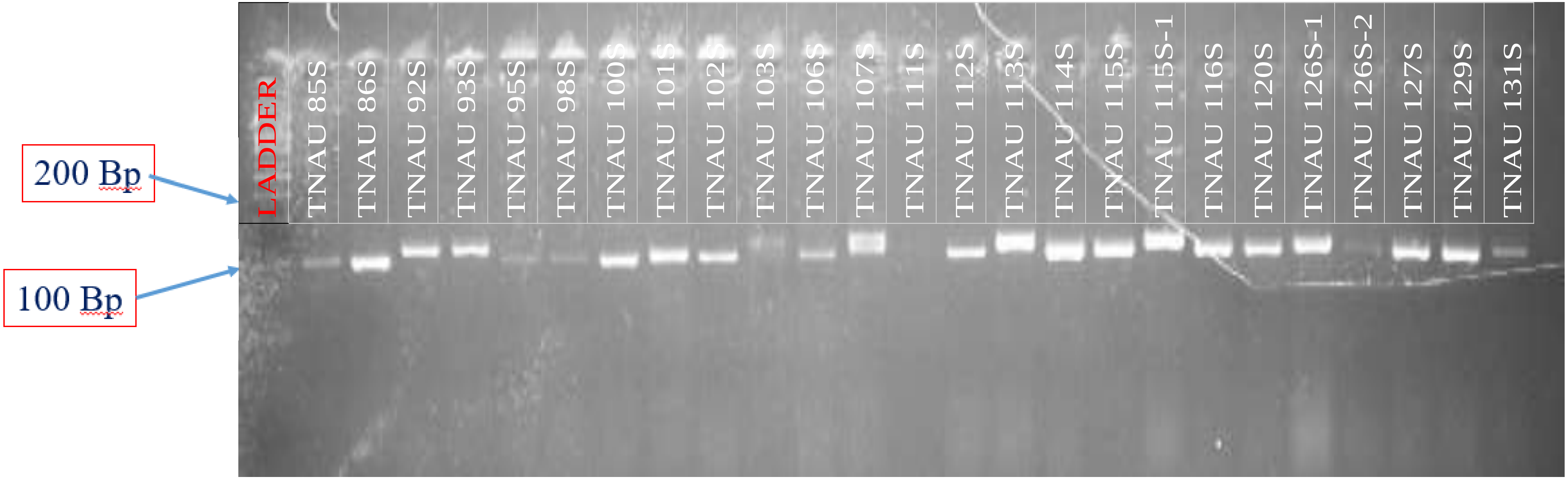

RM16559
Chromosome-4

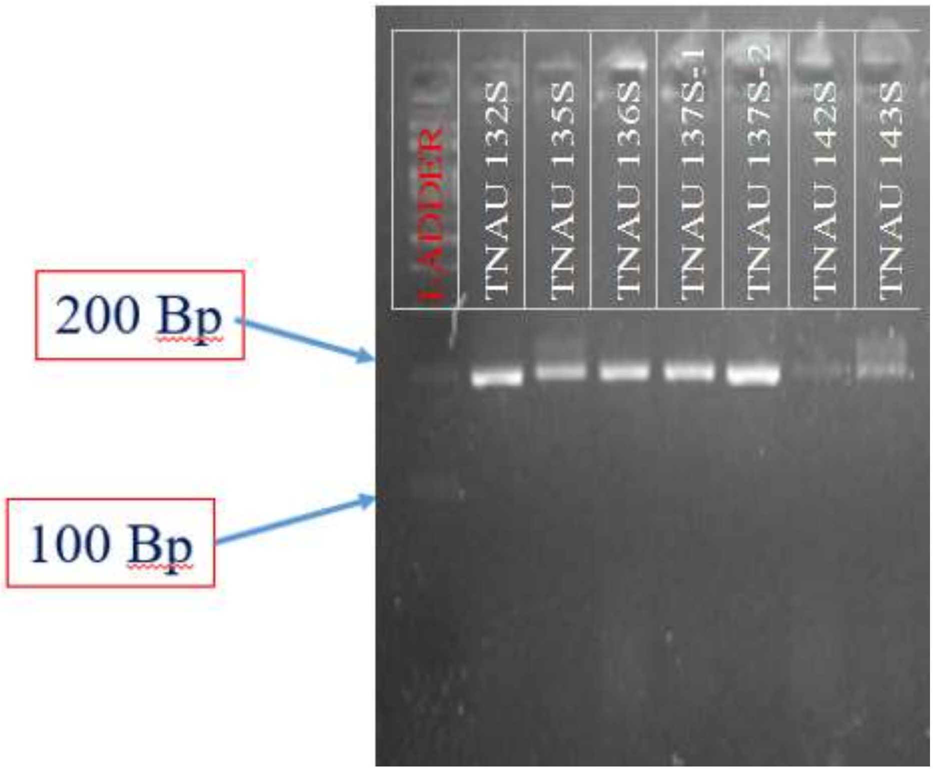

RM420
Chromosome-7

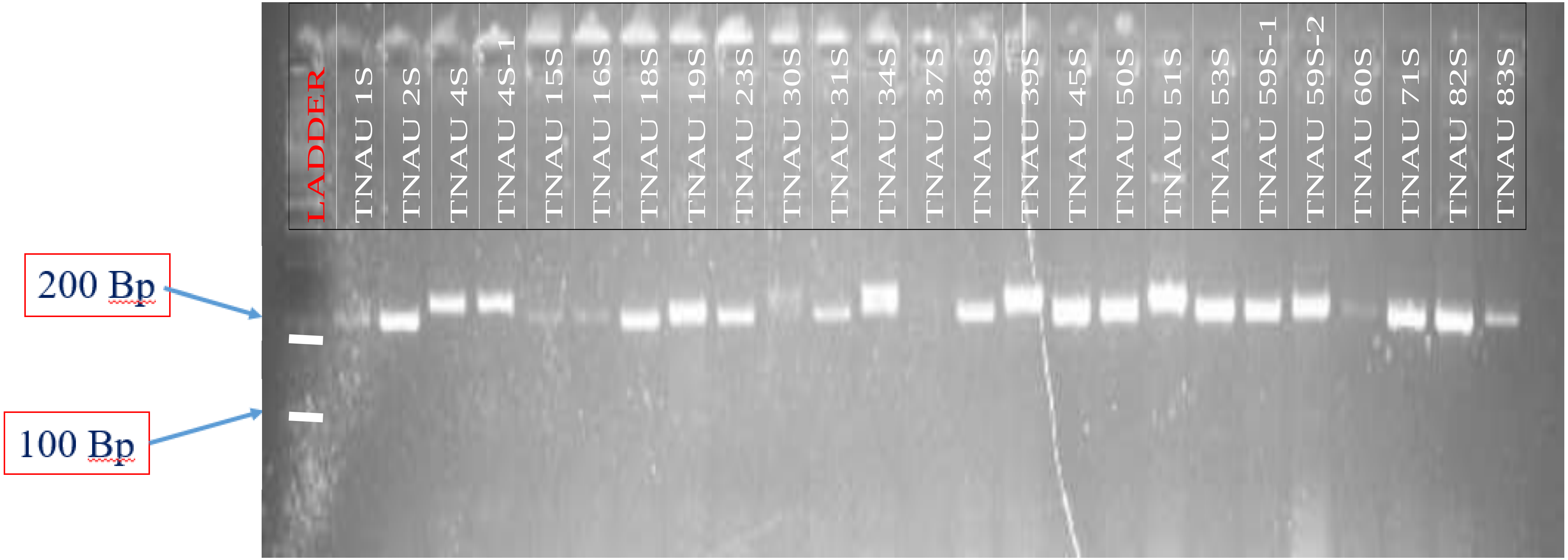

RM420
Chromosome-7

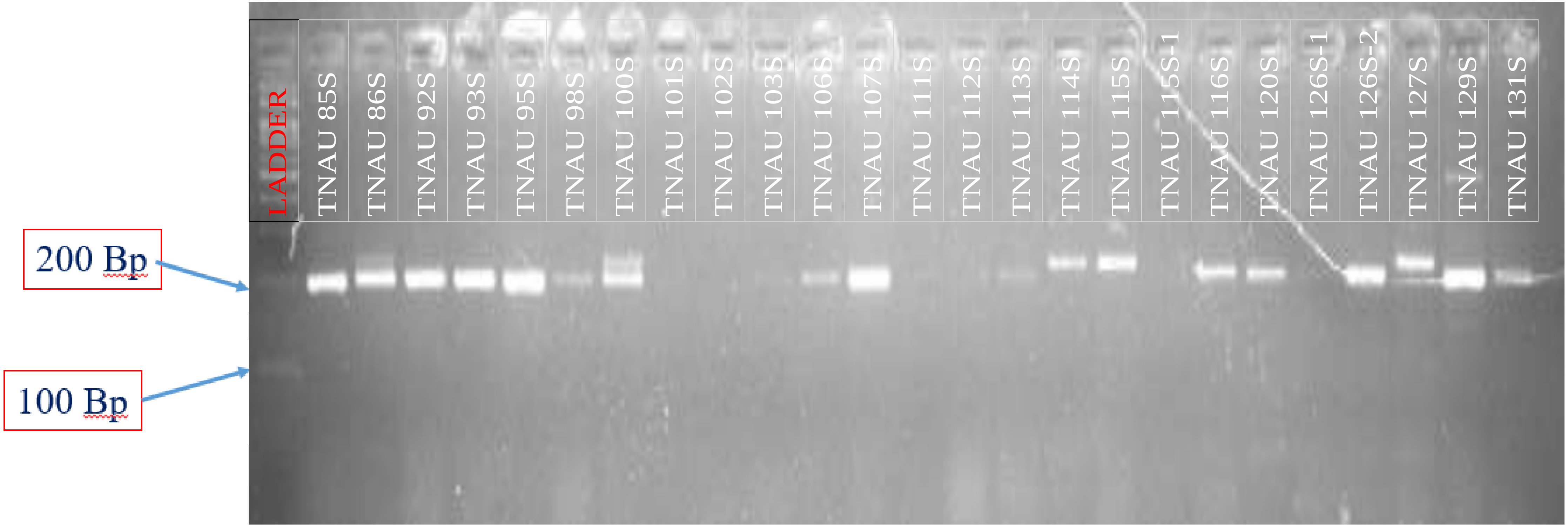

RM420
Chromosome-7

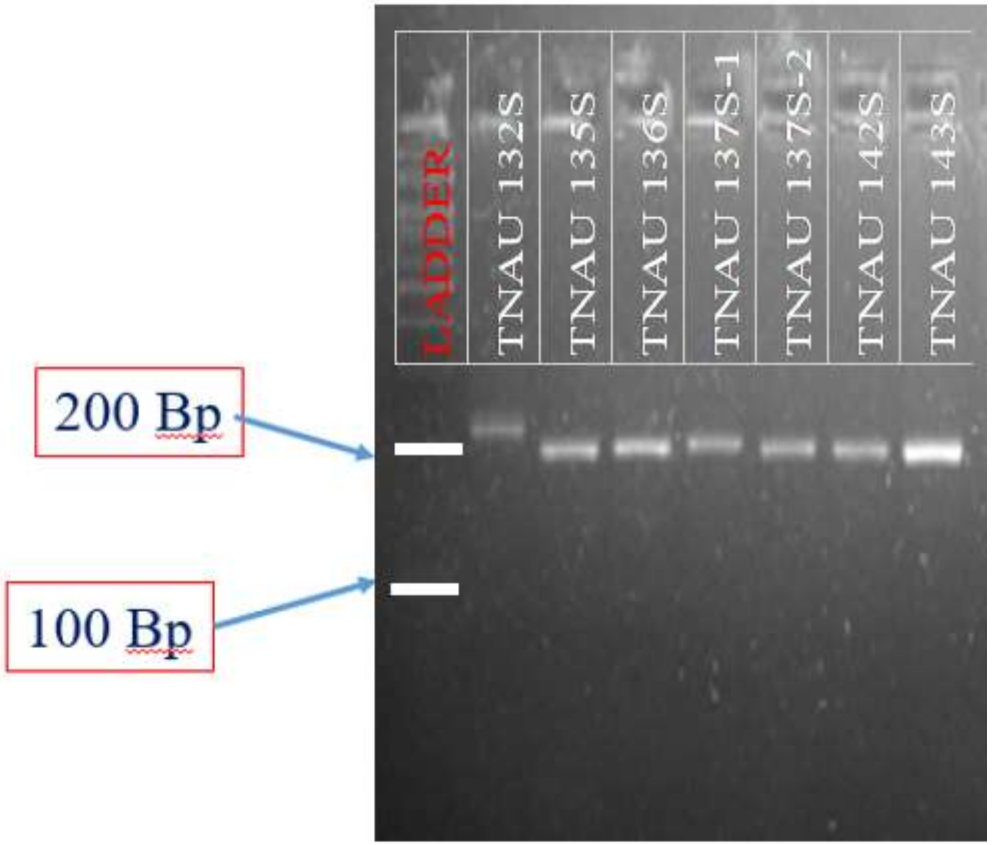

RM282
Chromosome-5

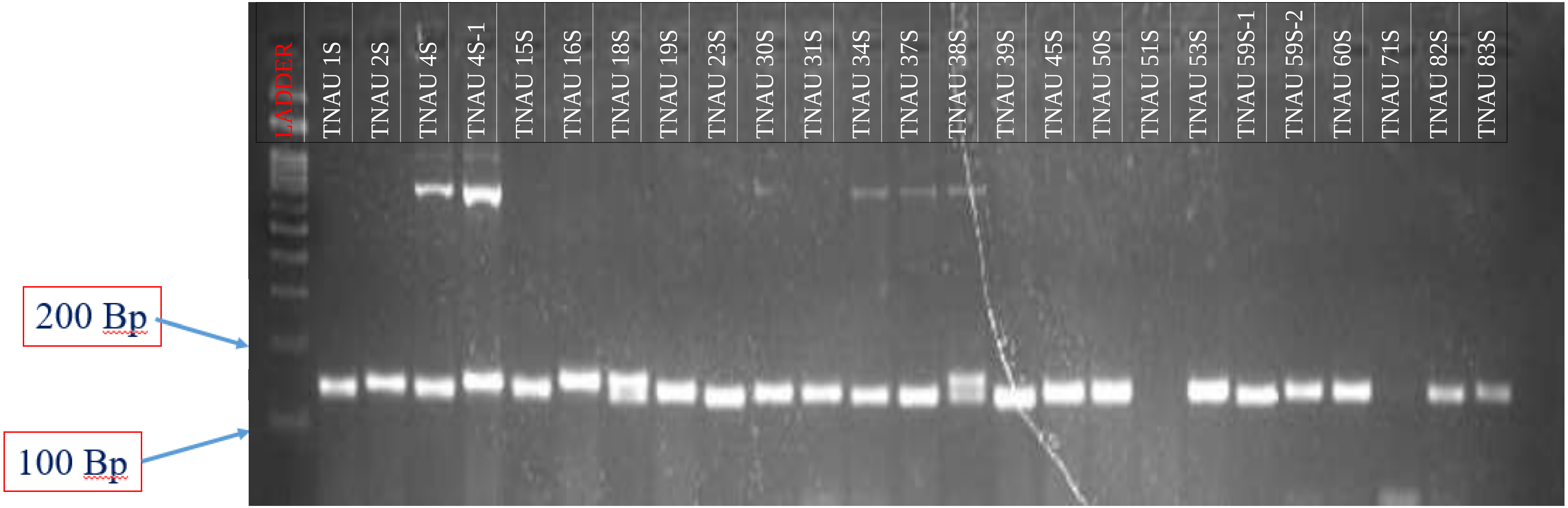

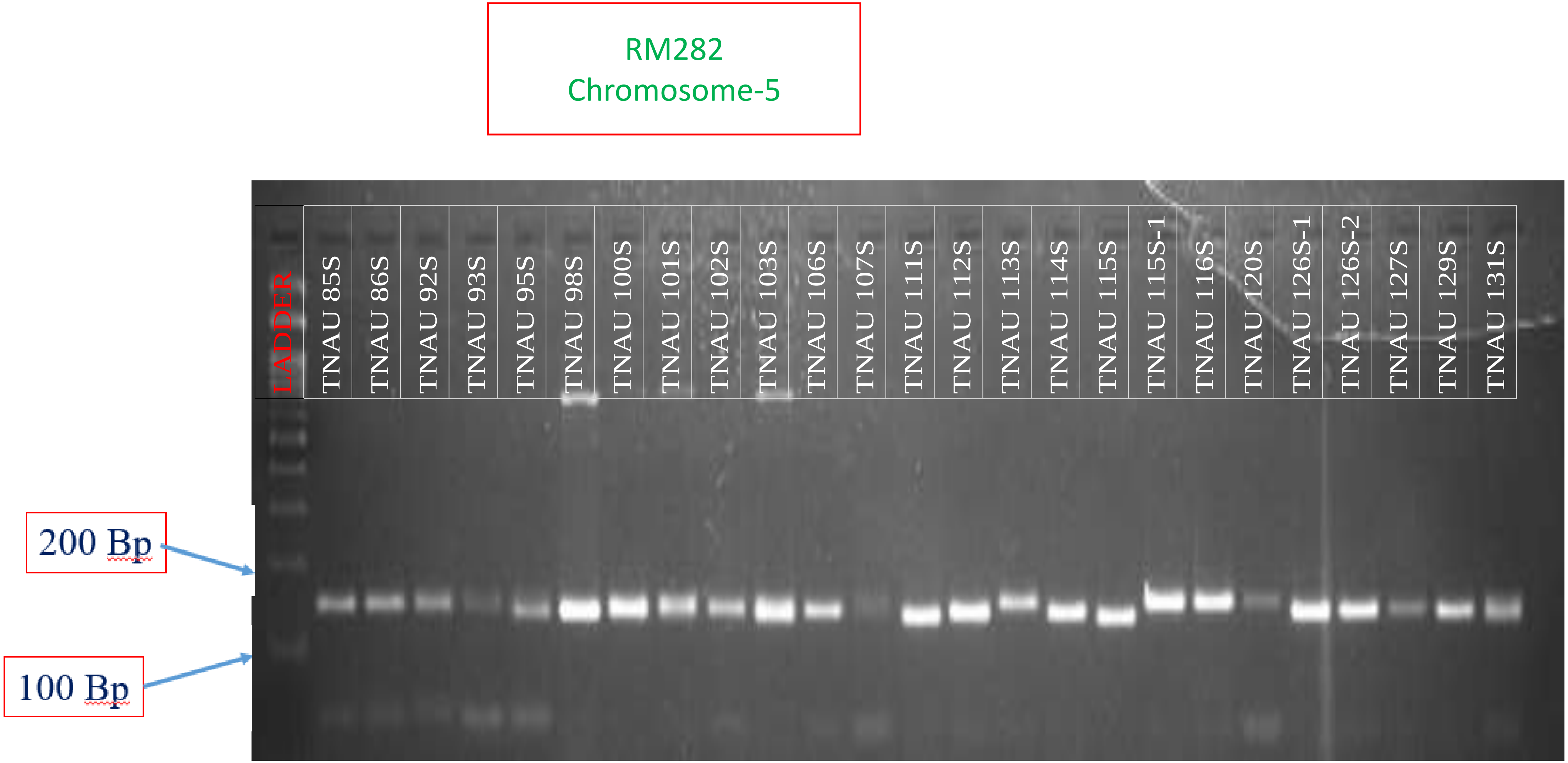
RM282
Chromosome-5
200 Bp
100 Bp
LADDER
TNAU 85S
TNAU 86S
TNAU 92S
TNAU 93S
TNAU 95S
TNAU 98S
TNAU 100S
TNAU 101S
TNAU 102S
TNAU 103S
TNAU 106S
TNAU 107S
TNAU 111S
TNAU 112S
TNAU 113S
TNAU 114S
TNAU 115S
TNAU 115S-1
TNAU 116S
TNAU 120S
TNAU 126S-1
TNAU 126S-2
TNAU 127S
TNAU 129S
TNAU 131S

RM282
Chromosome-5

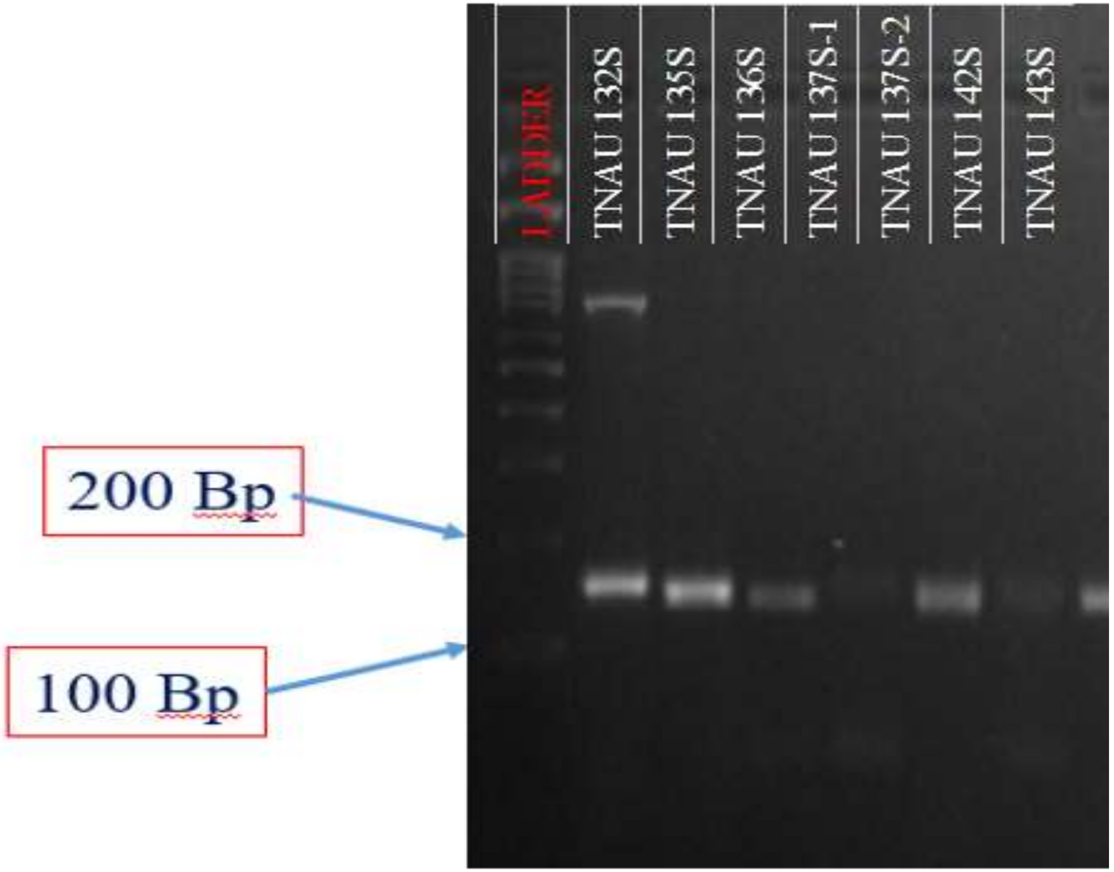

RM210
Chromosome-8

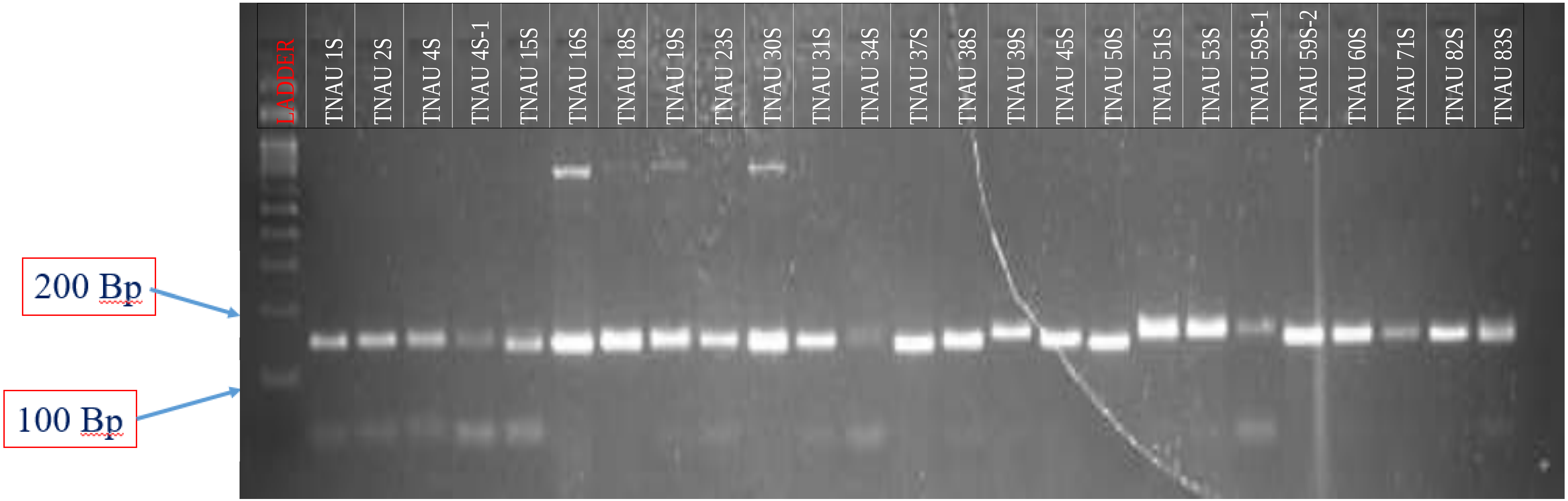

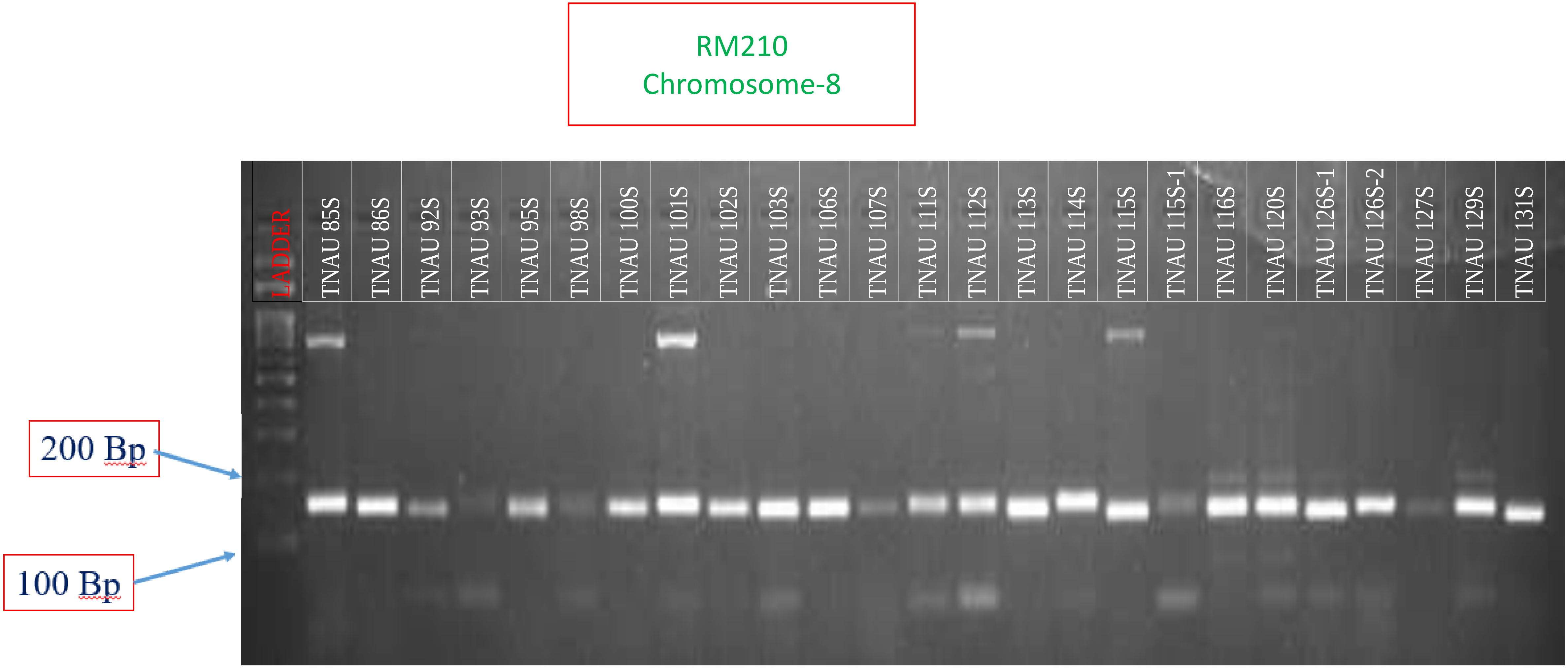
RM210
Chromosome-8
200 Bp
100 Bp
LADDER
TNAU 85S
TNAU 86S
TNAU 92S
TNAU 93S
TNAU 95S
TNAU 98S
TNAU 100S
TNAU 101S
TNAU 102S
TNAU 103S
TNAU 106S
TNAU 107S
TNAU 111S
TNAU 112S
TNAU 113S
TNAU 114S
TNAU 115S
TNAU 115S-1
TNAU 116S
TNAU 120S
TNAU 126S-1
TNAU 126S-2
TNAU 127S
TNAU 129S
TNAU 131S

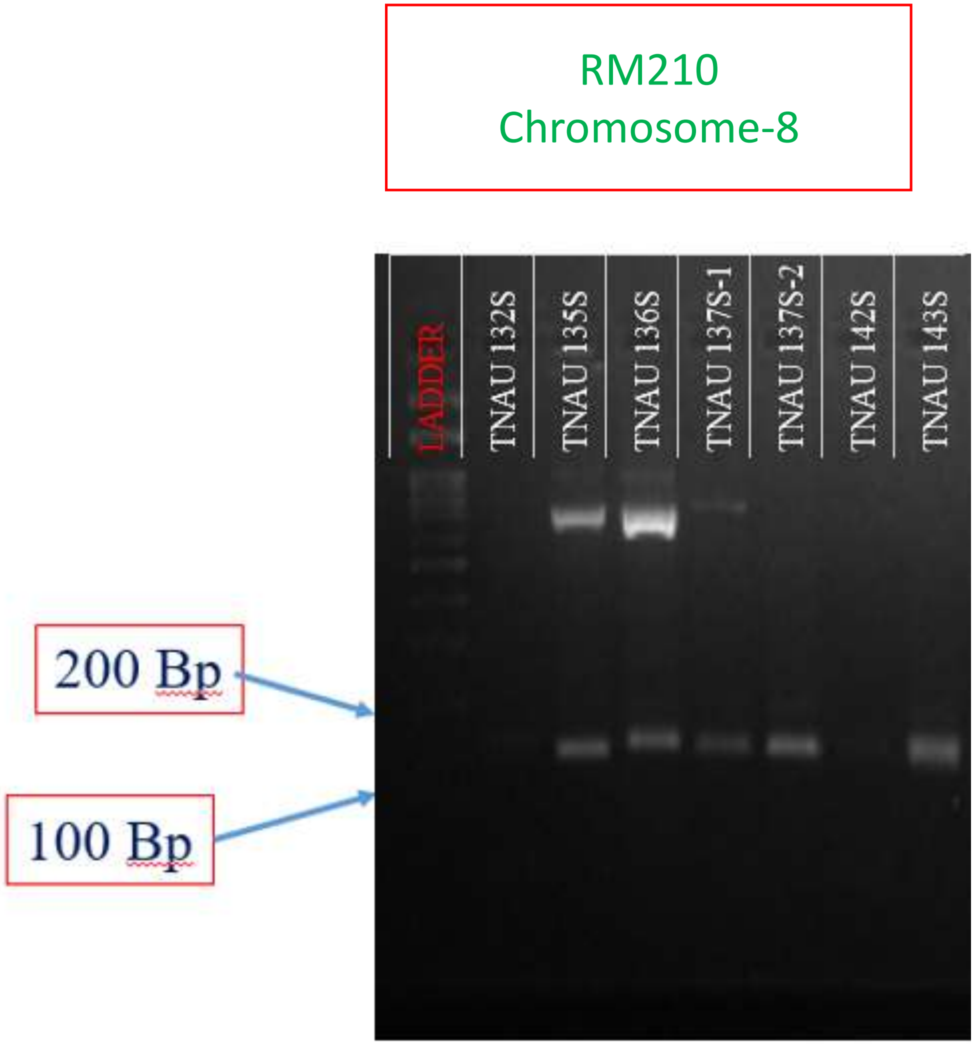
RM210
Chromosome-8
LADDER
TNAU 132S
TNAU 135S
TNAU 136S
TNAU 137S-1
TNAU 137S-2
TNAU 142S
TNAU 143S
200 Bp
100 Bp

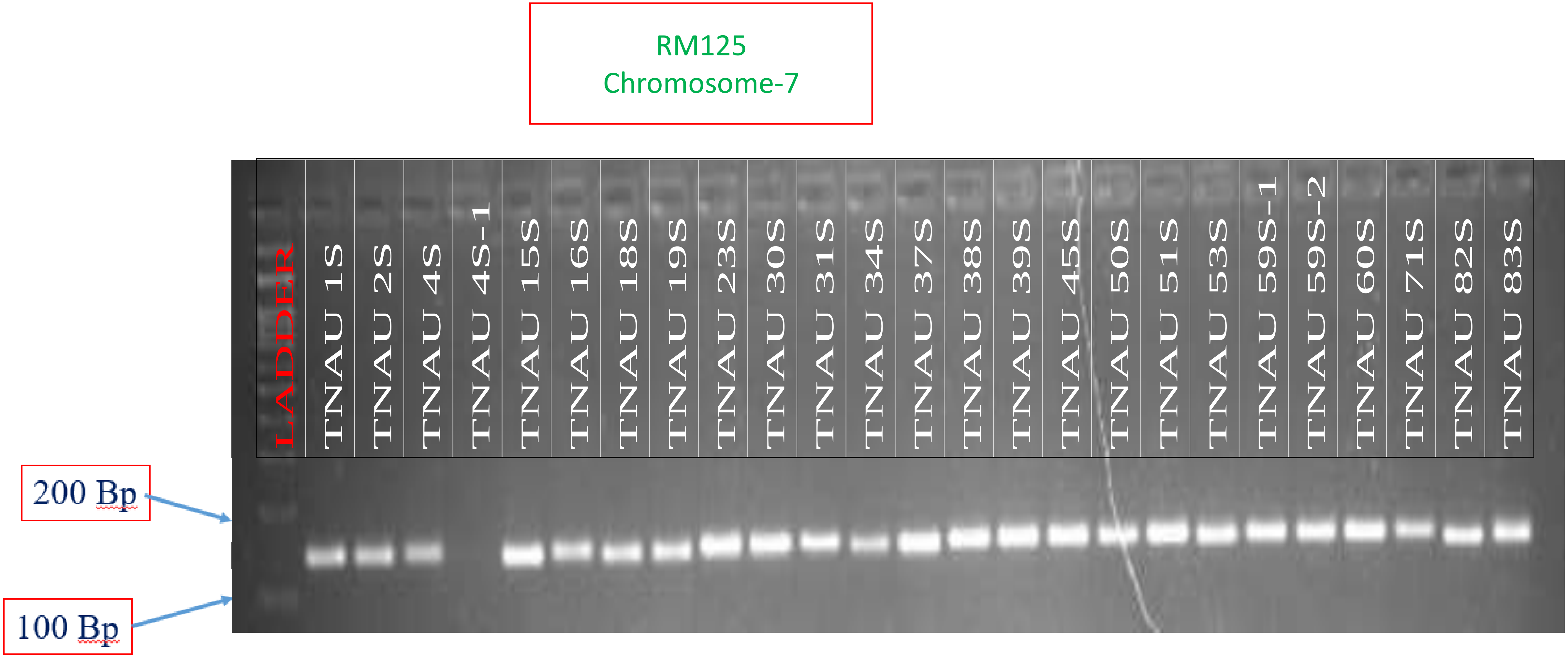
RM125
Chromosome-7
LADDER
TNAU 1S
TNAU 2S
TNAU 4S
TNAU 4S-1
TNAU 15S
TNAU 16S
TNAU 18S
TNAU 19S
TNAU 23S
TNAU 30S
TNAU 31S
TNAU 34S
TNAU 37S
TNAU 38S
TNAU 39S
TNAU 45S
TNAU 50S
TNAU 51S
TNAU 53S
TNAU 59S-1
TNAU 59S-2
TNAU 60S
TNAU 71S
TNAU 82S
TNAU 83S
200 Bp
100 Bp

RM125
Chromosome-7

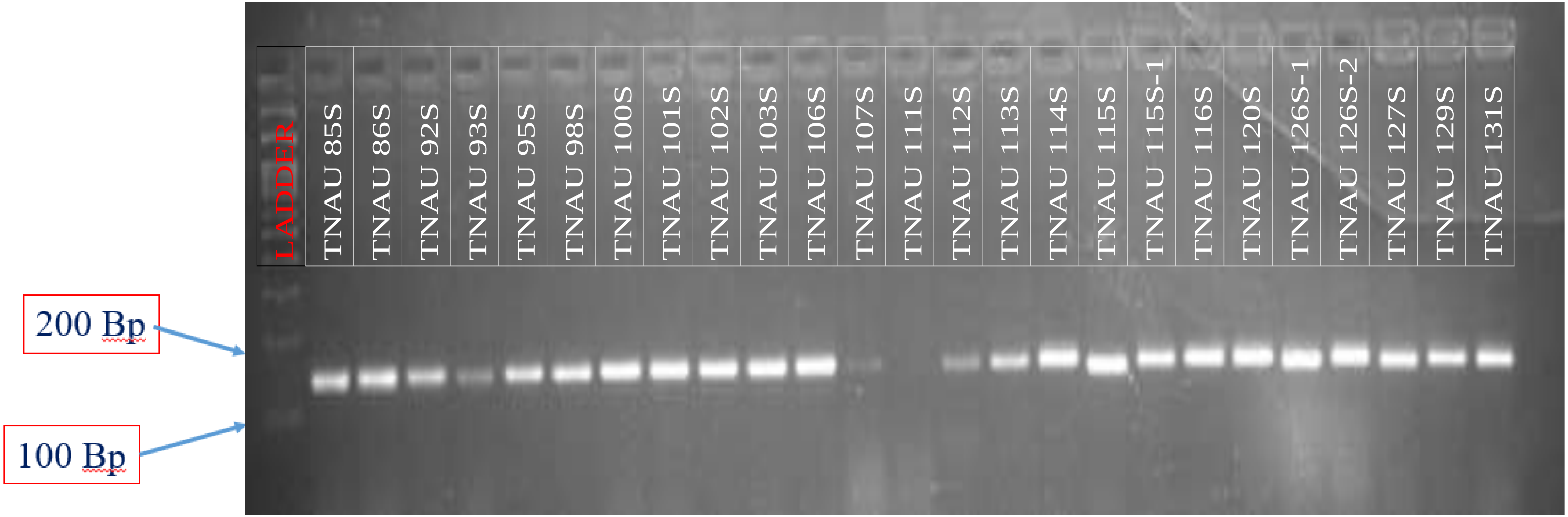

RM125
Chromosome-7

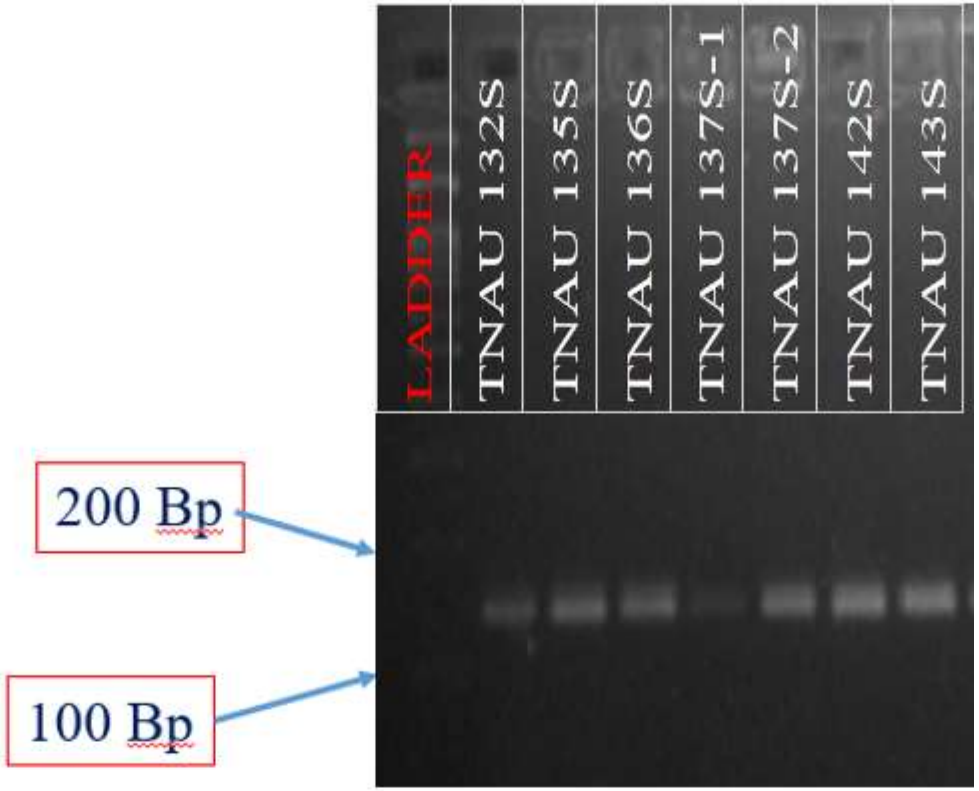

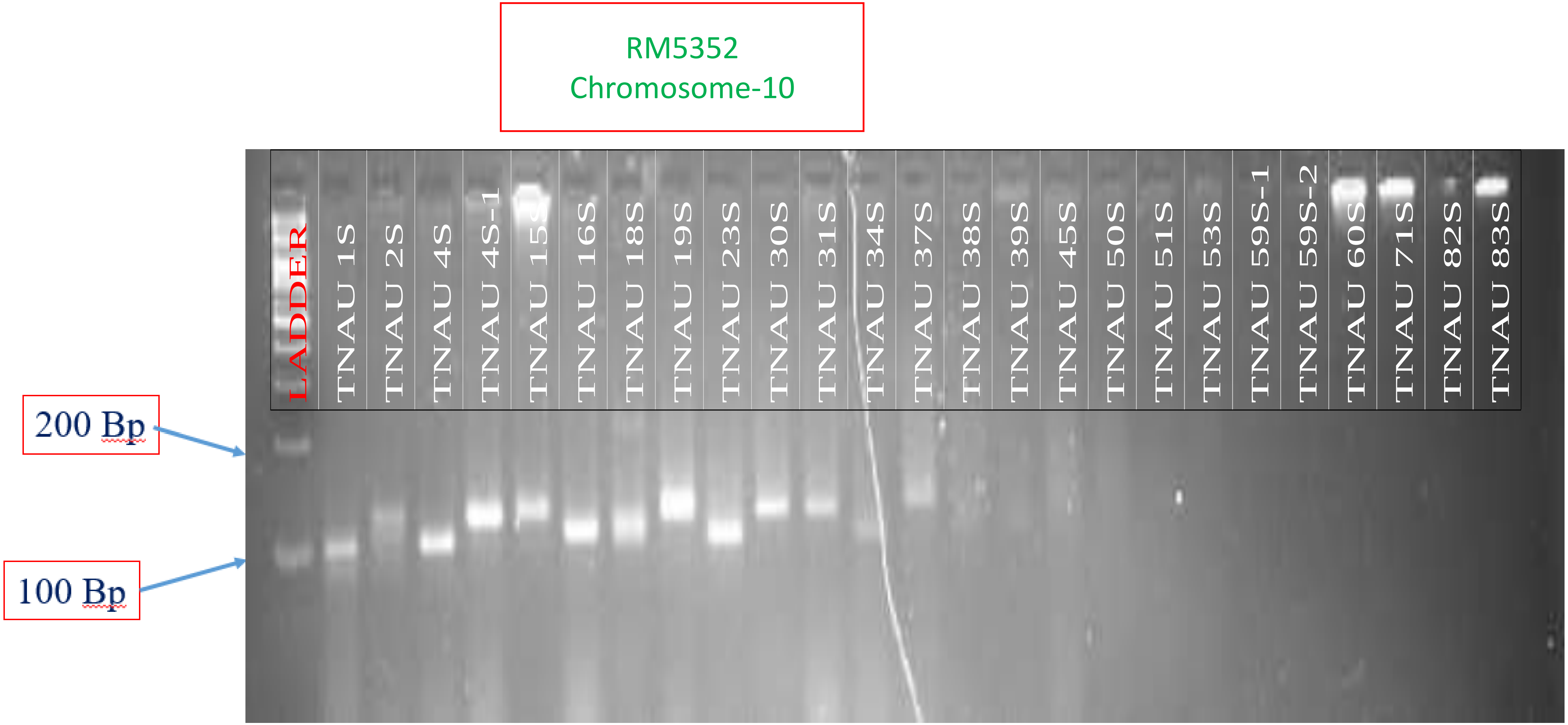

RM5352
Chromosome-10
LADDER
TNAU 1S
TNAU 2S
TNAU 4S
TNAU 4S-1
TNAU 15S
TNAU 16S
TNAU 18S
TNAU 19S
TNAU 23S
TNAU 30S
TNAU 31S
TNAU 34S
TNAU 37S
TNAU 38S
TNAU 39S
TNAU 45S
TNAU 50S
TNAU 51S
TNAU 53S
TNAU 59S-1
TNAU 59S-2
TNAU 60S
TNAU 71S
TNAU 82S
TNAU 83S
200 Bp
100 Bp

RM5352
Chromosome-10

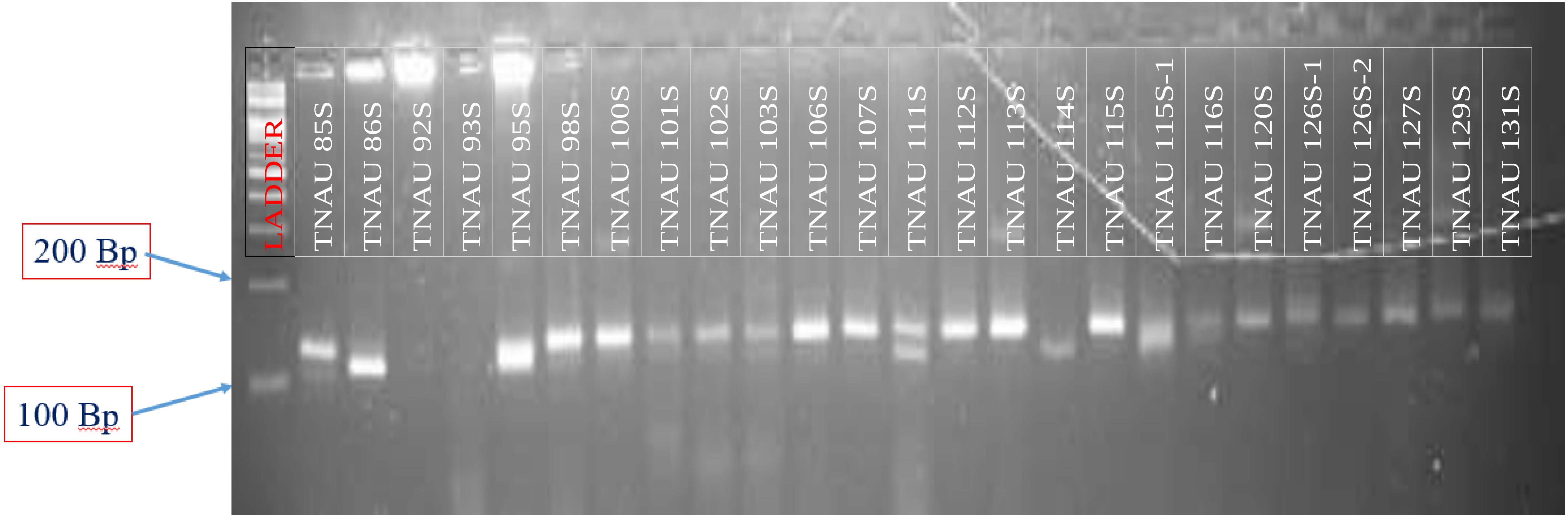

RM5352
Chromosome-10

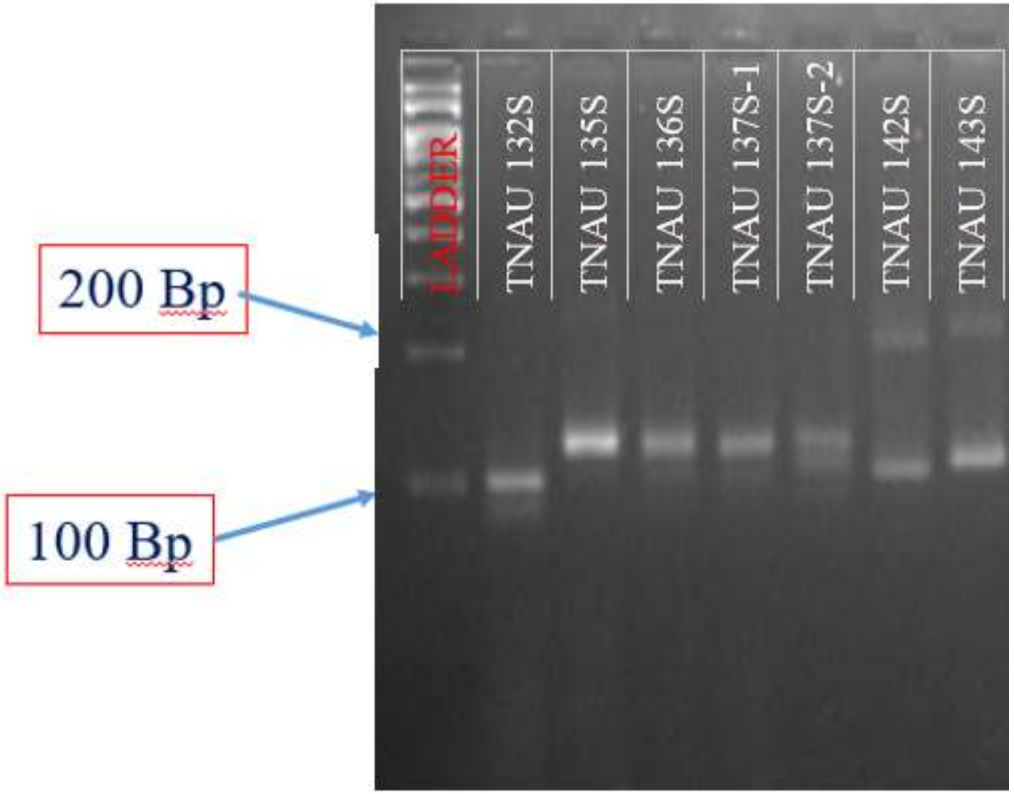

ch10 4455 55 116

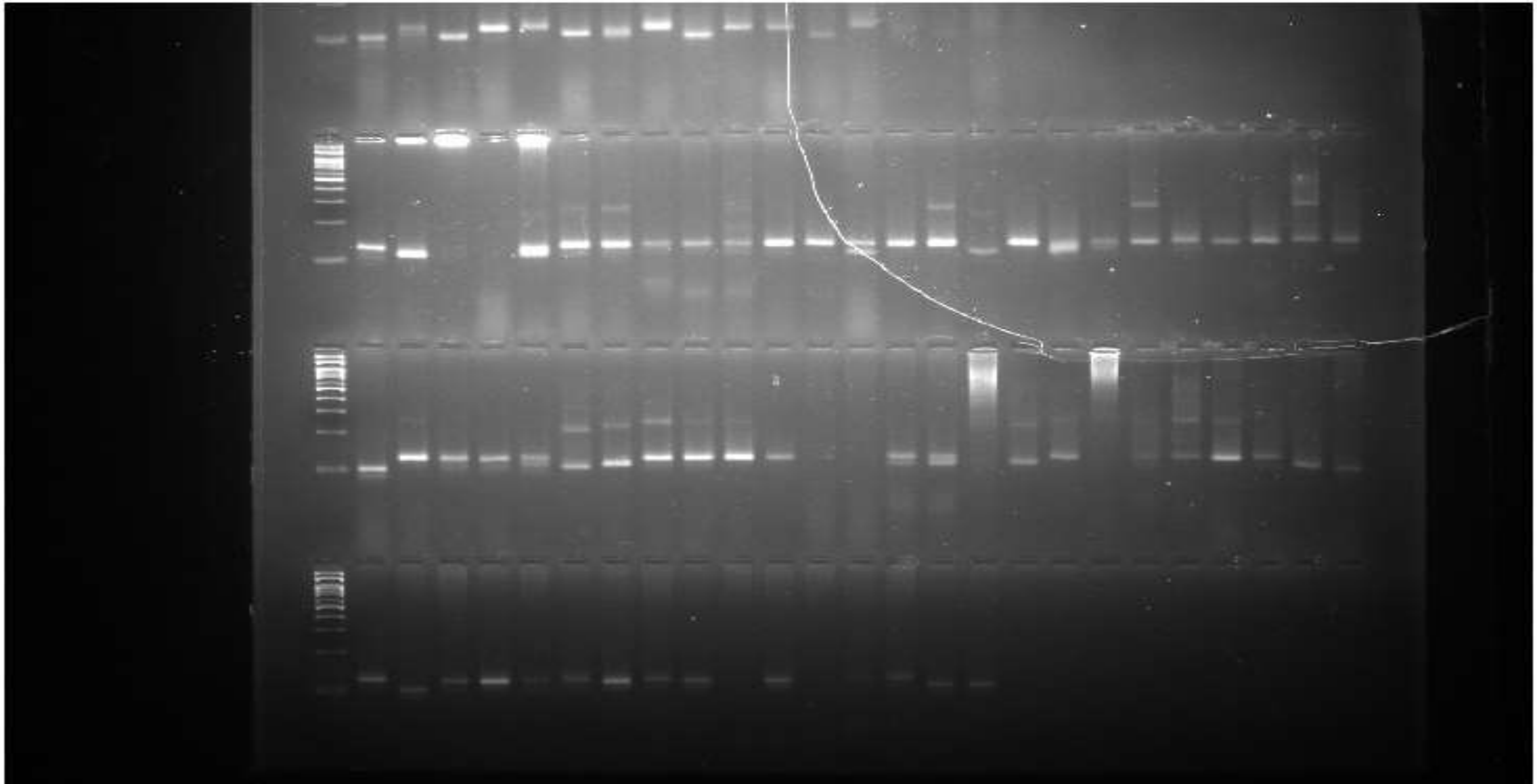

RM4455
Chromosome-10

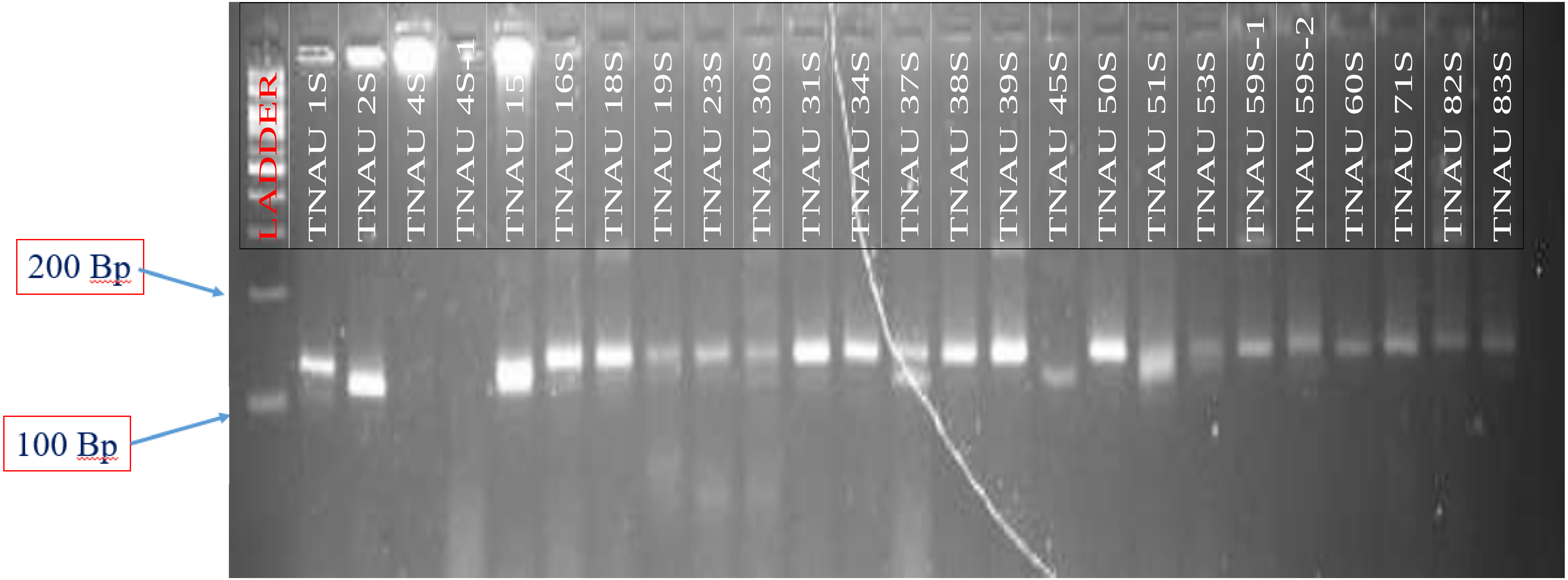

RM4455
Chromosome-10

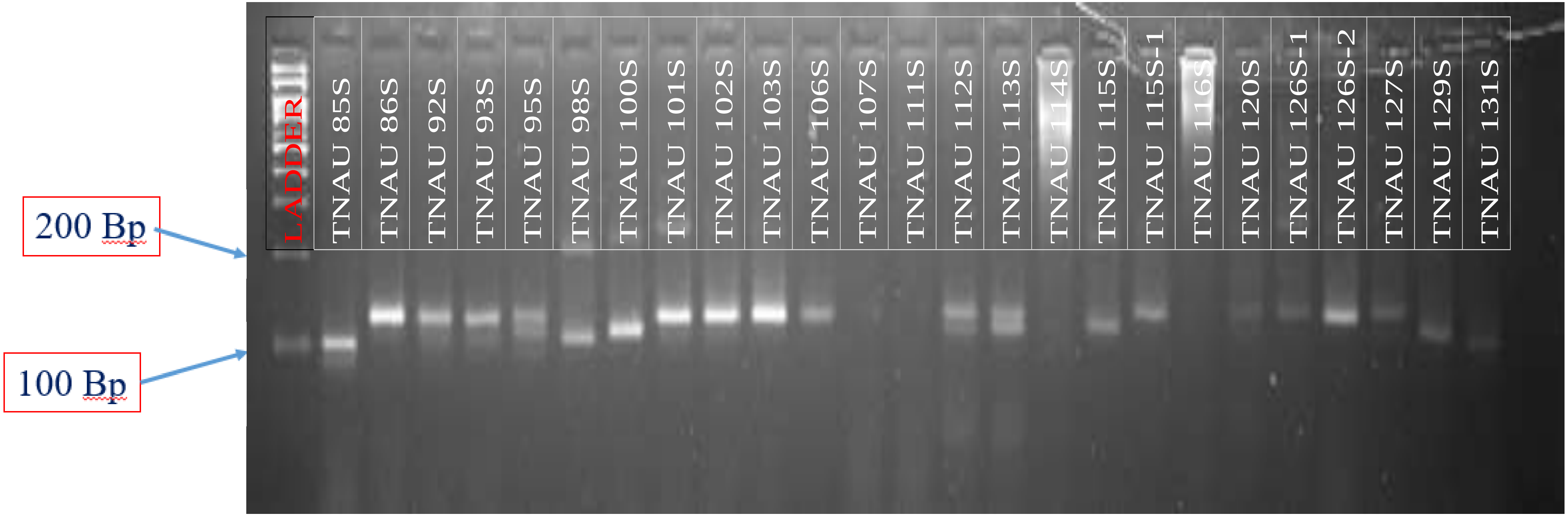

RM4455
Chromosome-10

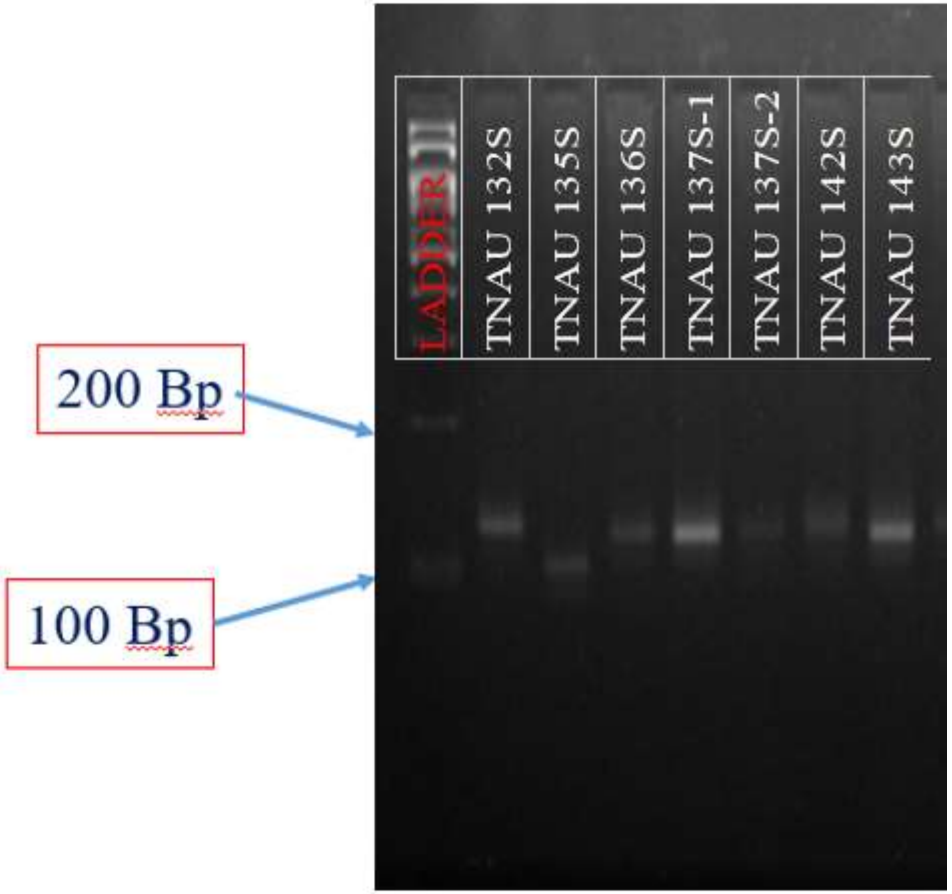

ch1 157 55 106

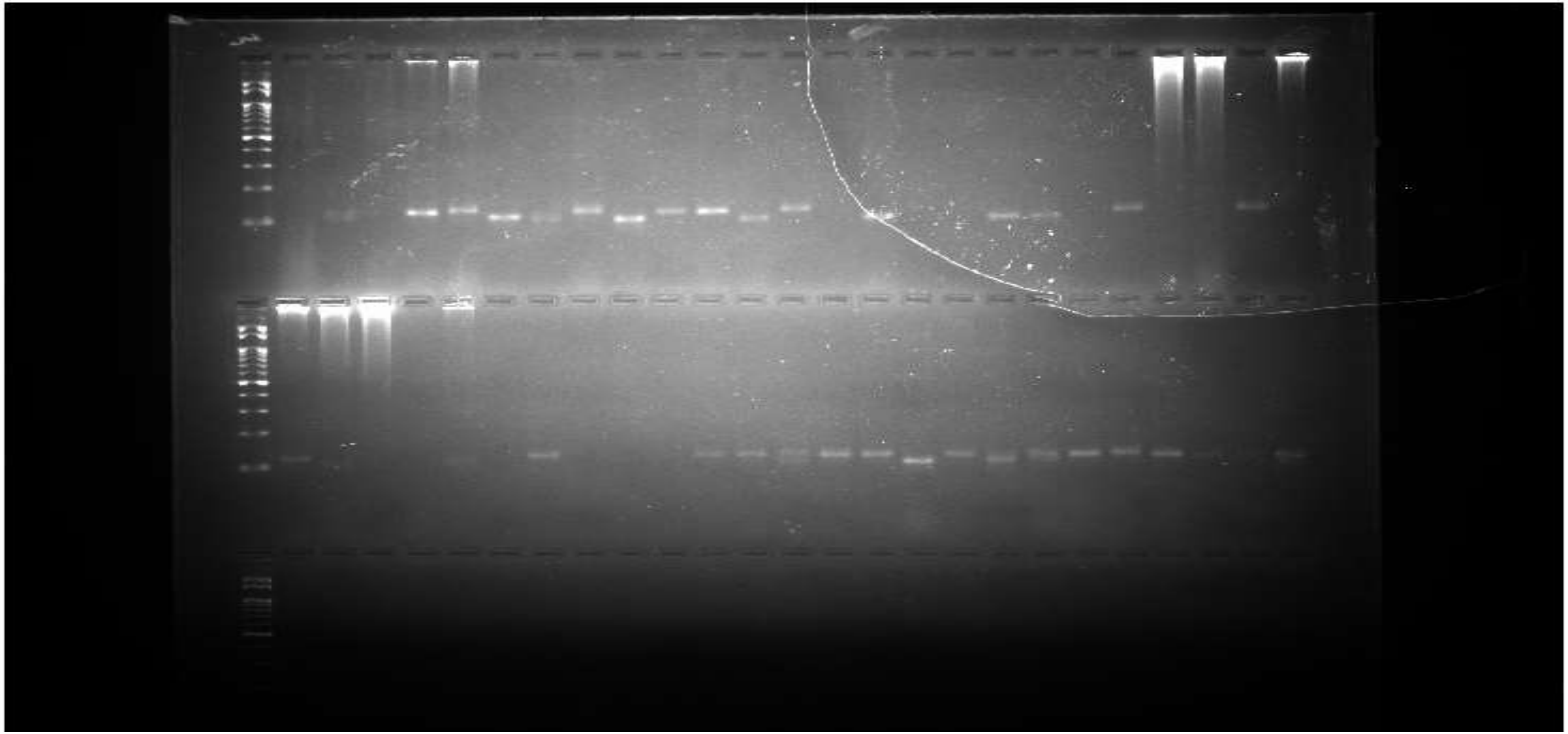

RM157
Chromosome-1

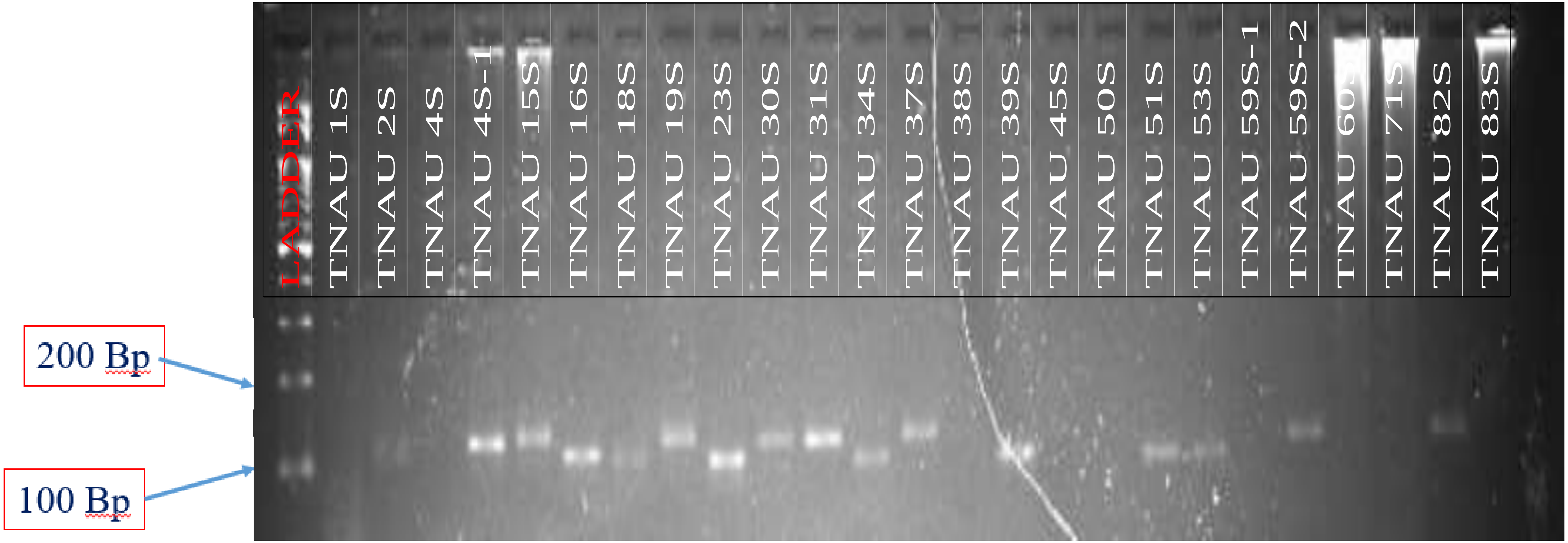

RM157
Chromosome-1

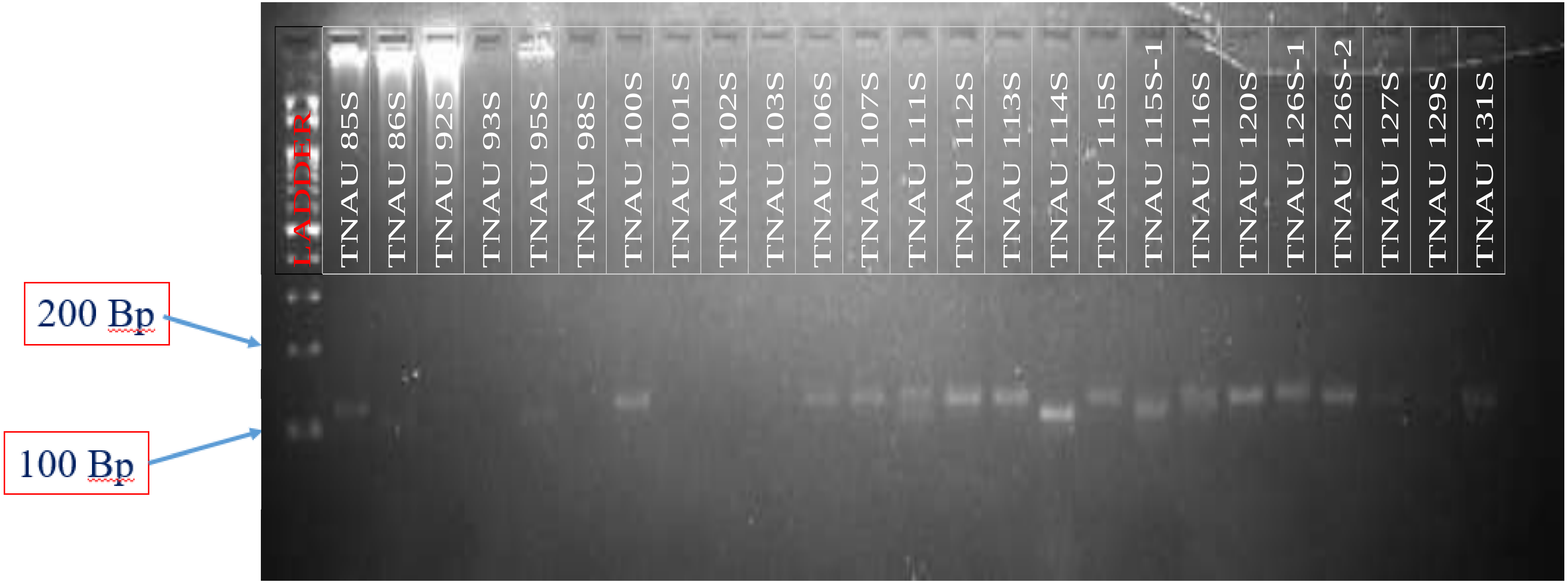

RM157
Chromosome-1

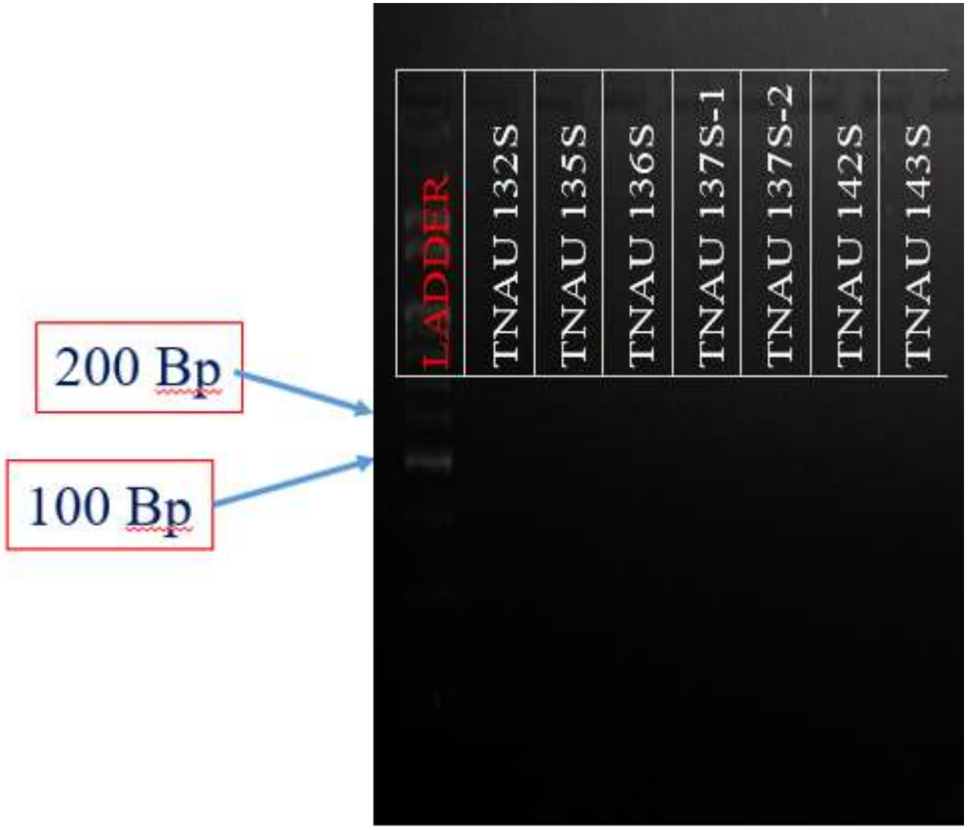

RM195
Chromosome-8

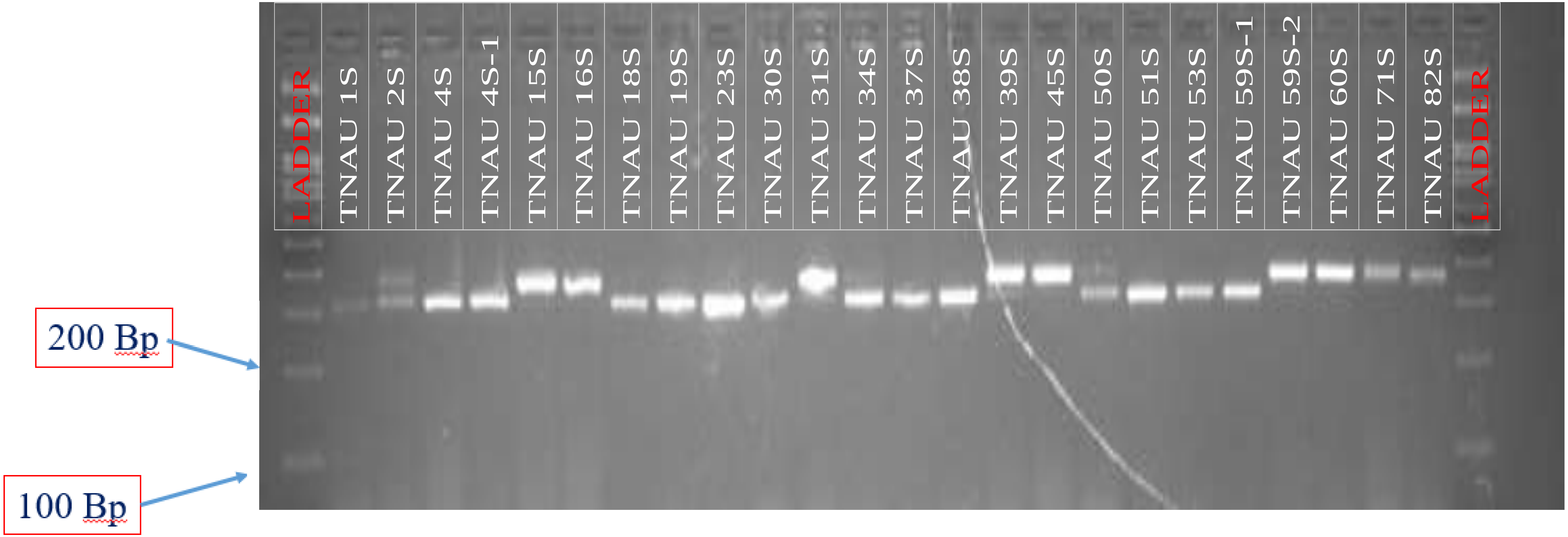

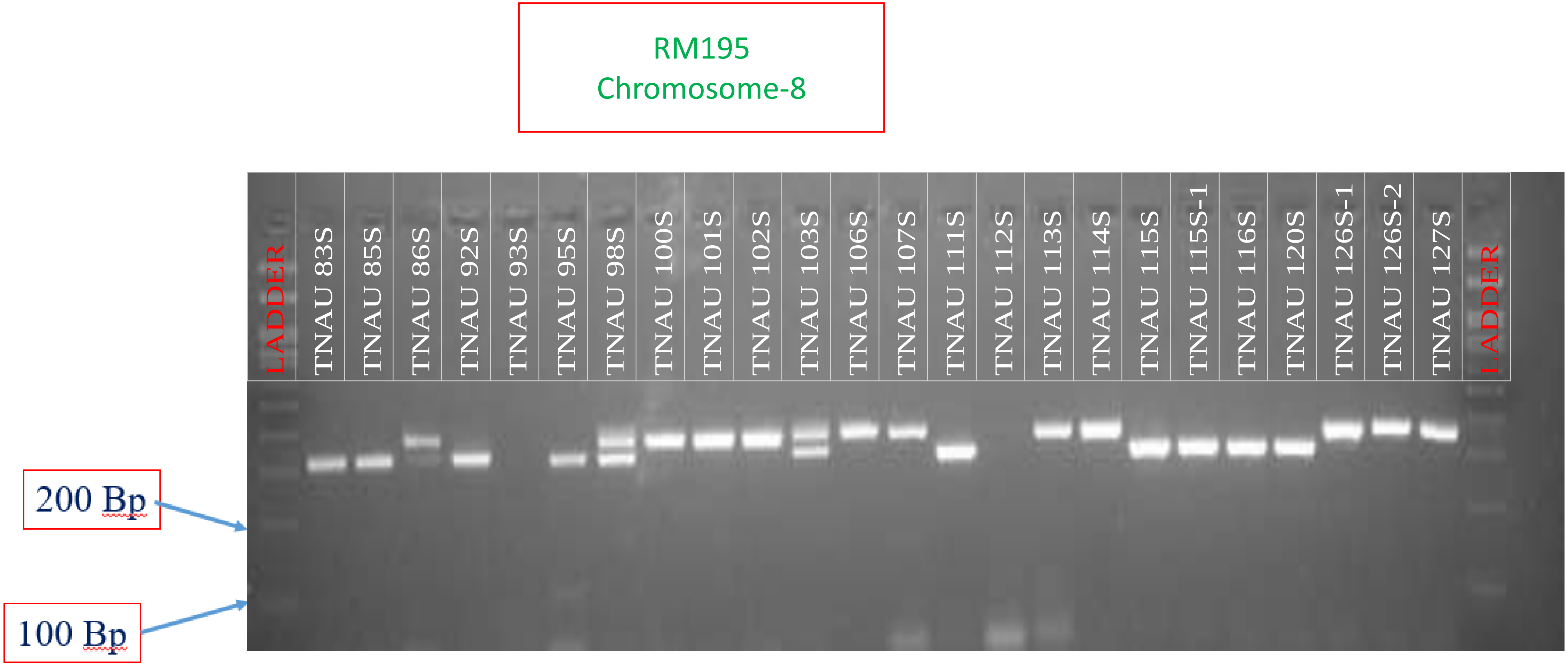
RM195
Chromosome-8
LADDER
TNAU 83S
TNAU 85S
TNAU 86S
TNAU 92S
TNAU 93S
TNAU 95S
TNAU 98S
TNAU 100S
TNAU 101S
TNAU 102S
TNAU 103S
TNAU 106S
TNAU 107S
TNAU 111S
TNAU 112S
TNAU 113S
TNAU 114S
TNAU 115S
TNAU 115S-1
TNAU 116S
TNAU 120S
TNAU 126S-1
TNAU 126S-2
TNAU 127S
LADDER
200 Bp
100 Bp

RM195
Chromosome-8

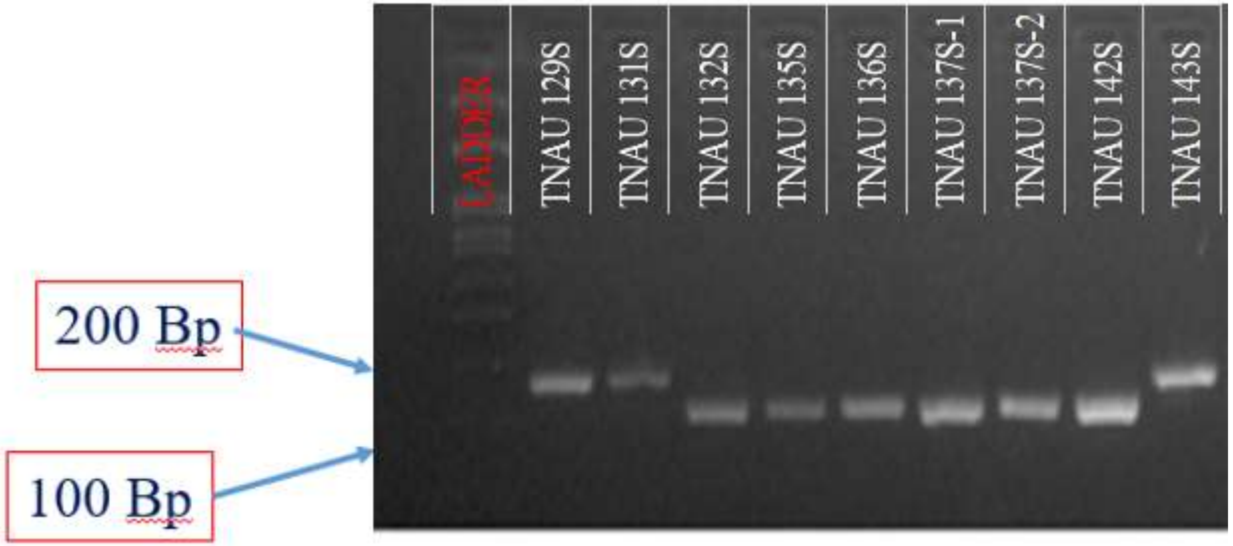

RM5704
Chromosome-11

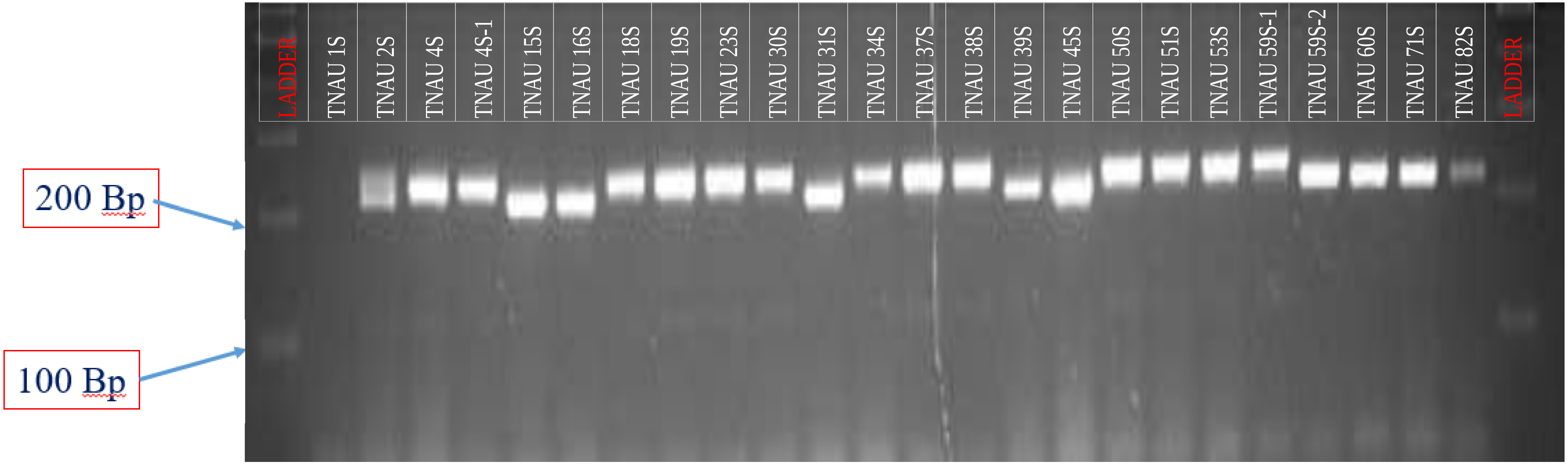

RM5704
Chromosome-11

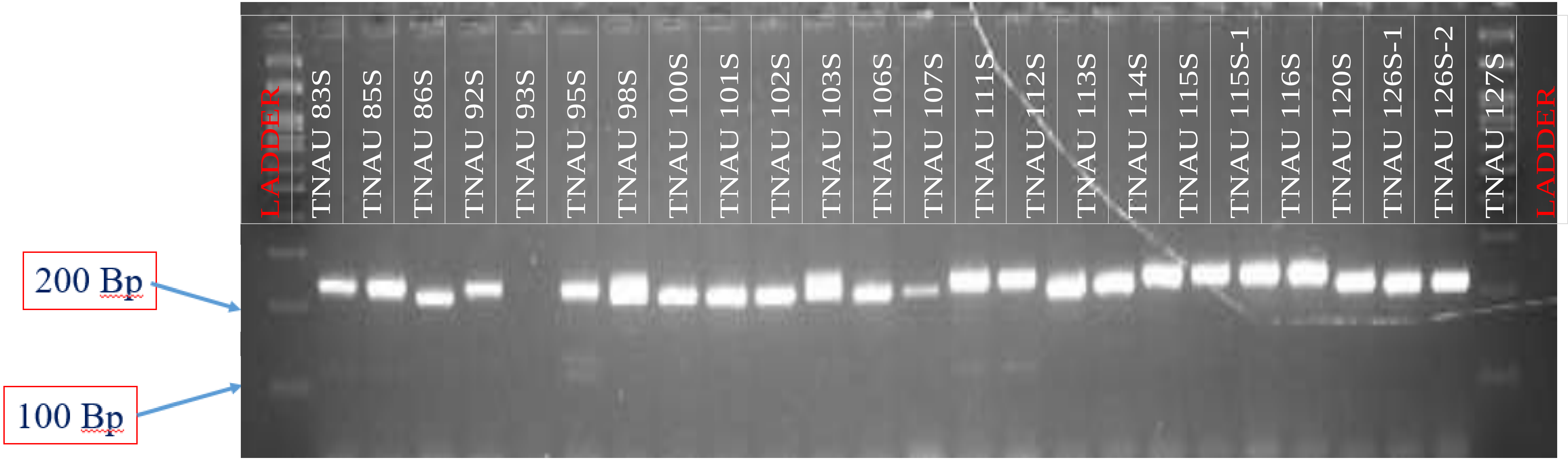

RM5704
Chromosome-11

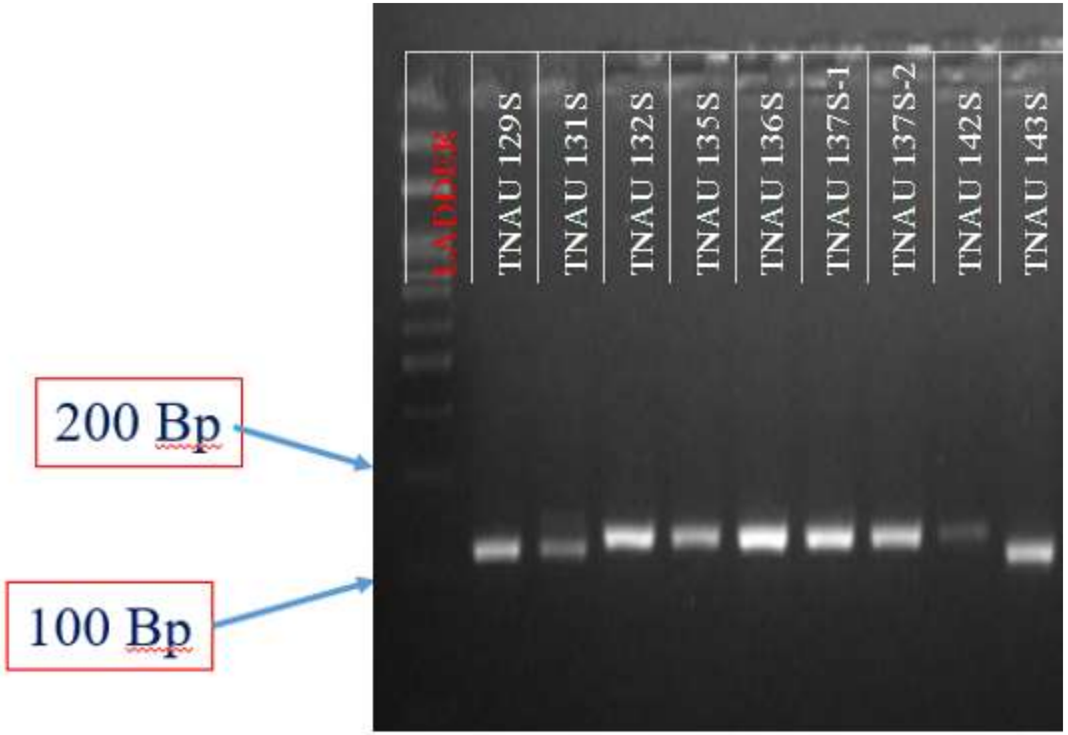

RM184
Chromosome-10

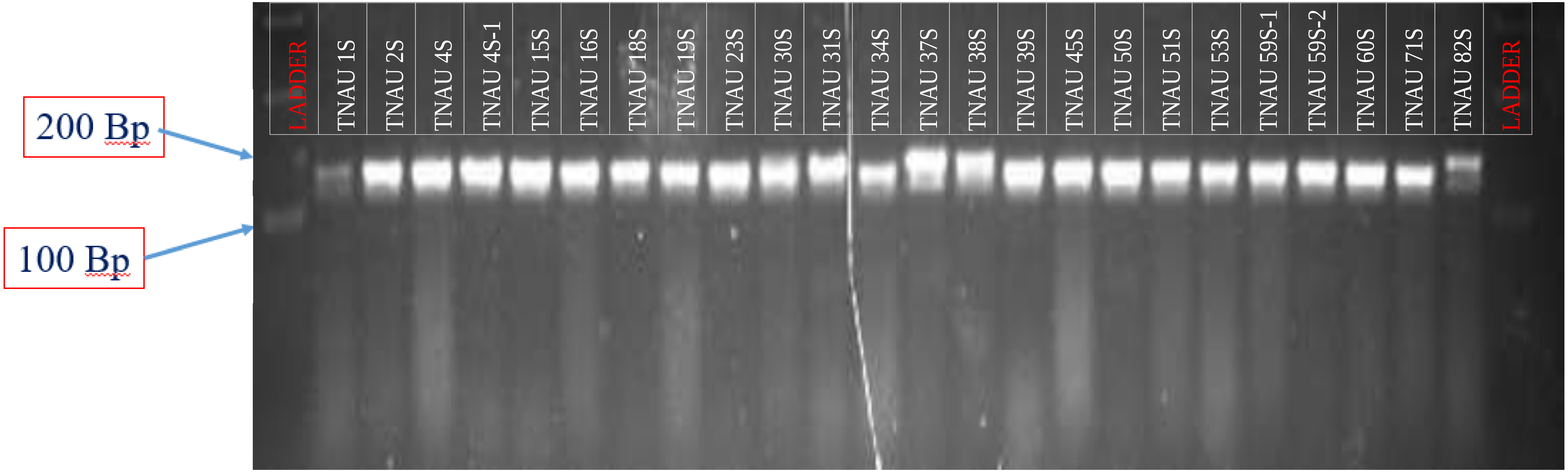

RM184
Chromosome-10

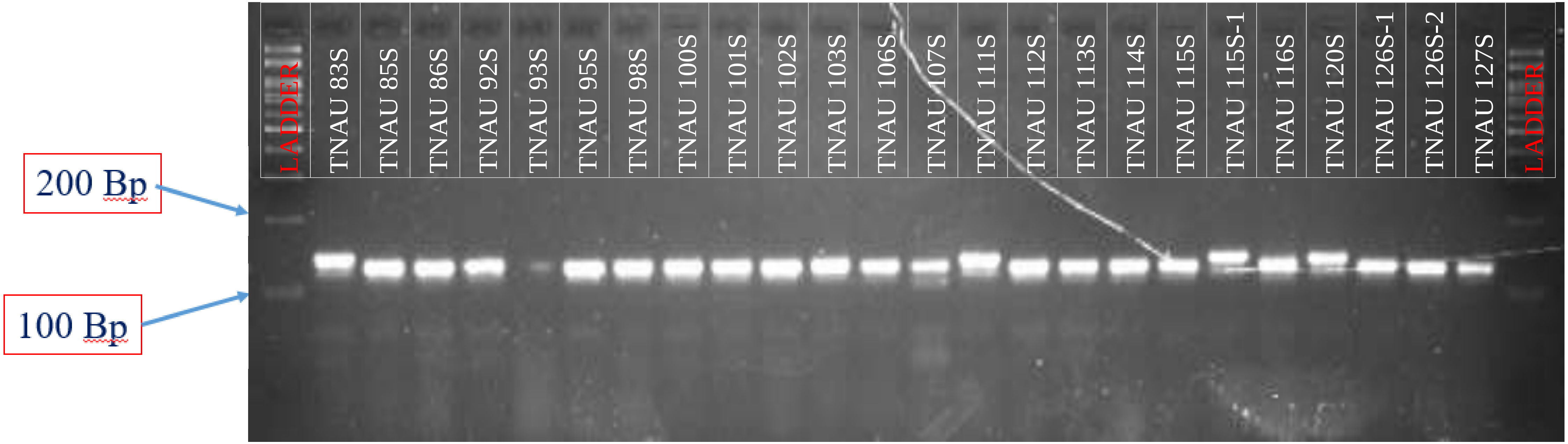

RM184
Chromosome-10

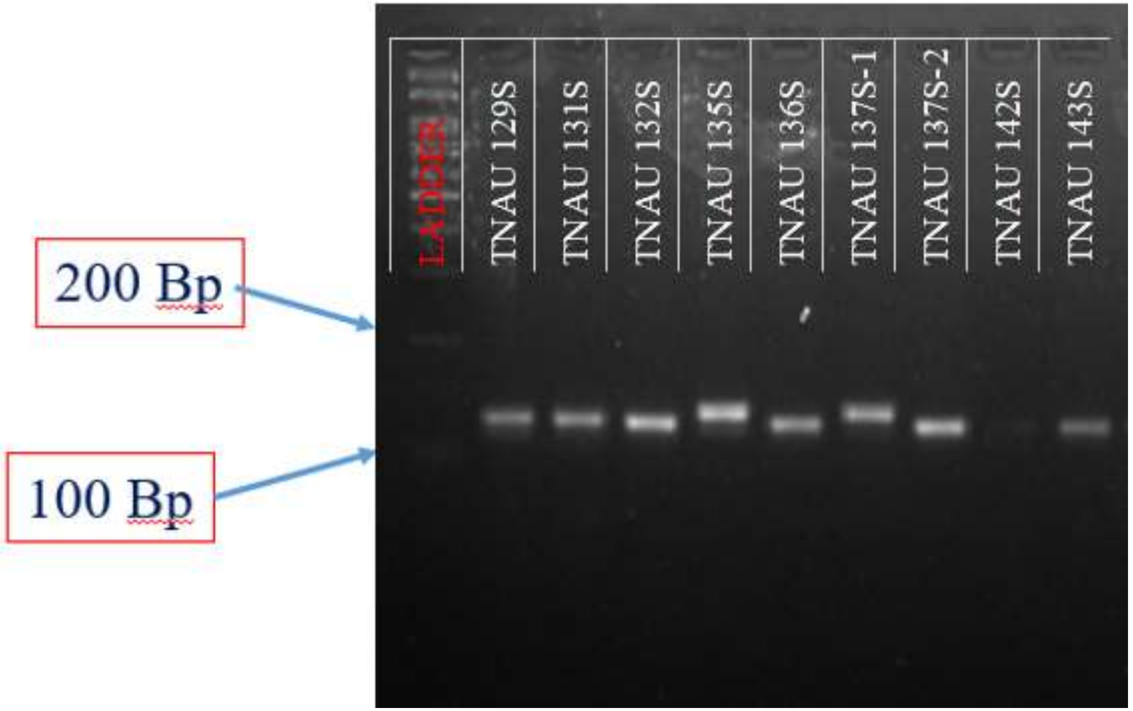

RM433
Chromosome-8

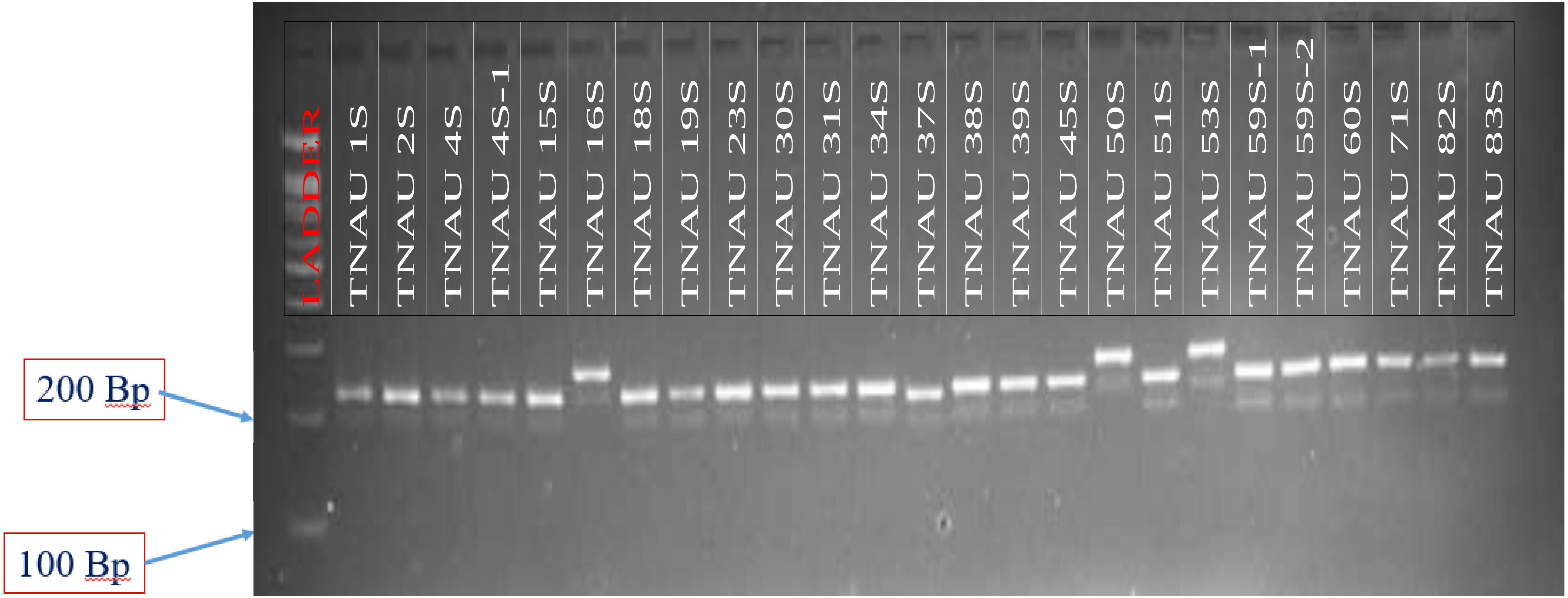

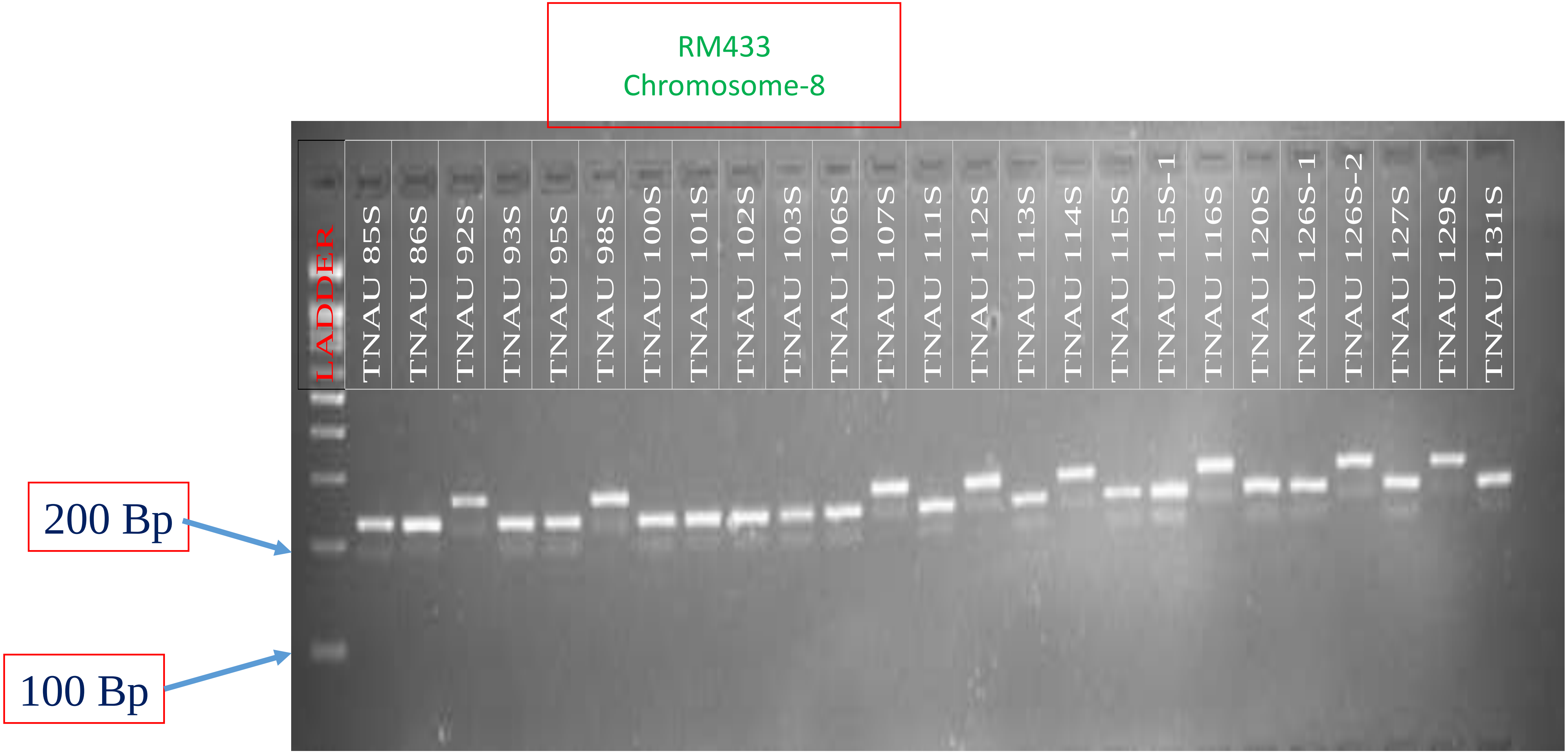
RM433
Chromosome-8
LADDER
TNAU 85S
TNAU 86S
TNAU 92S
TNAU 93S
TNAU 95S
TNAU 98S
TNAU 100S
TNAU 101S
TNAU 102S
TNAU 103S
TNAU 106S
TNAU 107S
TNAU 111S
TNAU 112S
TNAU 113S
TNAU 114S
TNAU 115S
TNAU 115S-1
TNAU 116S
TNAU 120S
TNAU 126S-1
TNAU 126S-2
TNAU 127S
TNAU 129S
TNAU 131S
200 Bp
100 Bp

RM433
Chromosome-8

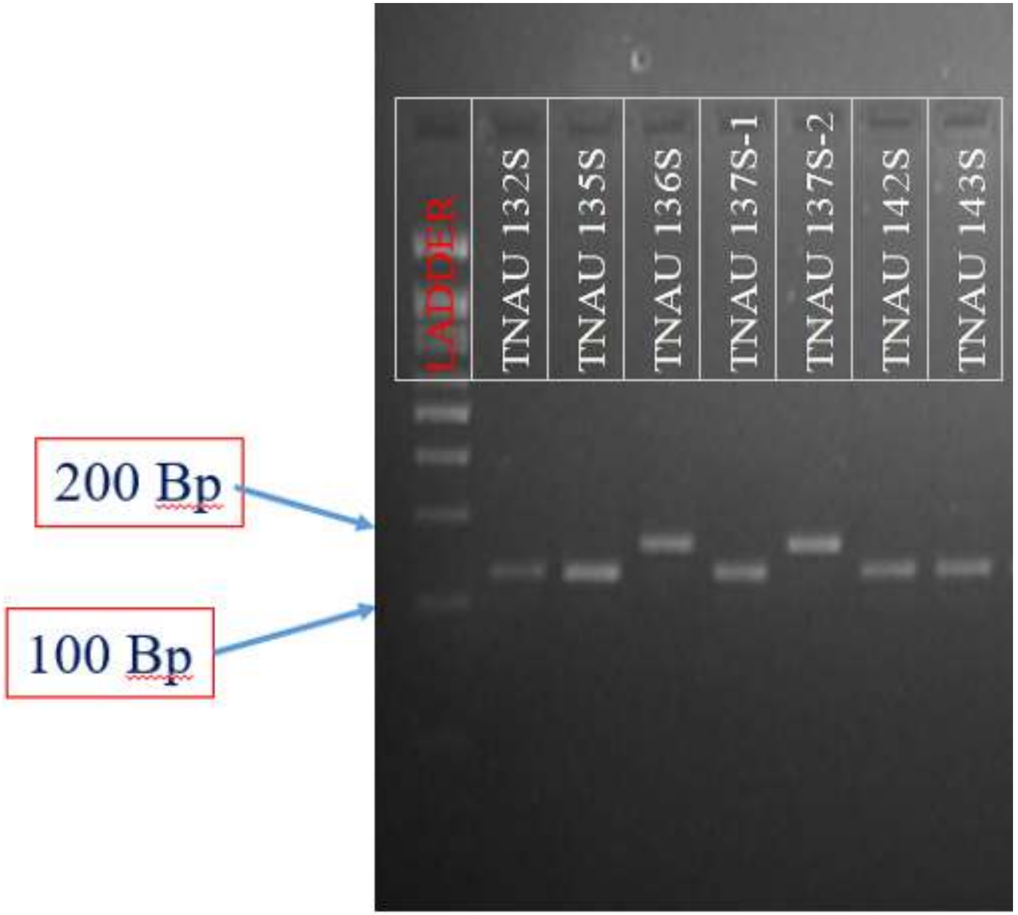

RM423
Chromosome-7

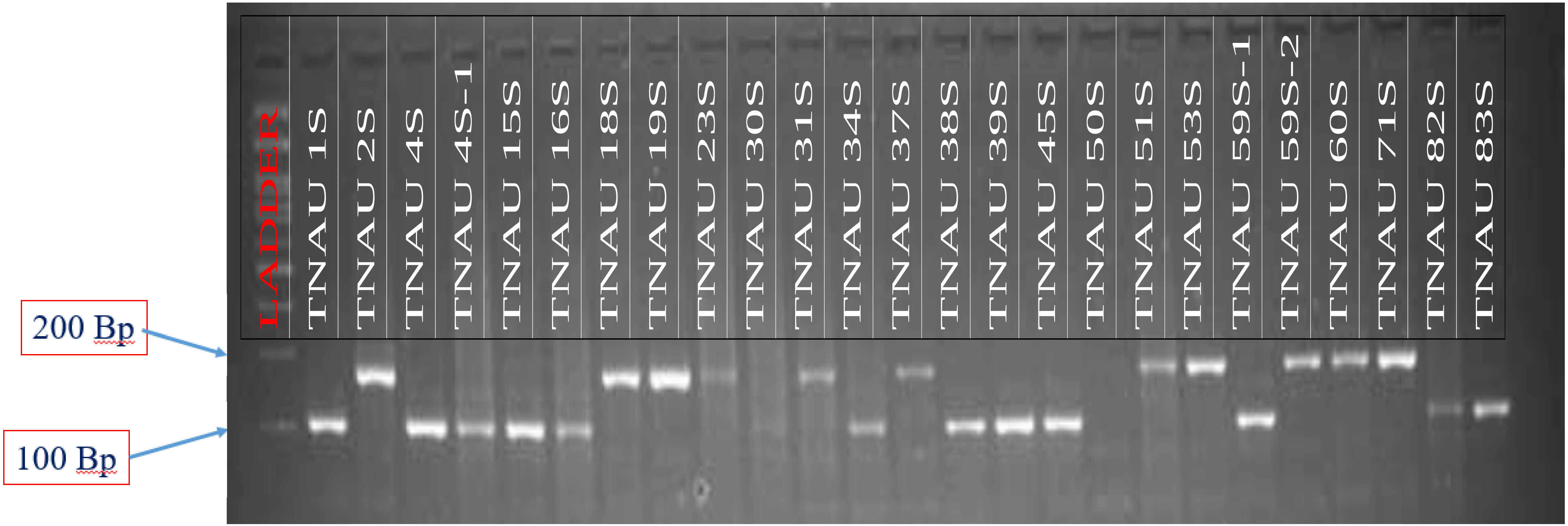

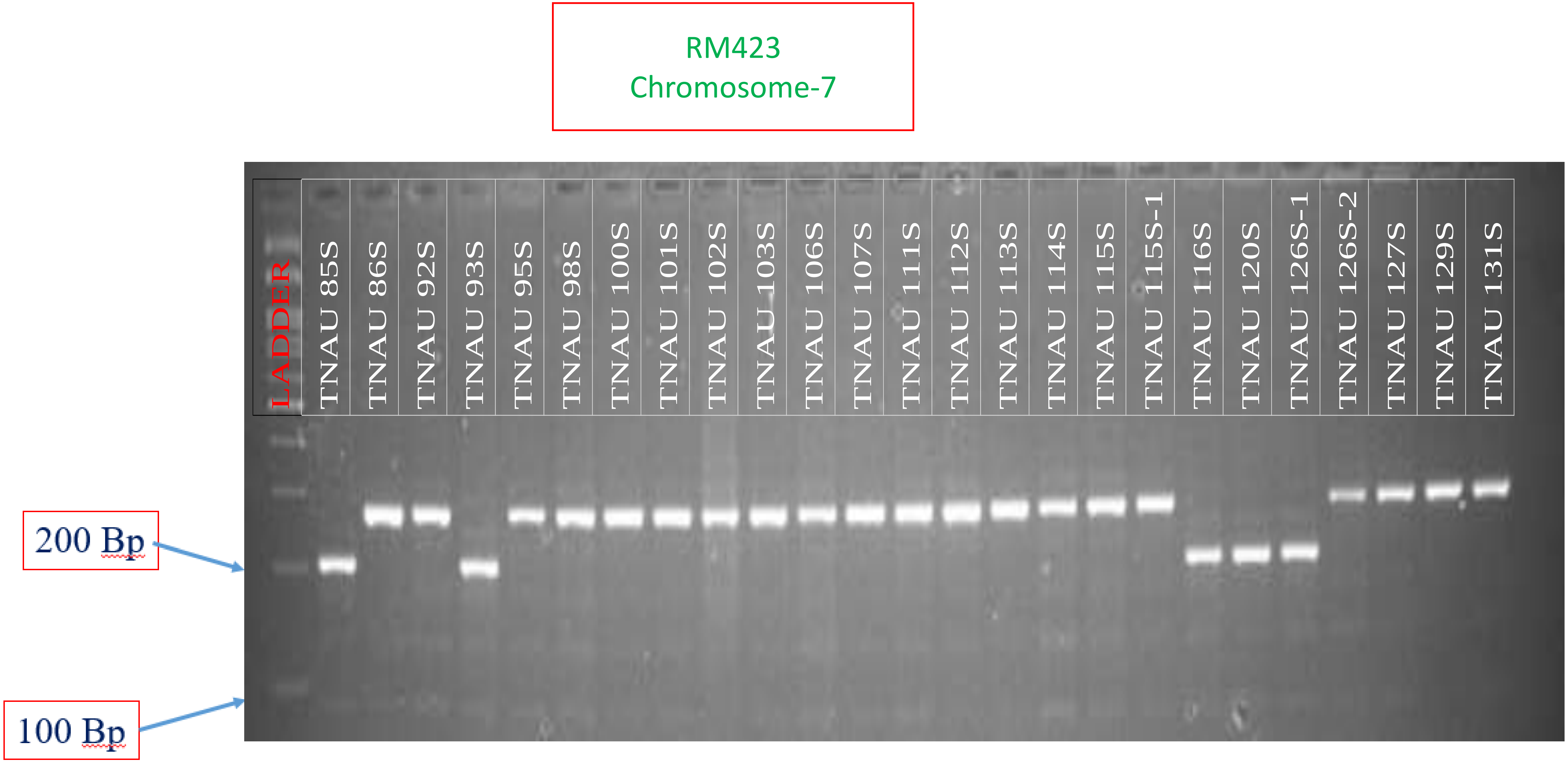

RM423
Chromosome-7
LADDER
TNAU 85S
TNAU 86S
TNAU 92S
TNAU 93S
TNAU 95S
TNAU 98S
TNAU 100S
TNAU 101S
TNAU 102S
TNAU 103S
TNAU 106S
TNAU 107S
TNAU 111S
TNAU 112S
TNAU 113S
TNAU 114S
TNAU 115S
TNAU 115S-1
TNAU 116S
TNAU 120S
TNAU 126S-1
TNAU 126S-2
TNAU 127S
TNAU 129S
TNAU 131S
200 Bp
100 Bp

RM423
Chromosome-7

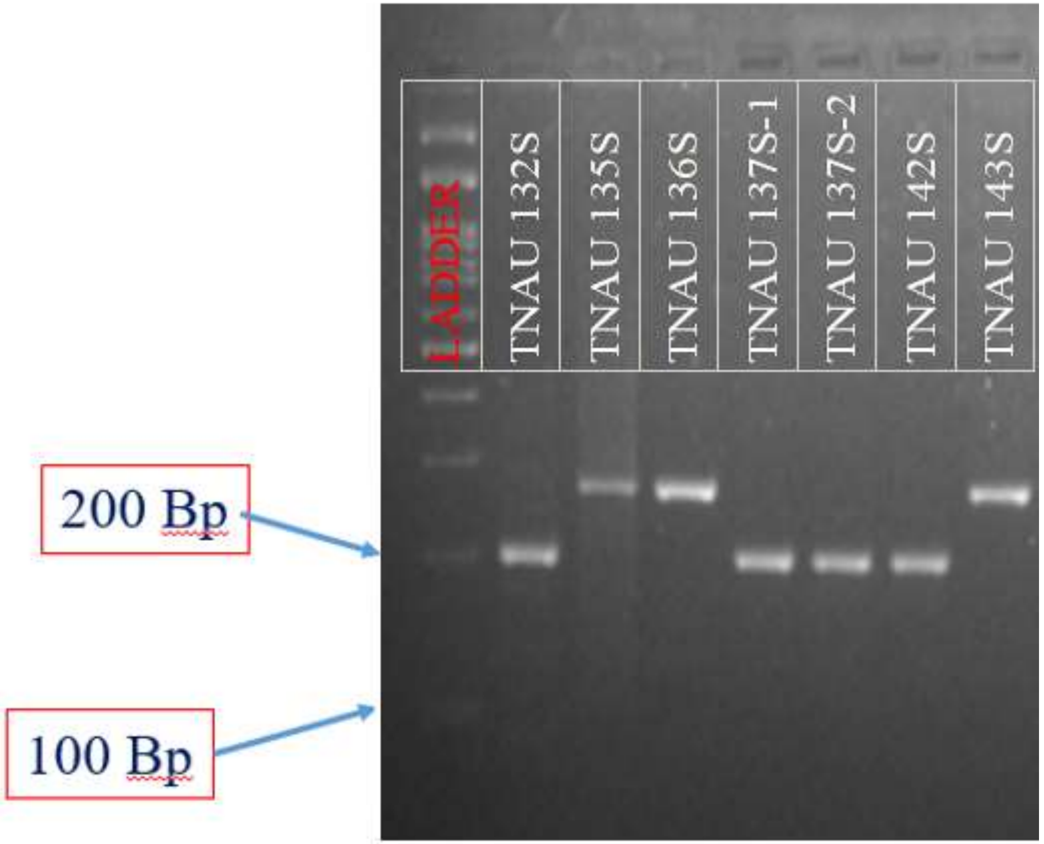

RM511
Chromosome-12

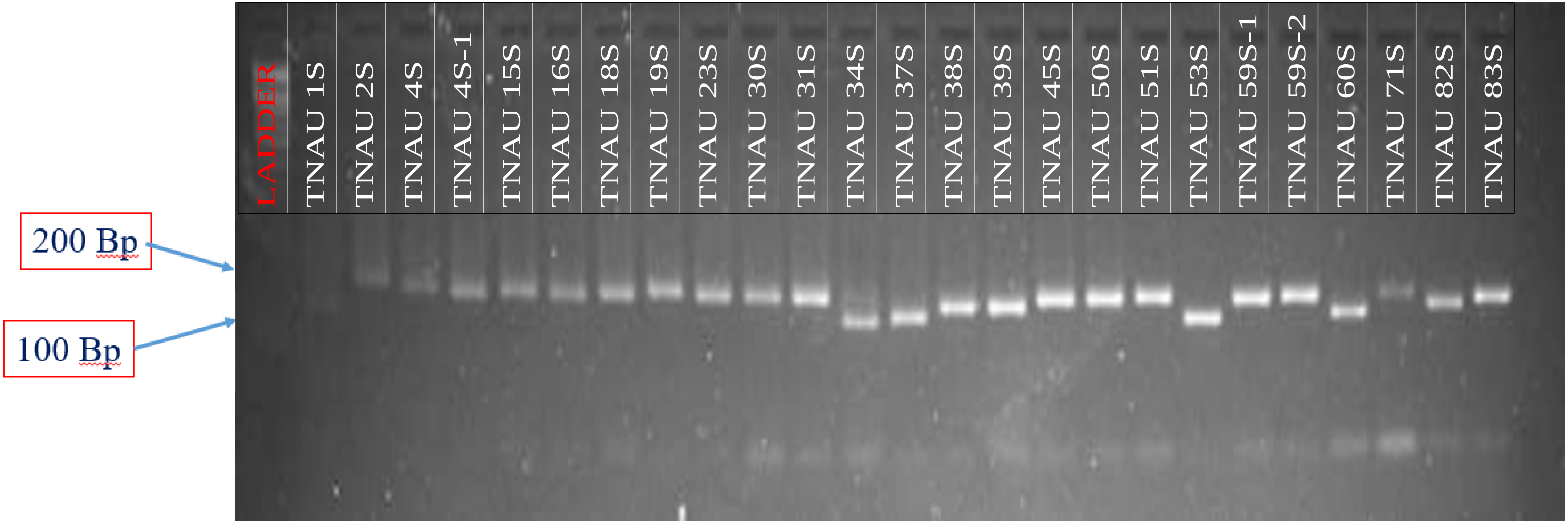

RM511
Chromosome-12

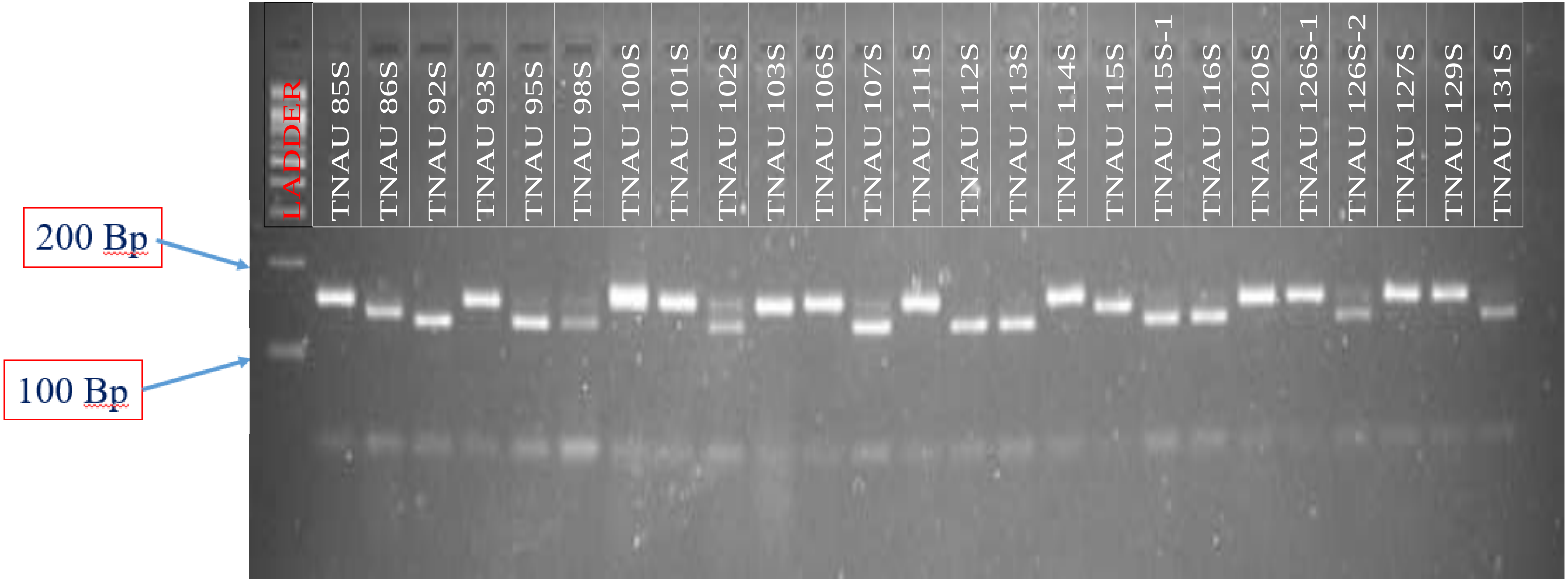

RM511
Chromosome-12

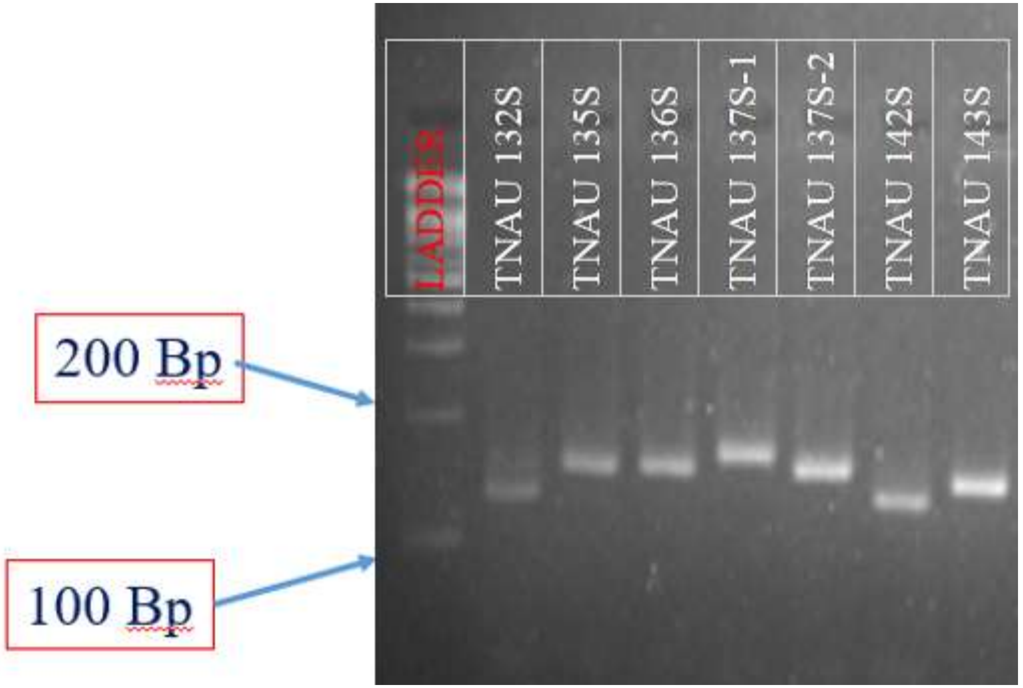

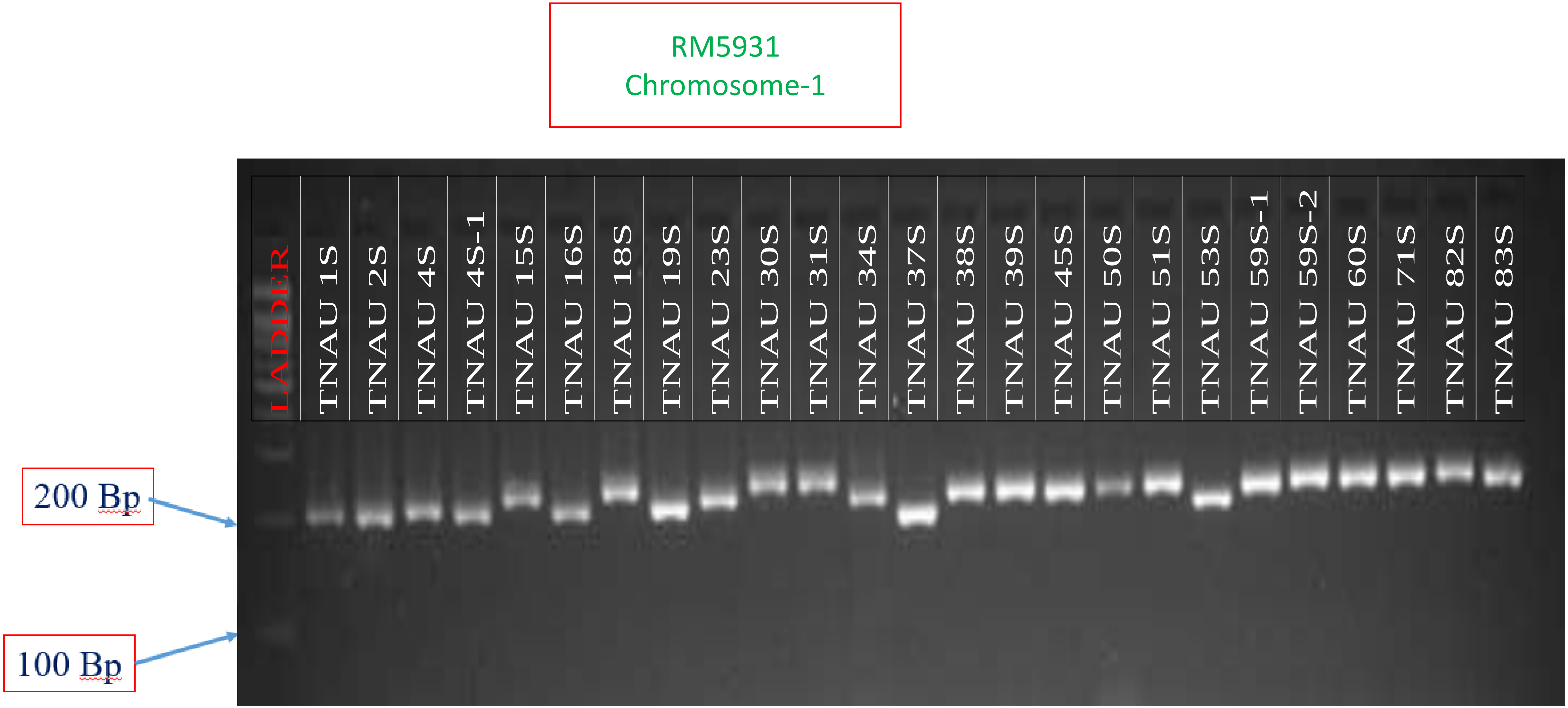
RM5931
Chromosome-1
LADDER
TNAU 1S
TNAU 2S
TNAU 4S
TNAU 4S-1
TNAU 15S
TNAU 16S
TNAU 18S
TNAU 19S
TNAU 23S
TNAU 30S
TNAU 31S
TNAU 34S
TNAU 37S
TNAU 38S
TNAU 39S
TNAU 45S
TNAU 50S
TNAU 51S
TNAU 53S
TNAU 59S-1
TNAU 59S-2
TNAU 60S
TNAU 71S
TNAU 82S
TNAU 83S
200 Bp
100 Bp

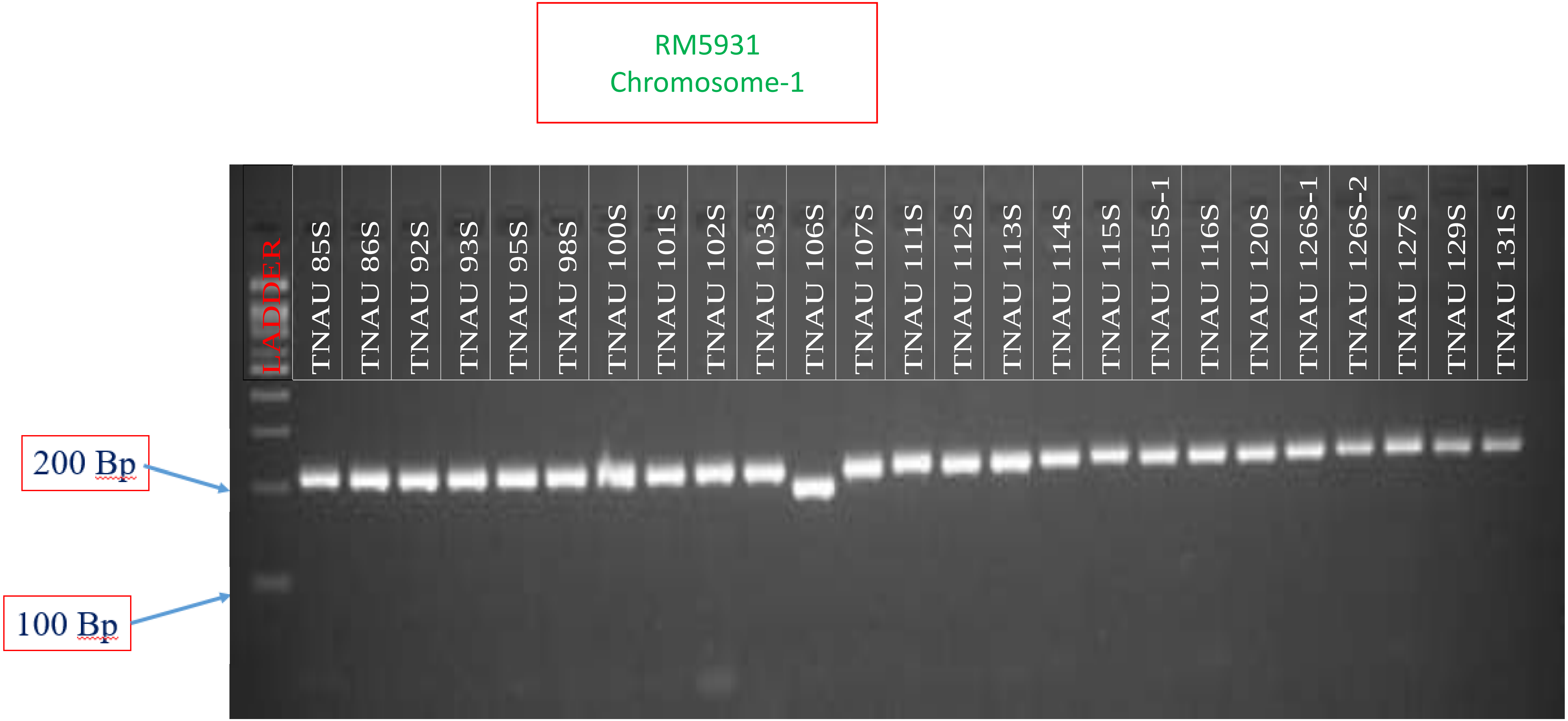
RM5931
Chromosome-1
LADDER
TNAU 85S
TNAU 86S
TNAU 92S
TNAU 93S
TNAU 95S
TNAU 98S
TNAU 100S
TNAU 101S
TNAU 102S
TNAU 103S
TNAU 106S
TNAU 107S
TNAU 111S
TNAU 112S
TNAU 113S
TNAU 114S
TNAU 115S
TNAU 115S-1
TNAU 116S
TNAU 120S
TNAU 126S-1
TNAU 126S-2
TNAU 127S
TNAU 129S
TNAU 131S
200 Bp
100 Bp

RM5931
Chromosome-1

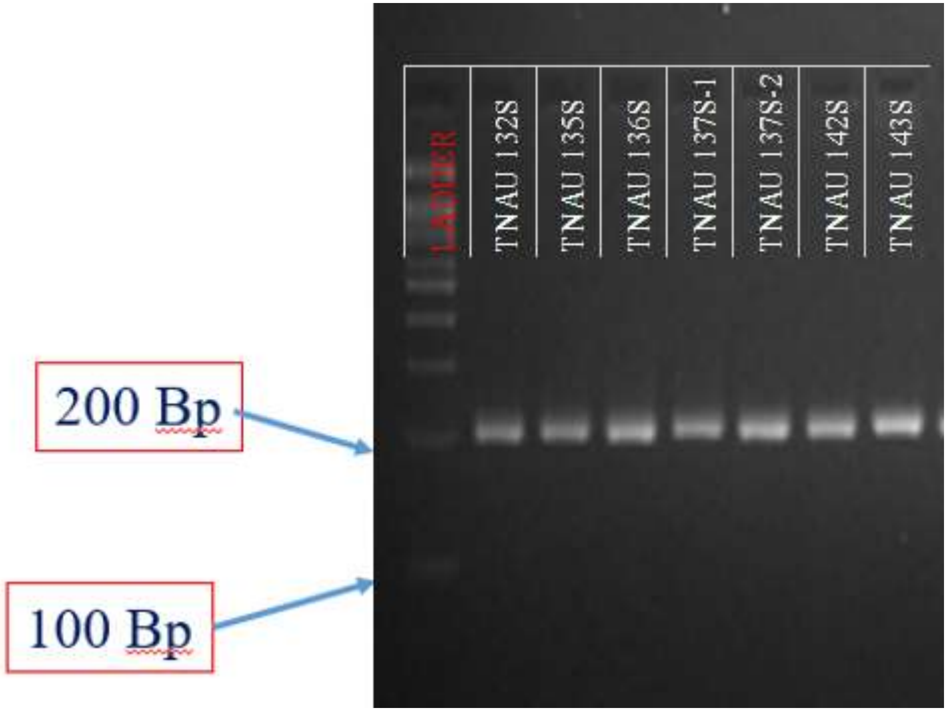

RM349
Chromosome-4

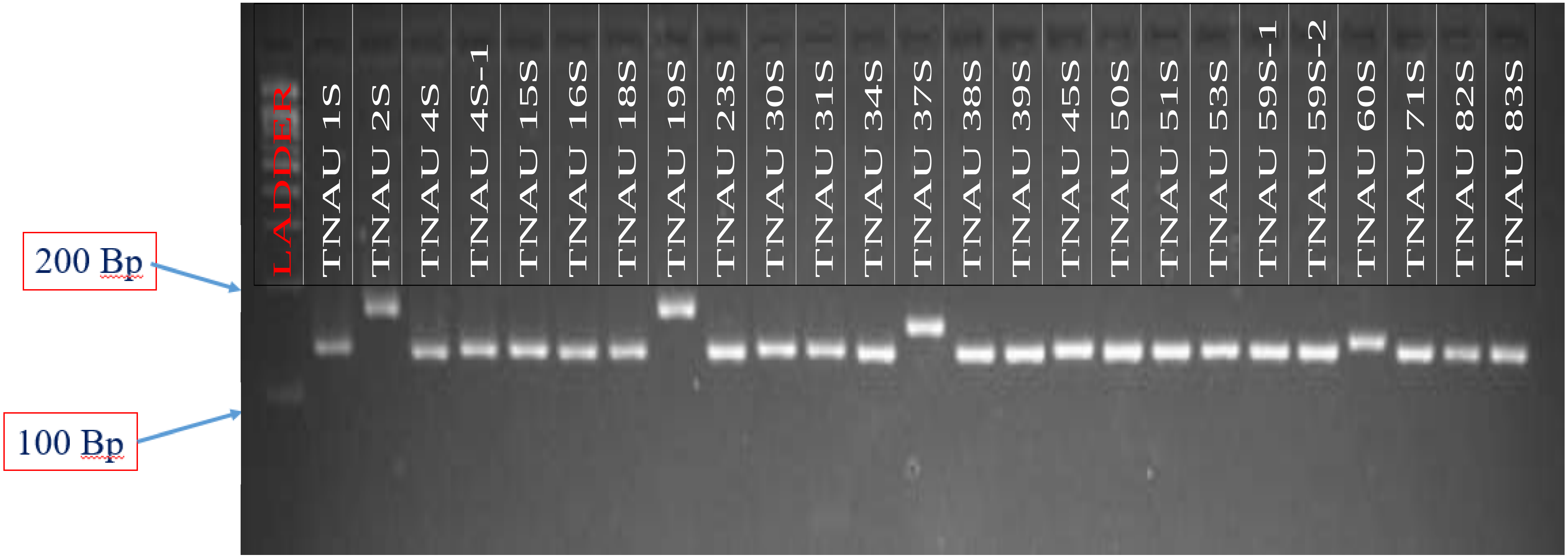

RM349
Chromosome-4

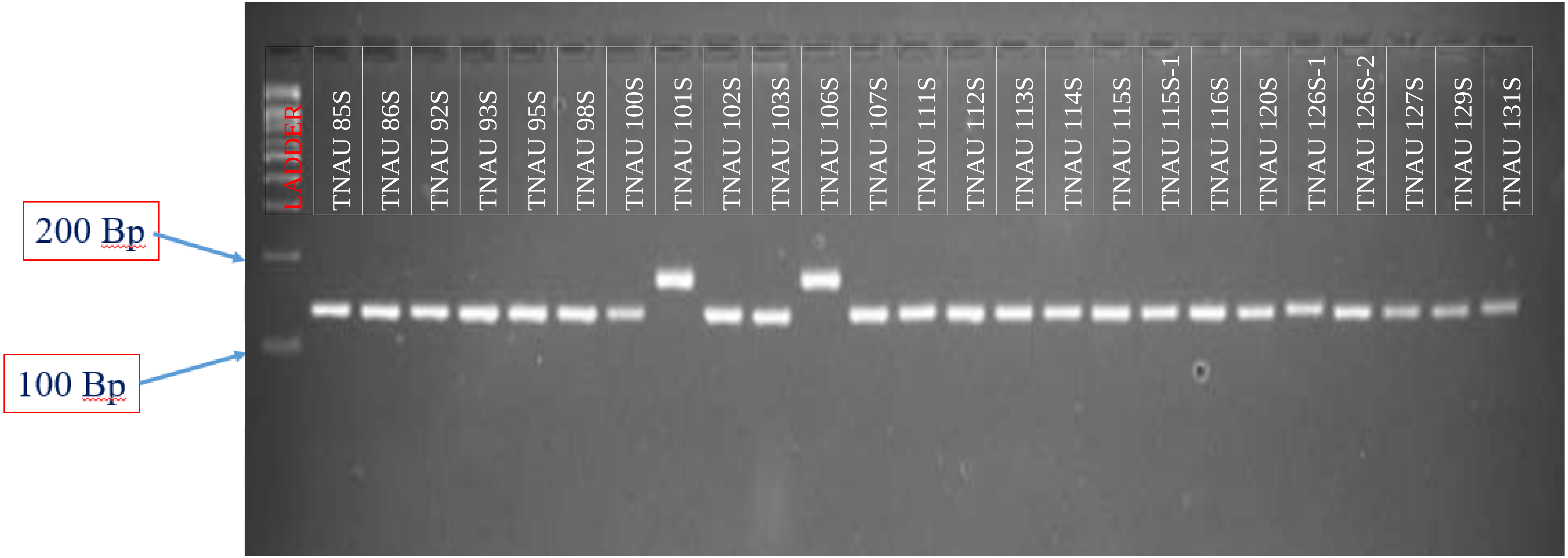

RM349
Chromosome-4

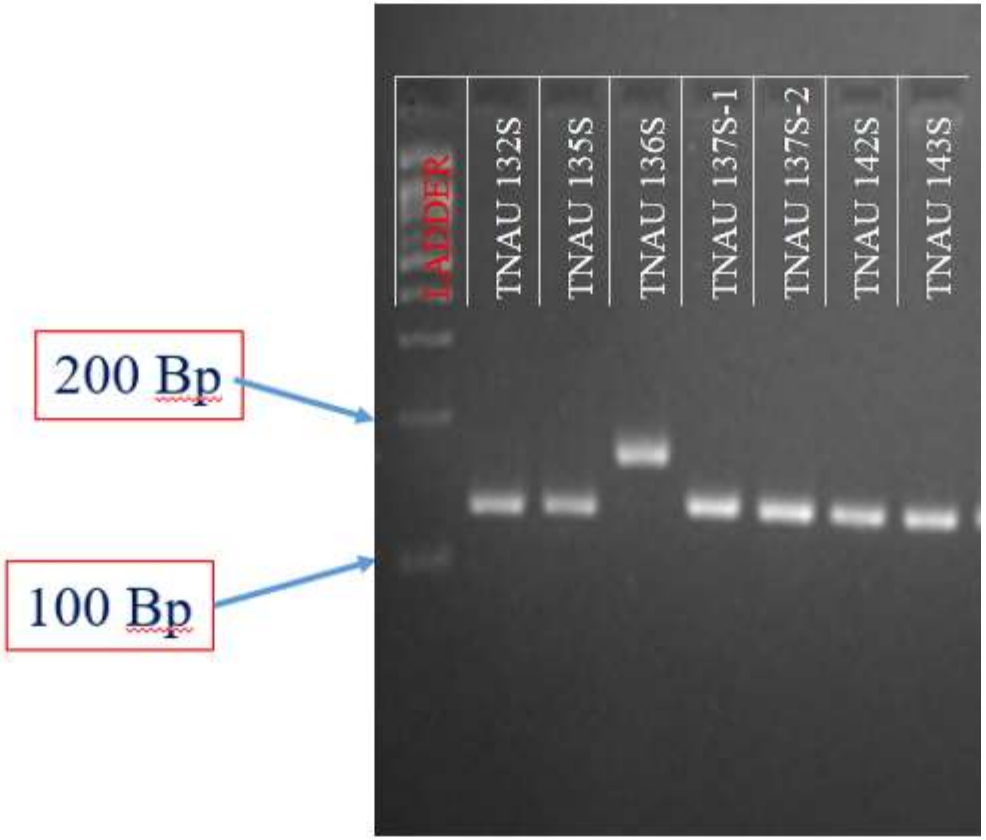

RM521
Chromosome-2

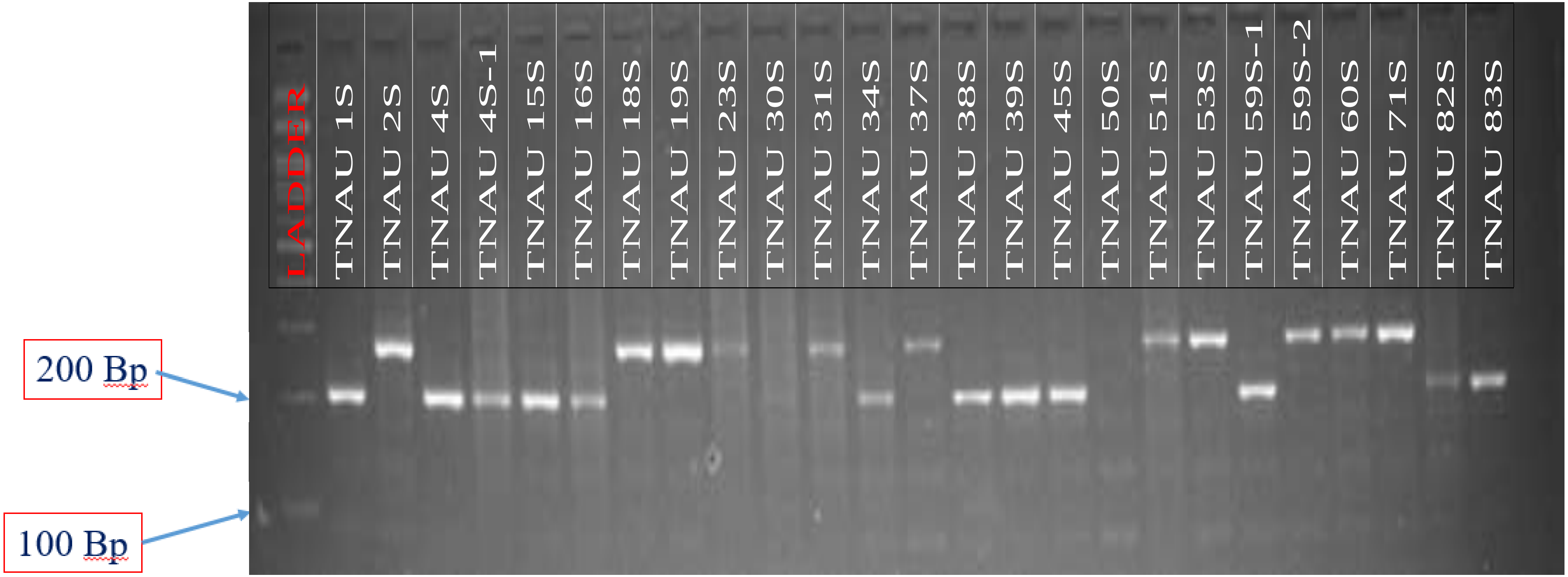

RM521
Chromosome-2

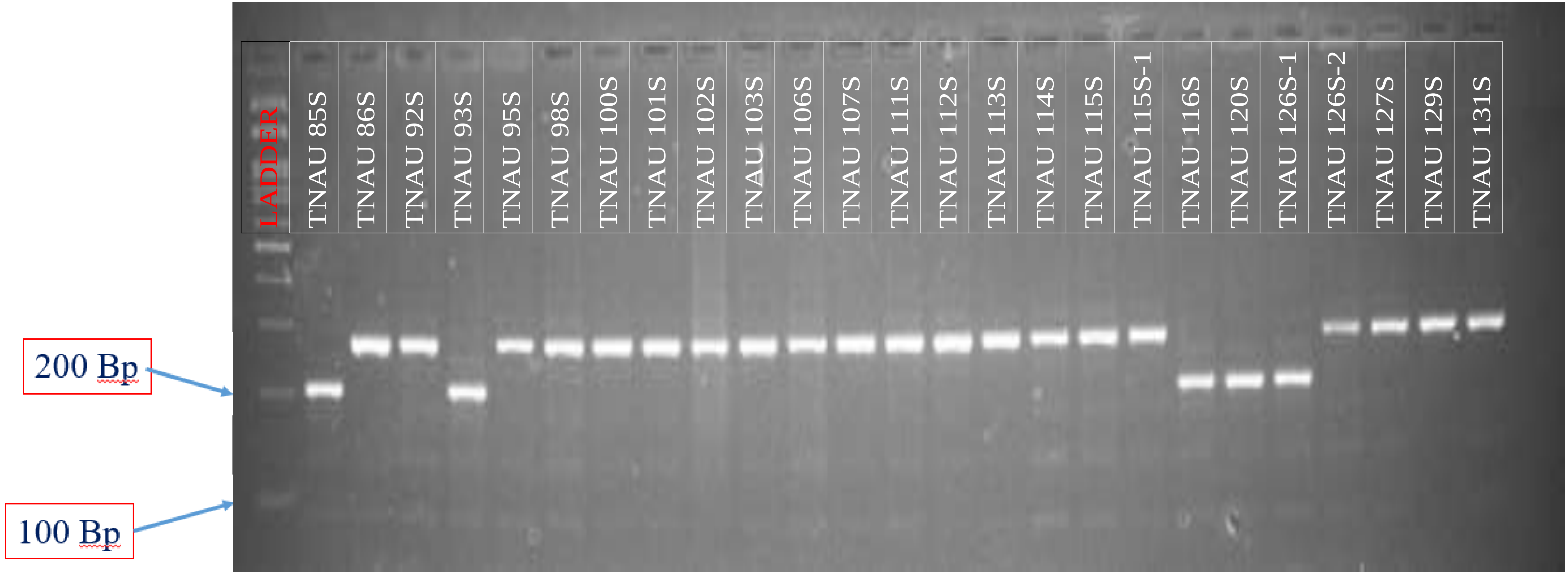

RM521
Chromosome-2

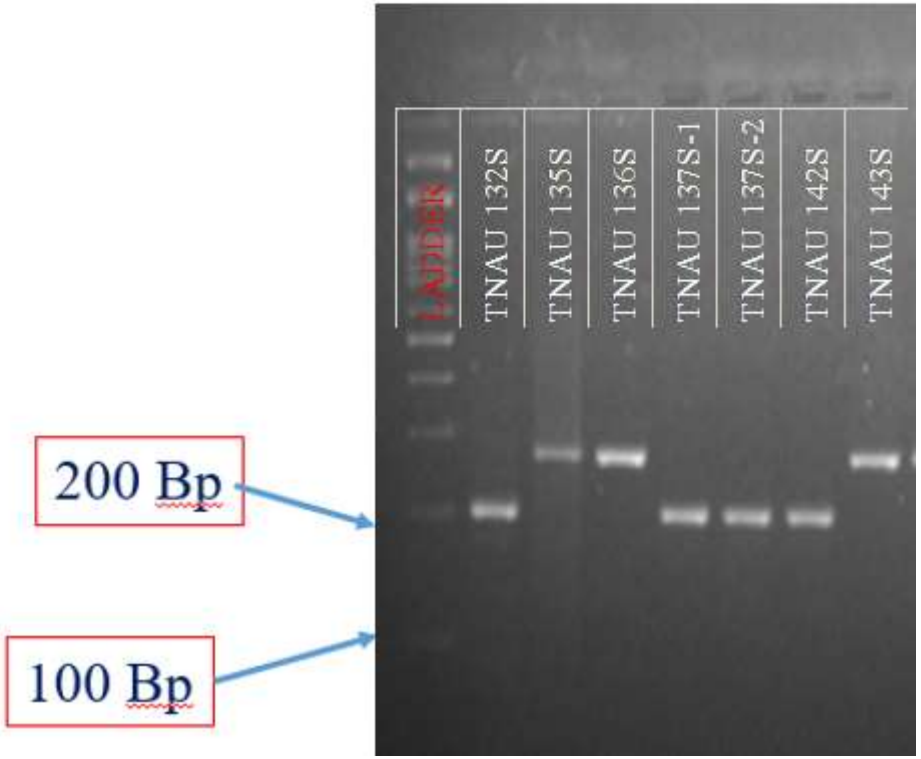

RM129
Chromosome-1

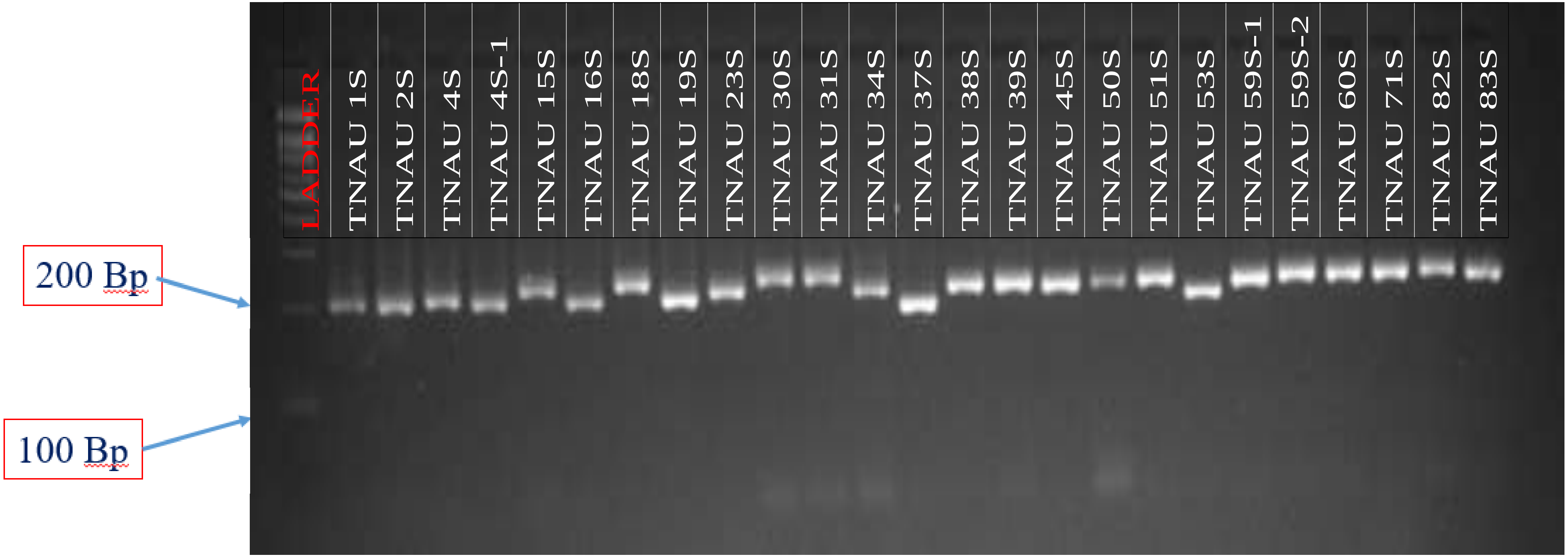

RM129
Chromosome-1

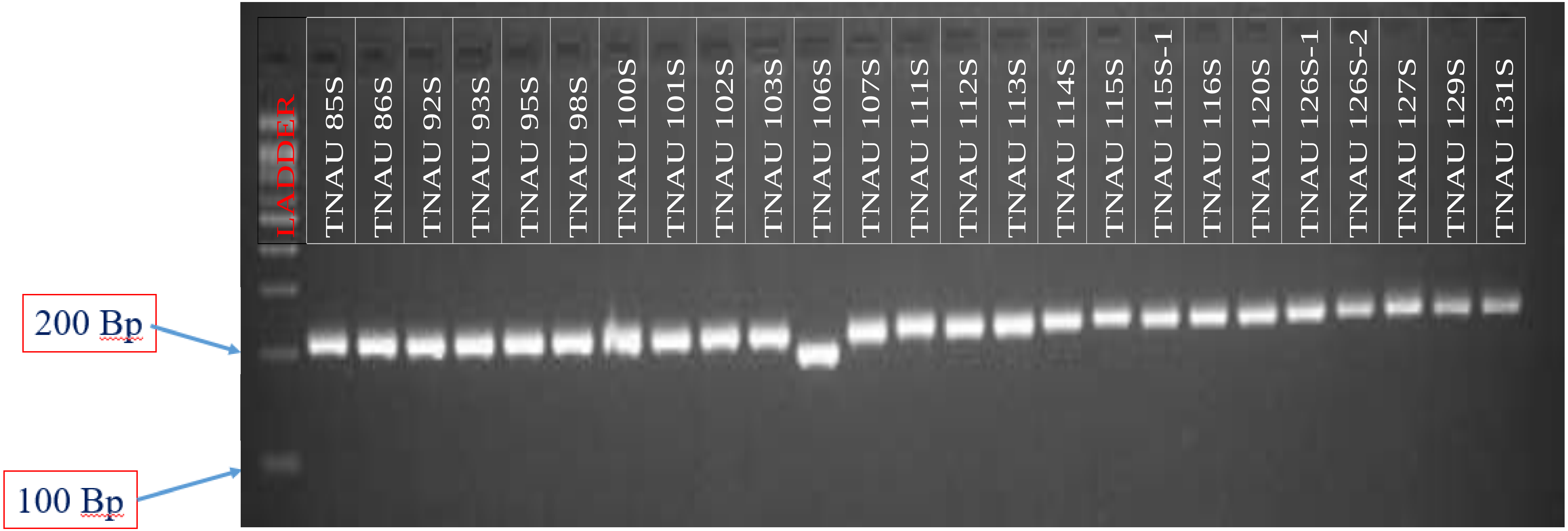

RM129
Chromosome-1

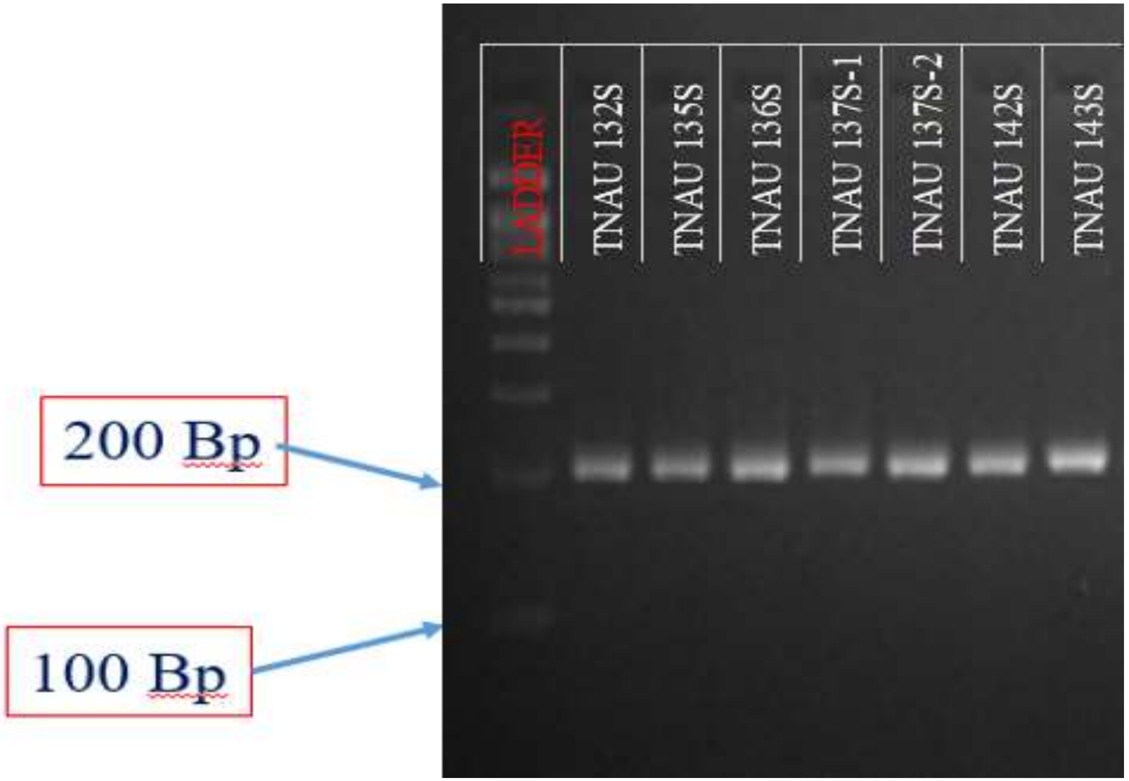

RM335
Chromosome-4

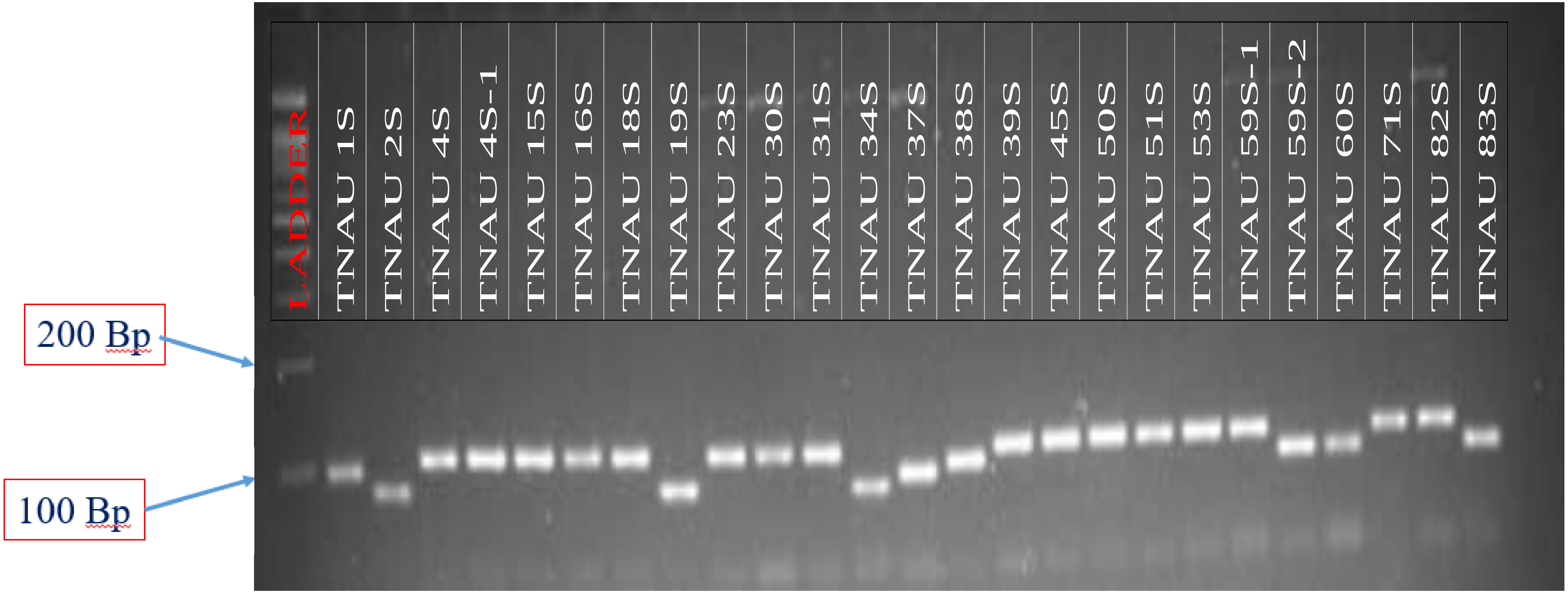

RM335
Chromosome-4

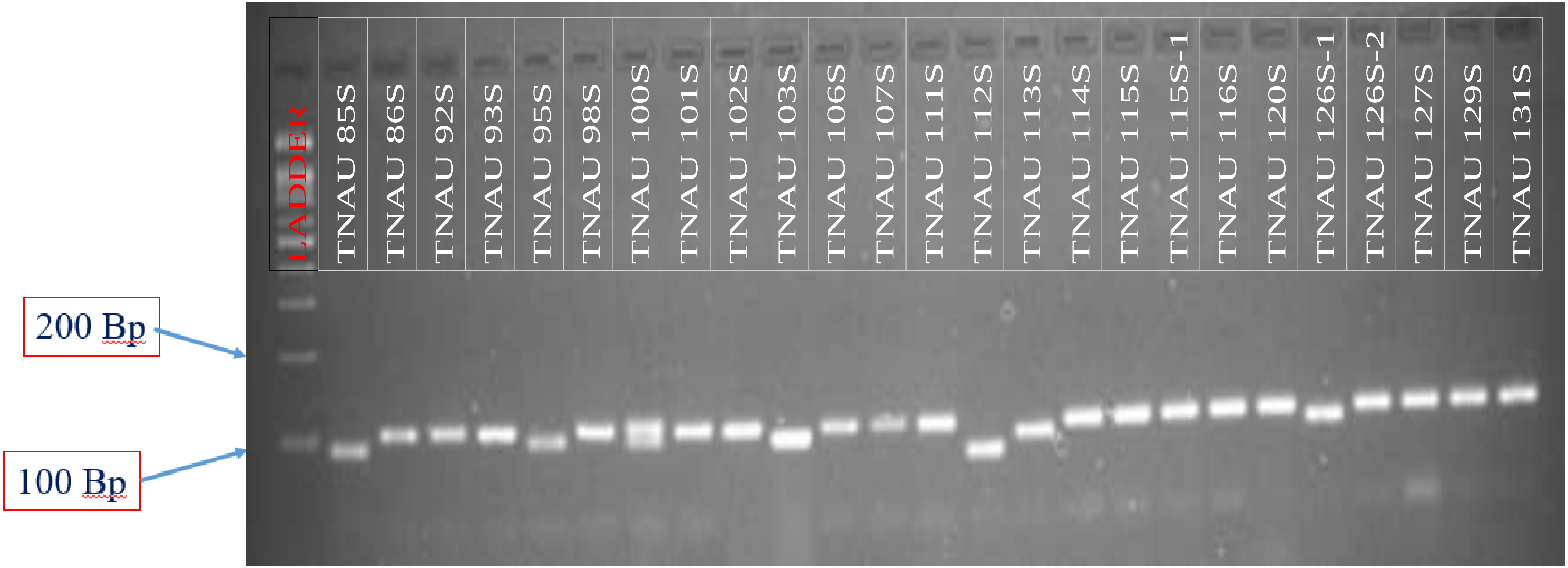

RM335
Chromosome-4

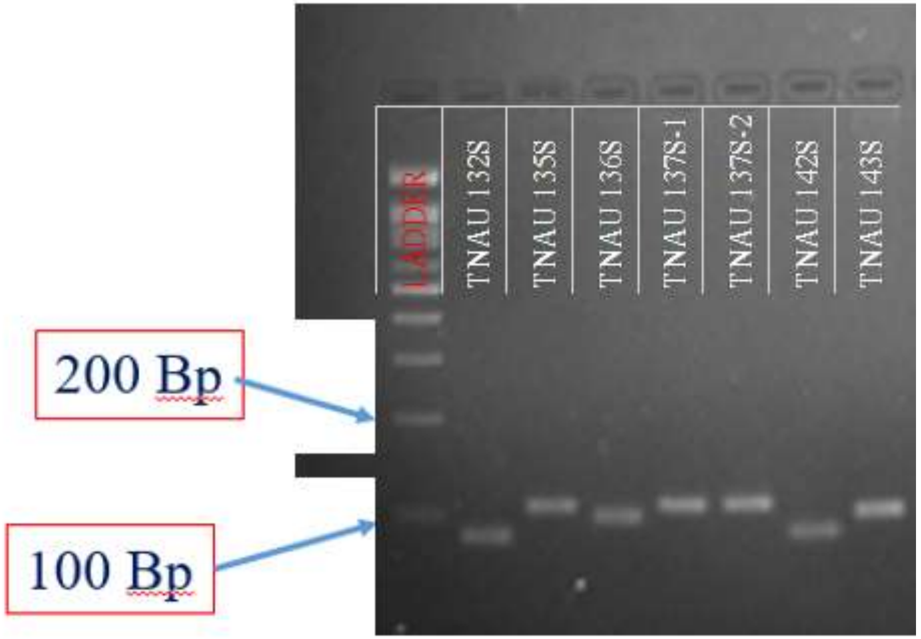

RM457
Chromosome-11

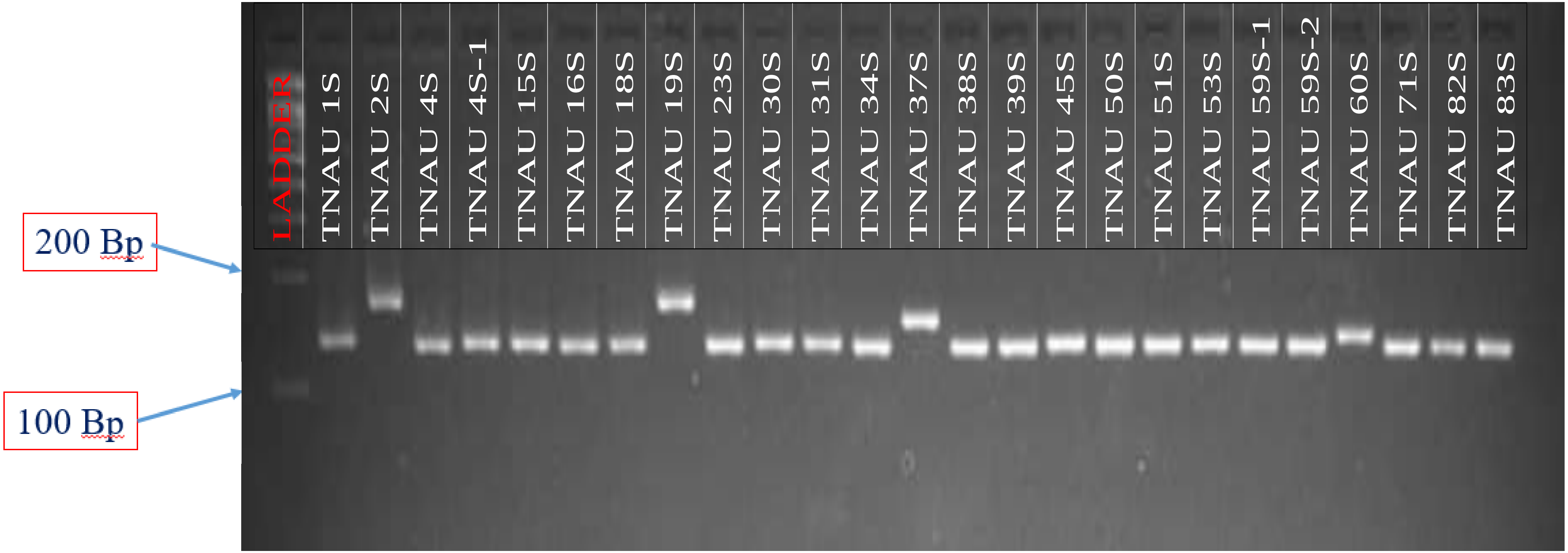

RM457
Chromosome-11

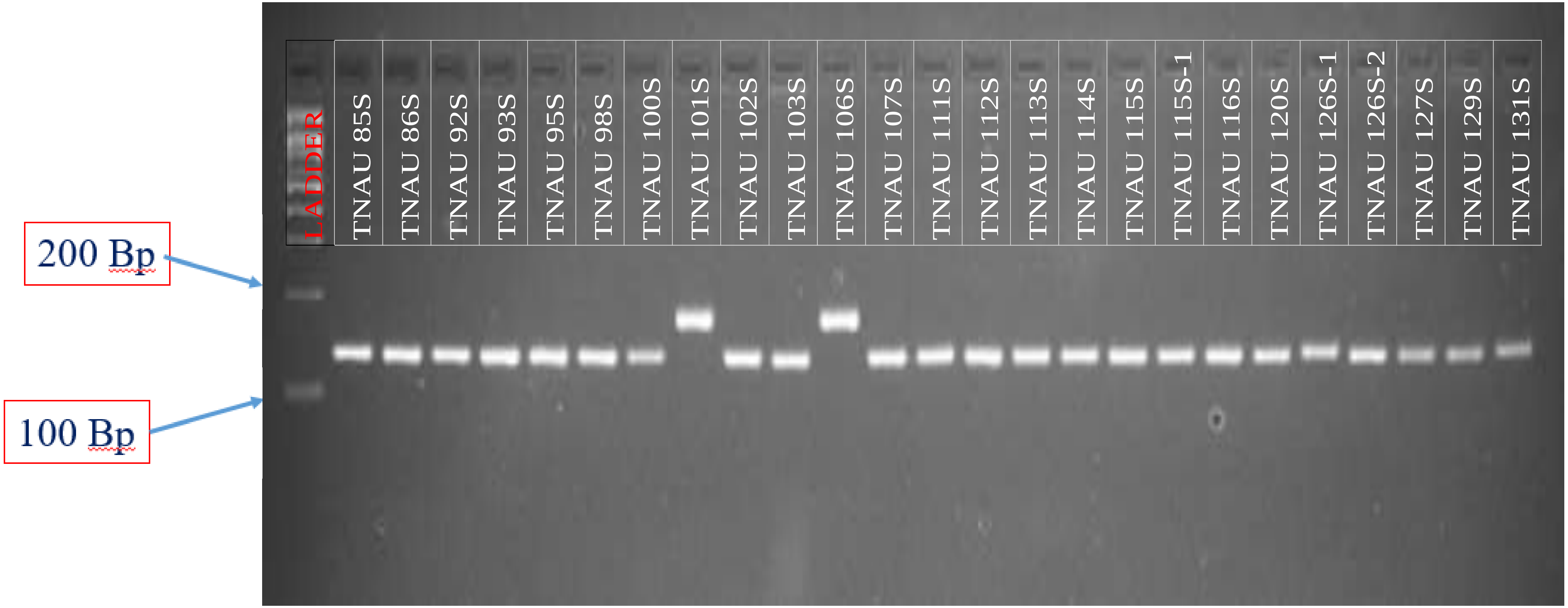

RM457
Chromosome-11

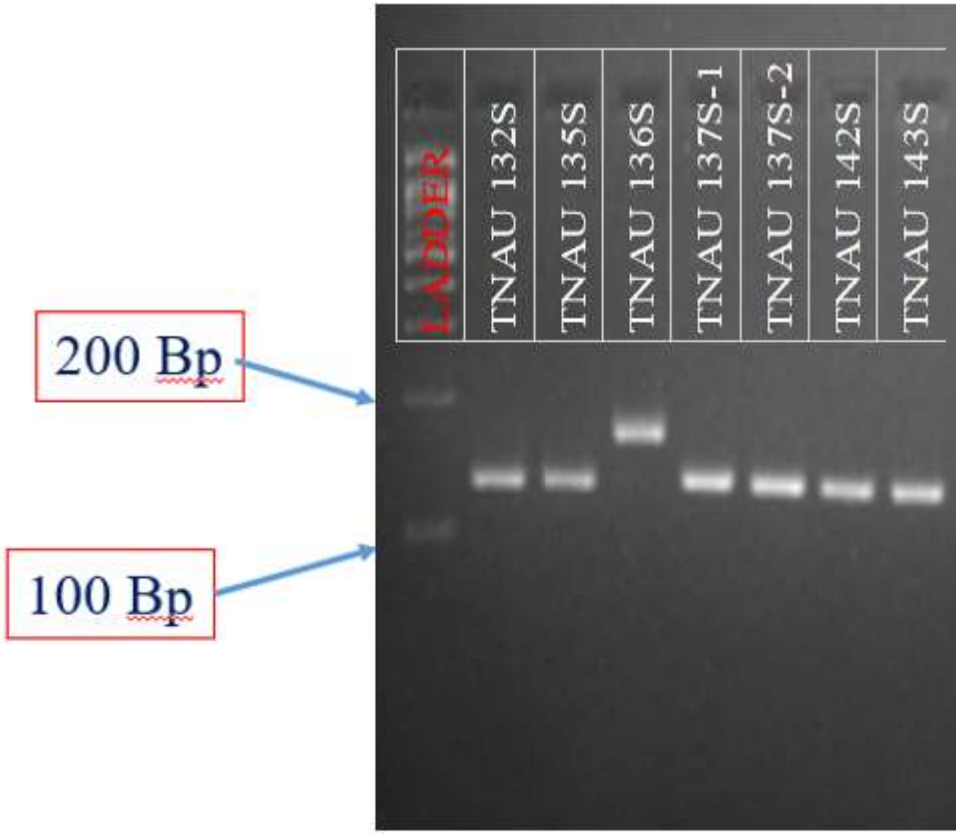

RM427
Chromosome-7

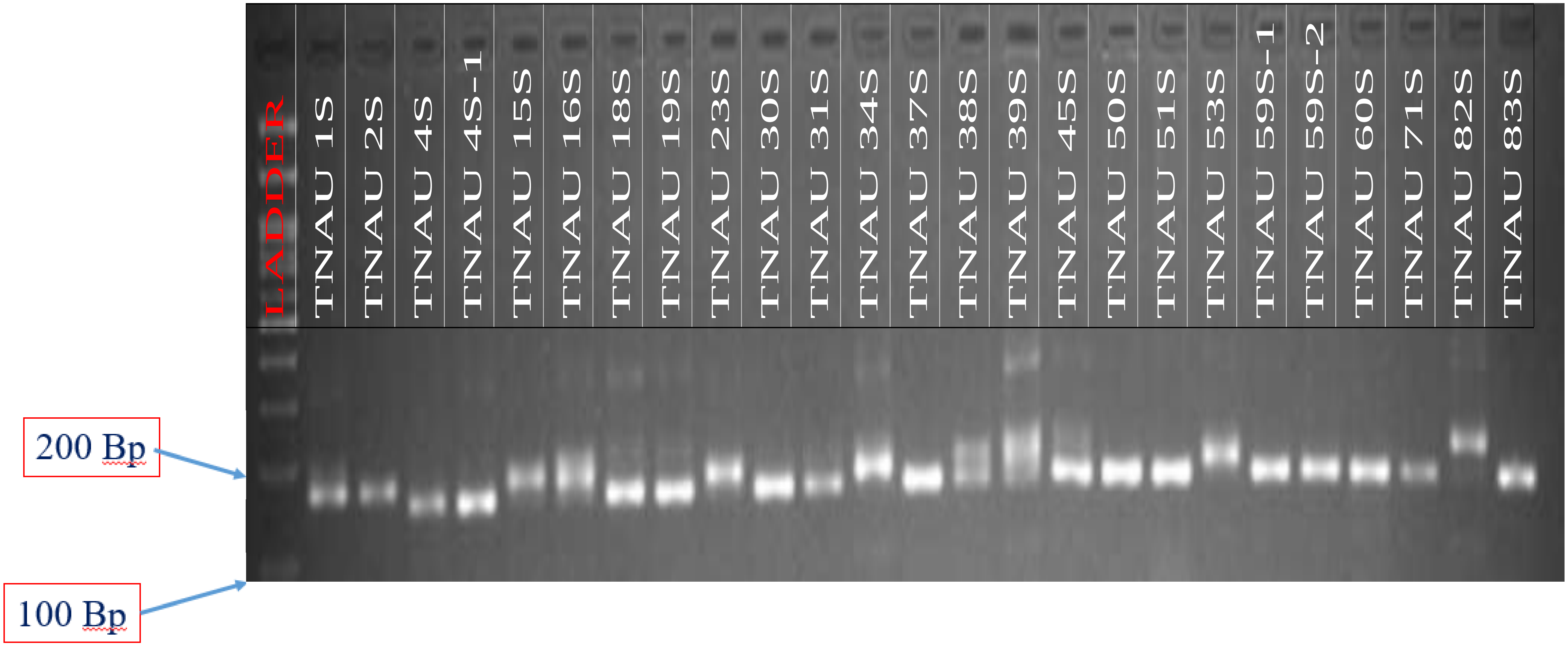

RM427
Chromosome-7

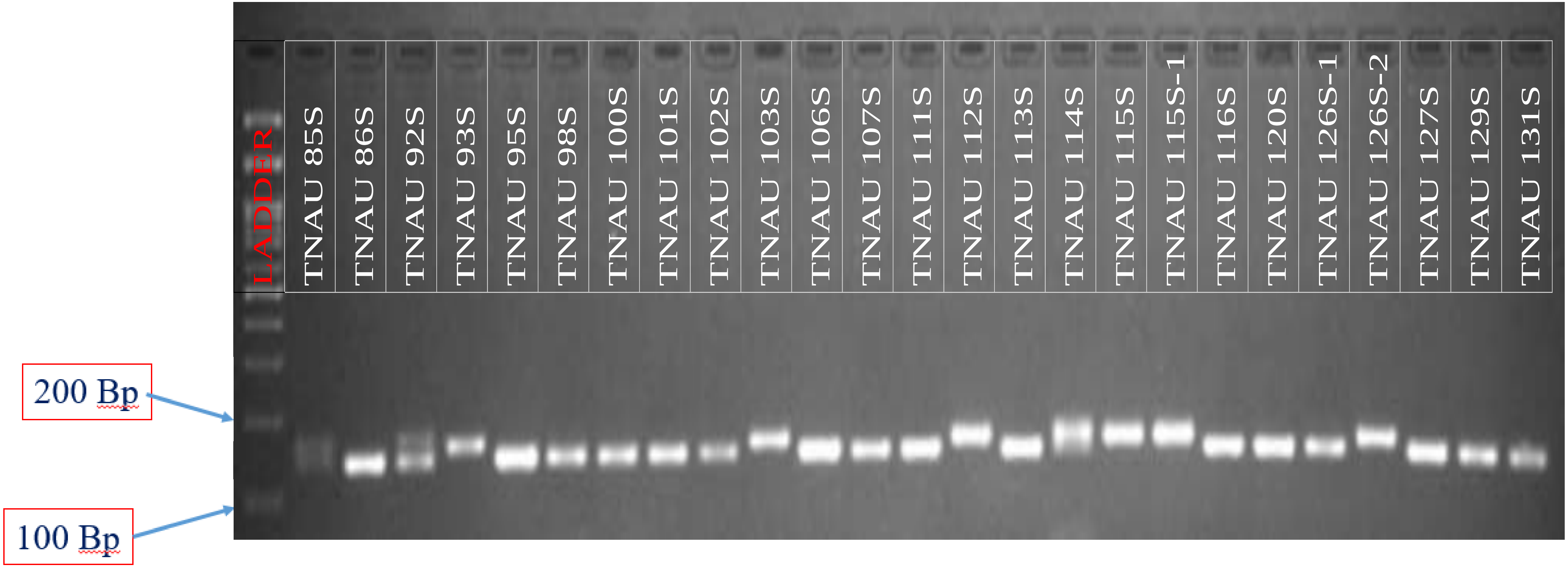

RM427
Chromosome-7

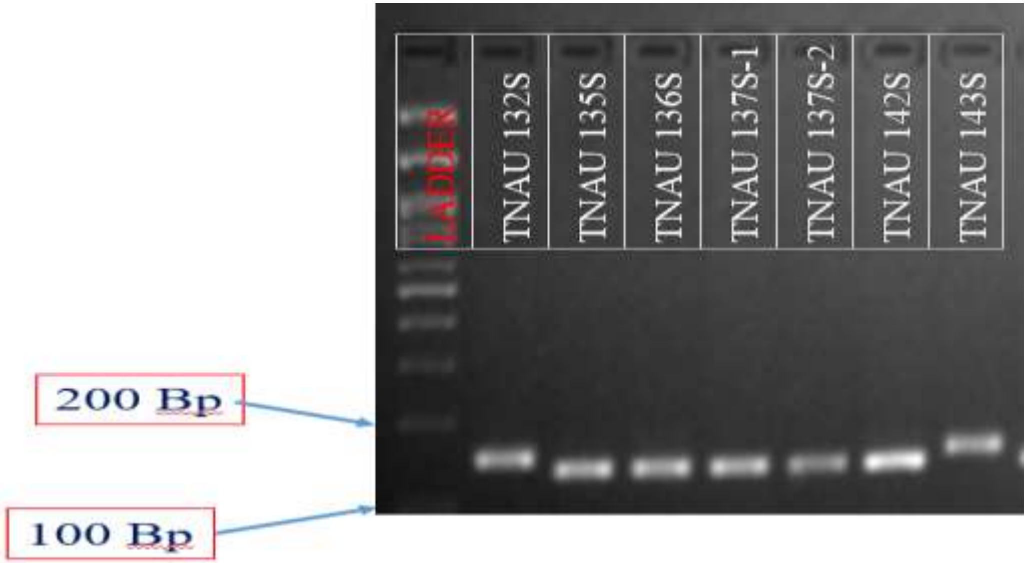

RM205
Chromosome-9

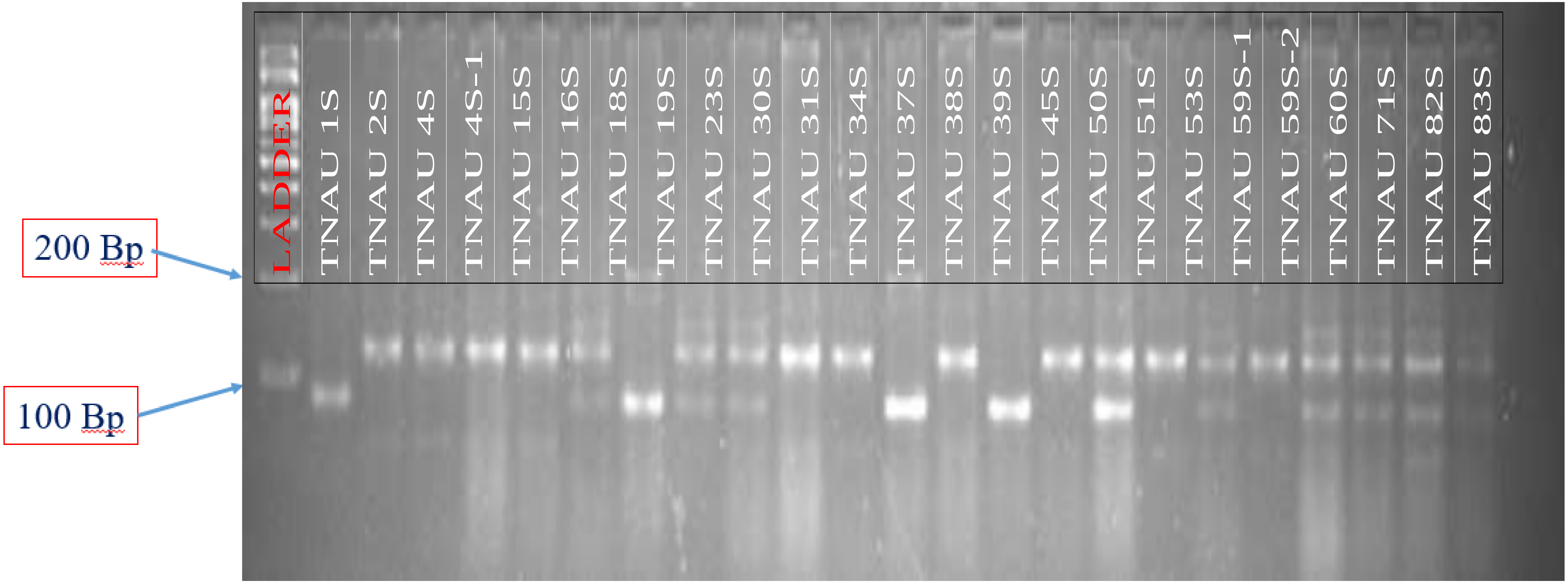

RM205
Chromosome-9

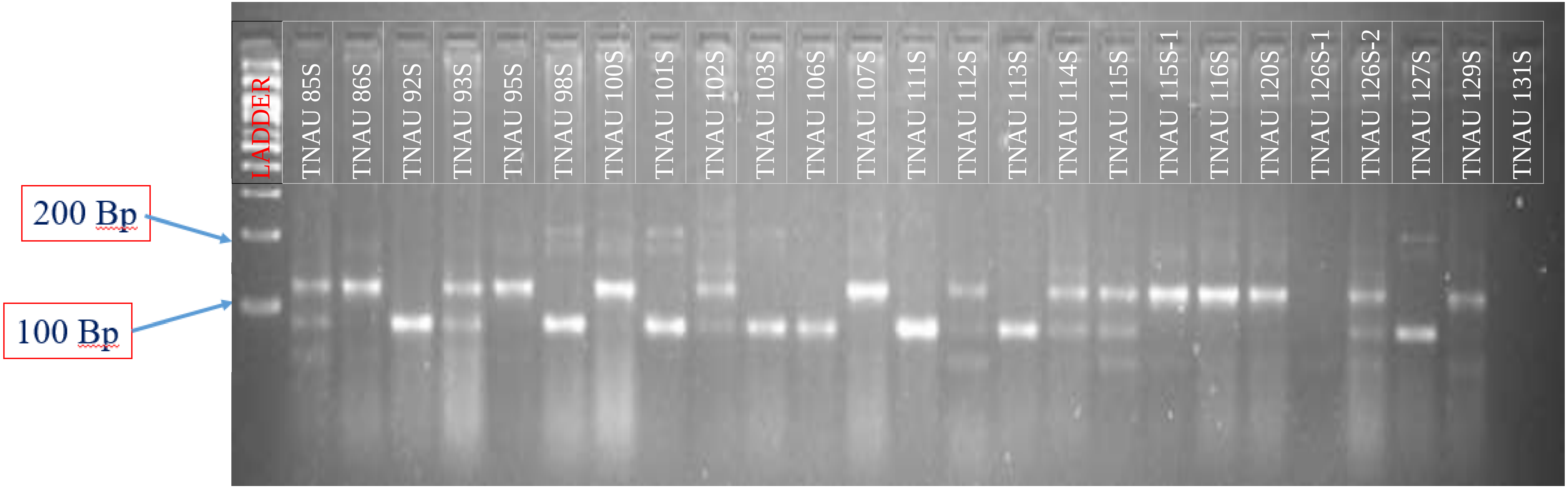

RM205
Chromosome-9

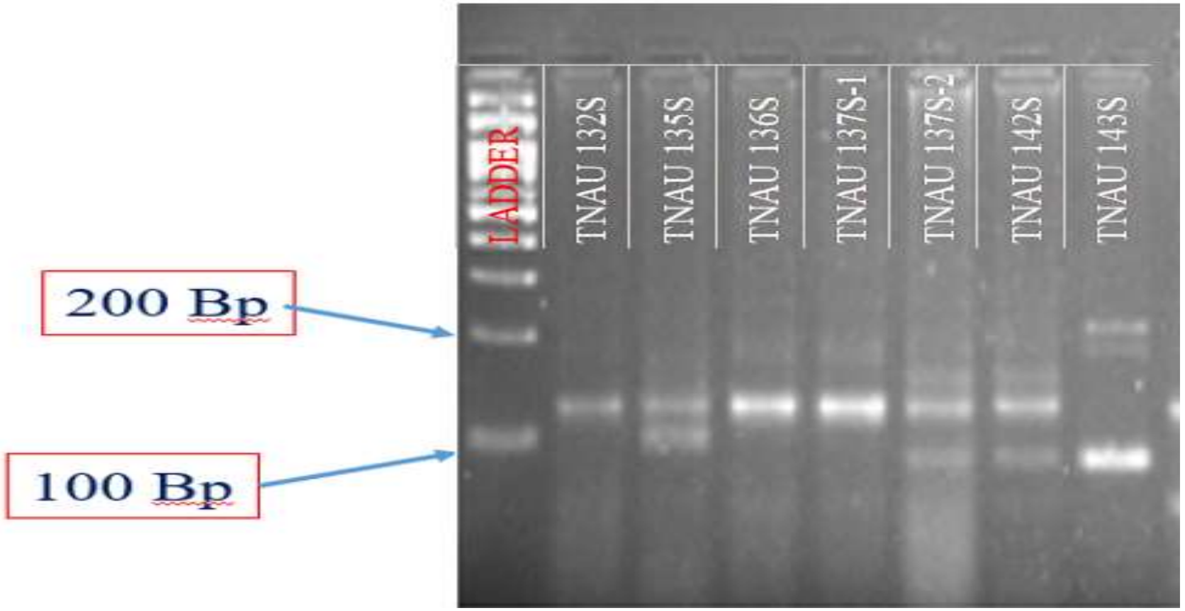

RM8263
Chromosome-7

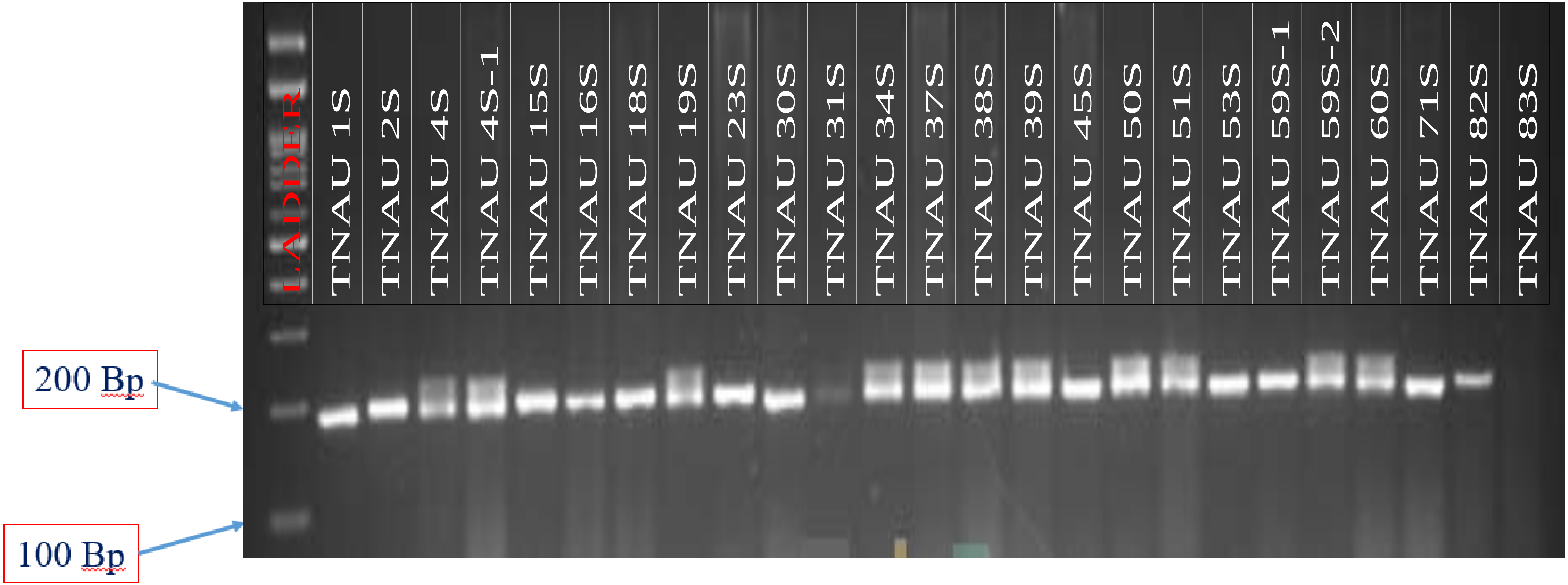

RM8263
Chromosome-7

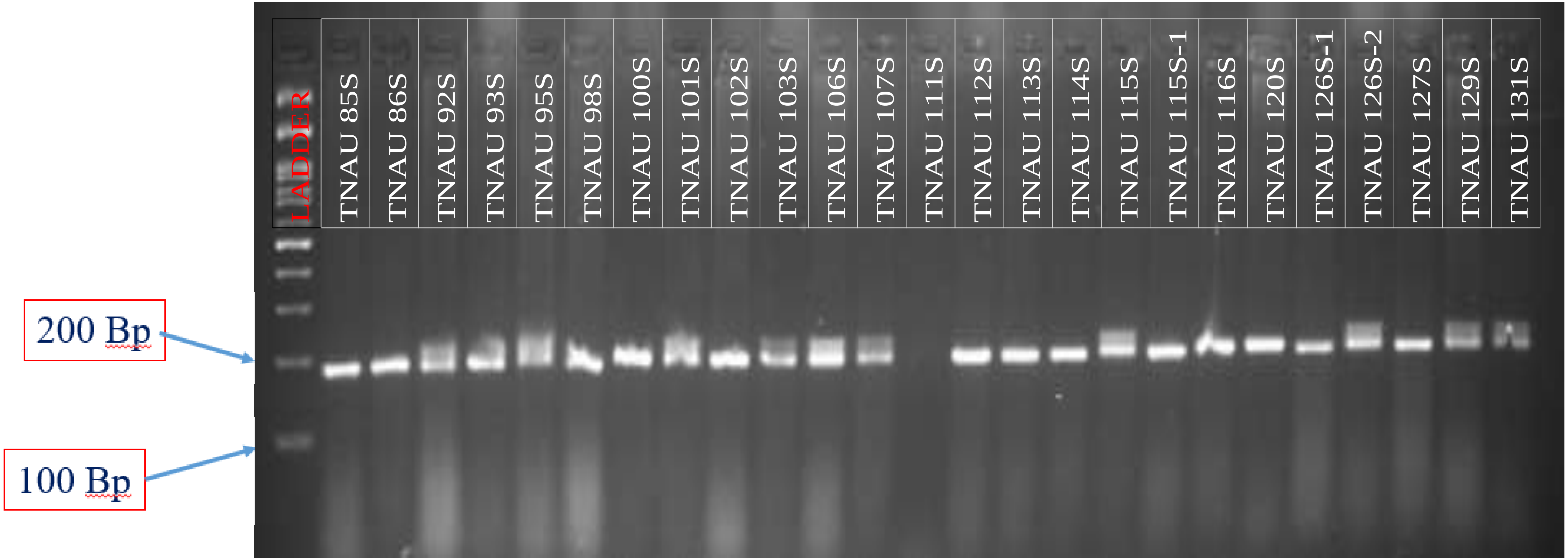

RM8263
Chromosome-7

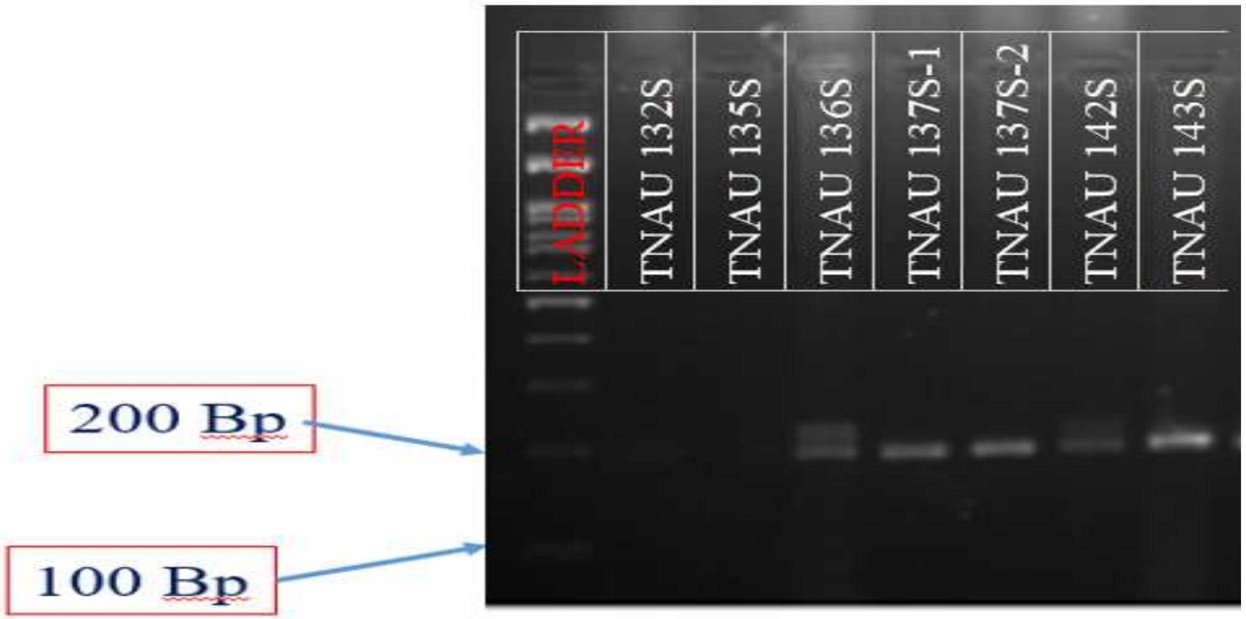

RM1337
Chromosome-12

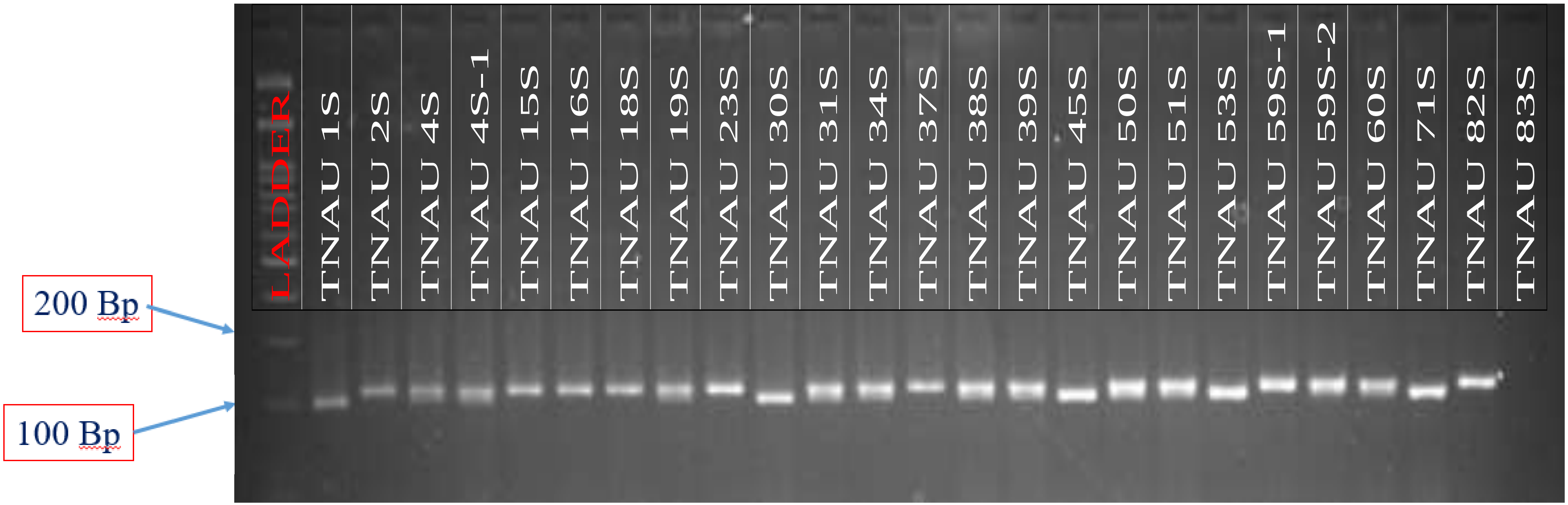

RM1337
Chromosome-12

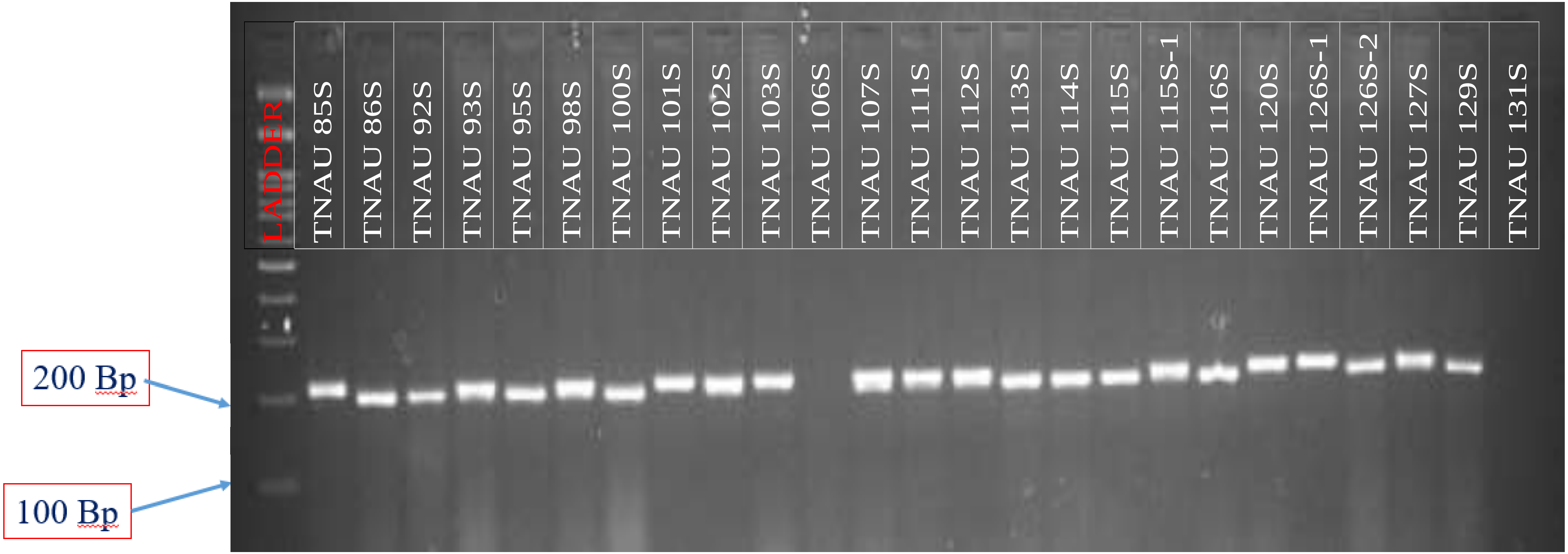

RM1337
Chromosome-12

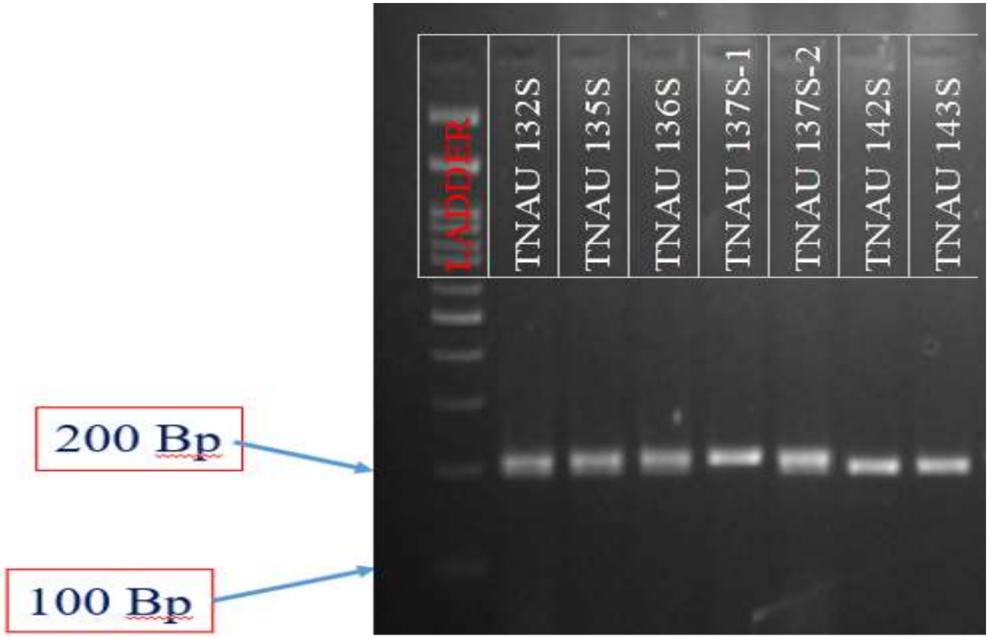

RM346
Chromosome-7

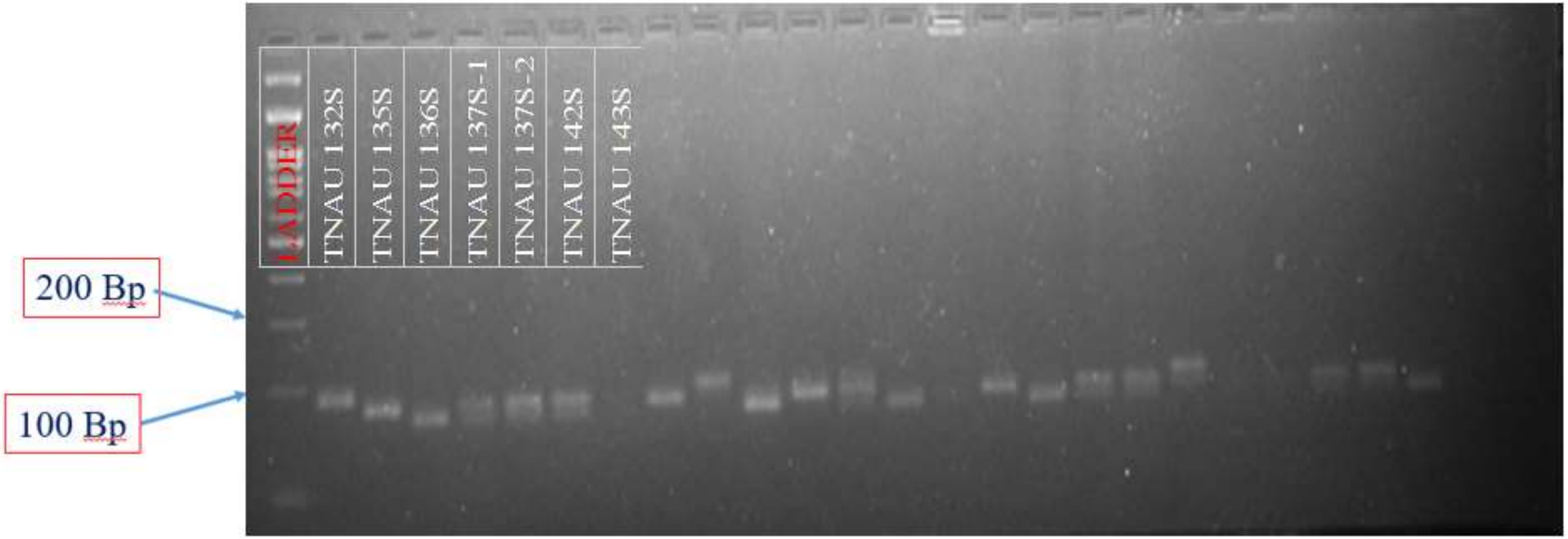

RM346
Chromosome-7

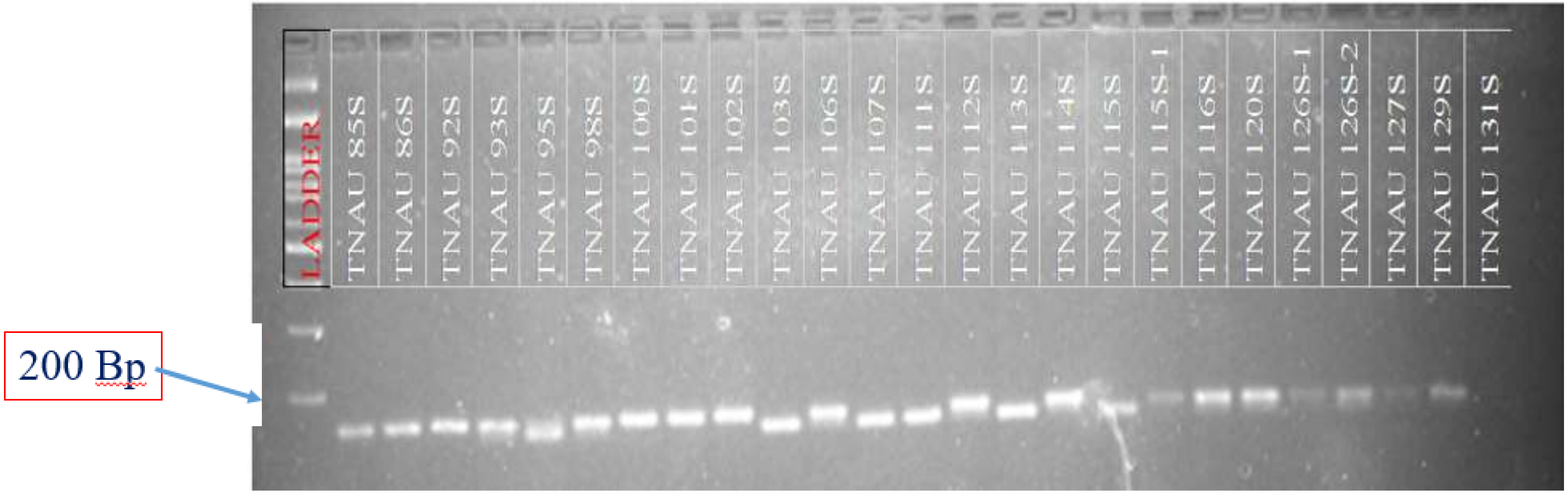

RM346
Chromosome-7

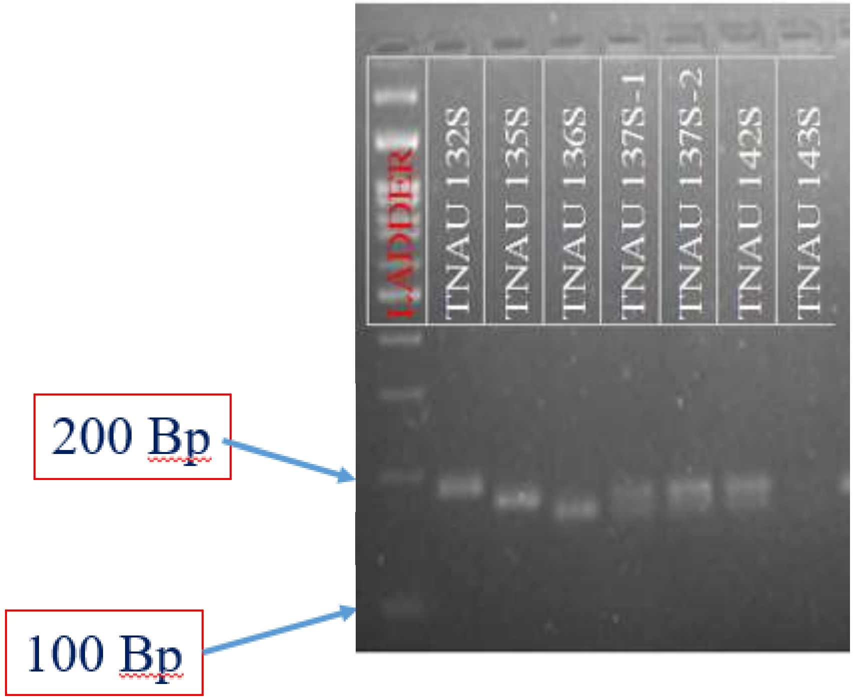

Supplement: Supplemental Information 4 [file peerj-13-18975-s004.pdf]
